# Secular Trends in Musculoskeletal Rehabilitation Needs in 191 Countries and Territories From 1990 to 2019

**DOI:** 10.1001/jamanetworkopen.2021.44198

**Published:** 2022-01-19

**Authors:** Ningjing Chen, Daniel Yee Tak Fong, Janet Yuen Ha Wong

**Affiliations:** 1 School of Nursing, Li Ka Shing Faculty of Medicine, the University of Hong Kong, Hong Kong, China

## Abstract

**Importance:**

It is hard for policy makers and health professionals to develop musculoskeletal rehabilitation strategies because secular trends for musculoskeletal rehabilitation by region and country remain unknown.

**Objective:**

To evaluate the secular trends in global musculoskeletal rehabilitation needs by sex, age, region, country, and health condition.

**Design, Setting, and Participants:**

This cross-sectional study included data from 191 countries and territories from the World Health Organization Rehabilitation Need Estimator between January 1, 1990, and December 31, 2019. Data analyses were performed from February to May 2021.

**Main Outcomes and Measures:**

Prevalence and years lived with disability (YLDs) of musculoskeletal disorders in need of rehabilitation, overall and by sex, age, region, country, and health condition. Trends in rehabilitation needs were evaluated by the estimated annual percentage changes (EAPCs) in age-standardized rates. Pearson correlation analysis was used to examine the associations between EAPCs and the age-standardized rates in 1990. The associations between the age-standardized rates and universal health coverage (UHC) effective coverage index were assessed by fitting a restricted cubic spline in a linear model.

**Results:**

From 1990 to 2019, the global number of prevalent cases of musculoskeletal disorders in need of rehabilitation increased from 1060.6 (95% uncertainty interval [UI], 1009.1-1116.4) million to 1713.6 (95% UI, 1632.4-1800.4) million, with a steady increase in the number of YLDs from 93.9 (95% UI, 67.7-123.6) million to 149.0 (95% UI, 107.5-198.6) million. Overall, 55 countries and territories (28.8%) had annual increase in age-standardized prevalence rates, and 18 countries and territories (9.4%) had annual increase in YLD rates. The global age-standardized prevalence and YLD rates of musculoskeletal disorders decreased annually with EAPCs of 0.34 (95% CI, −0.37 to −0.31) and 0.42 (95% CI, −0.51 to −0.32), respectively. Specifically, the global age-standardized prevalence and YLD rates decreased for low back pain (prevalence: EAPC, −0.52; 95% CI, −0.57 to −0.47; YLD: EAPC, −0.52; 95% CI, −0.66 to −0.37), fractures (prevalence: EAPC, −0.35; 95% CI, −0.41 to −0.30; YLD: EAPC, −0.42; 95% CI, −0.65 to −0.19), other injuries (prevalence: EAPC, −0.75; 95% CI, −0.82 to −0.68; YLD: EAPC, −1.04; 95% CI, −1.38 to −0.71), and amputation (prevalence: EAPC, −0.64; 95% CI, −0.73 to −0.55; YLD: EAPC, −1.13; 95% CI, −1.60 to −0.65). The age-standardized prevalence rate decreased for neck pain (EAPC, −0.10; 95% CI, −0.18 to −0.02) but increased for osteoarthritis (EAPC, 0.13; 95% CI, 0.06-0.19) and rheumatoid arthritis (EAPC, 0.37; 95% CI, 0.04-0.70). In contrast, the age-standardized YLD rates remained stable for neck pain (EAPC, −0.09; 95% CI, −0.35 to 0.16), osteoarthritis (EAPC, 0.14; 95% CI, −0.14 to 0.42), and rheumatoid arthritis (EAPC, 0.38; 95% CI, −0.40 to 1.16). The age-standardized prevalence rate of neck pain decreased faster in male individuals, with an EAPC of −0.10 (95% CI, −0.19 to −0.02), while the age-standardized prevalence rate of low back pain decreased faster in female individuals, with an EAPC of −0.55 (95% CI, −0.60 to −0.51). EAPCs were significantly associated with the baseline age-standardized rates (prevalence: ρ = −0.49; *P* < .001; YLD: ρ = −0.55; *P* < .001), except for those of neck pain and rheumatoid arthritis. Excess rehabilitation needs were observed in countries with both higher and lower UHC effective coverage indexes (prevalence: β = 749.04; SE, 288.52; *P* = .01; YLD: β = 49.70; SE, 22.89; *P* = .03).

**Conclusions and Relevance:**

In this cross-sectional study, substantial increases in musculoskeletal rehabilitation needs were found across locations, suggesting rehabilitation needs have become a global health concern. Mitigating risk factors, strengthening rehabilitation in primary health care, and allocating sufficient funds are encouraged to satisfy rehabilitation needs.

## Key Points

**Question:**

How have musculoskeletal rehabilitation needs changed globally during the past 30 years?

**Findings:**

In this cross-sectional study of 191 countries and territories, prevalent cases and years lived with disability (YLD) counts of musculoskeletal disorders in need of rehabilitation increased substantially, with 55 countries and territories increasing annually in age-standardized prevalence and 18 in YLD rates. The changing patterns of age-standardized prevalence and YLD rates differed by sex and health condition.

**Meaning:**

The findings of this study suggest that musculoskeletal rehabilitation needs have increased, with nearly 1 in every 4 people needing rehabilitation, and coping strategies should be developed.

## Teaser

This cross-sectional analysis evaluates secular trends in global musculoskeletal rehabilitation needs by sex, age, region, country, and health condition.

## Introduction

Globally, musculoskeletal disorders affected nearly 1.3 billion individuals in 2017.^1^ Chronic pain, declines in physical capacity, functional disorders, and low quality of life have been identified as adverse health consequences of musculoskeletal disorders.^2^ This has caused economic losses of billions of dollars globally because of reduced work capacity, early retirement, and increasing medical expenses.^3,4^ In 2016, low back pain and neck pain were ranked first among 154 health conditions in terms of the United States’ medical expenditures, with a value of $134.5 billion, followed by other musculoskeletal disorders ($129.8 billion).^5^

Rehabilitation is effective in reducing pain, building muscle strength, and improving physical mobility and functional ability in individuals with musculoskeletal disorders.^6,7^ In addition, rehabilitation plays a central role in reducing hospitalization and health care costs.^8^ According to the latest report from the World Health Organization (WHO), rehabilitation is a necessary health resource that should be delivered to the entire population, and it is an indispensable part of attaining universal health coverage (UHC).^9^

The heavy burden of musculoskeletal disorders and their health consequences necessitate additional efforts for the appropriate provision of rehabilitation services. However, rehabilitation services are often insufficiently provided in many countries.^9^ This can be explained by the fallback traits of rehabilitation because individuals tend to adopt rehabilitation alternatives only when promotion, prevention, and treatment resolutions are ineffective. Moreover, there is a traditional belief that only individuals with certain disabilities need rehabilitation.^10^ Therefore, it is critical to alter this perspective and track changes in rehabilitation needs to guide rehabilitation resource allocation. However, to the best of our knowledge, only 1 study has comprehensively reported the rehabilitation needs attributed to 25 health conditions, including musculoskeletal disorders, using data from the Global Burden of Diseases, Injuries, and Risk Factors Study (GBD) 2019.^10^ However, the secular trends in musculoskeletal rehabilitation needs by region and country have not been described. In addition, no study has estimated the annual percentage changes in musculoskeletal rehabilitation needs by category over a specific time interval. A better understanding of the secular trends in musculoskeletal rehabilitation needs at the regional and national levels helps to identify the areas with high rehabilitation needs and discover the potential areas requiring additional attention, which will facilitate the development of strategies tailored to rehabilitation needs. Therefore, in this study, we presented the secular trends in prevalence and years lived with disability (YLDs) of musculoskeletal rehabilitation needs by sex, age, region, country, and category between 1990 and 2019 using data from the GBD 2019 study.

## Methods

### Overview

According to the Hong Kong Hospital Authority’s Investigator’s Code of Practice, ethical approval was not needed because this study conducted secondary analyses of publicly available data and participants’ information was not individually identifiable. This study followed the Strengthening the Reporting of Observational Studies in Epidemiology (STROBE) reporting guideline for cross-sectional studies. The GBD 2019 study comprehensively analyzed causes of 369 diseases and injuries and 87 risk factors in 204 countries and territories between 1990 to 2019 by using data from population censuses, household surveys, civil registration and vital systems, hospital and clinical records, and other sources, which were collected from published literature, institutional websites, international and national surveys, and the GBD collaborative network.^11,12^ As a coping strategy to deal with physical, psychological, and social dysfunction, rehabilitation is needed at some stages of diseases and injuries.^10^ Importantly, for estimating musculoskeletal rehabilitation needs, published studies that described or calculated rehabilitation needs in populations with health conditions were reviewed without language restrictions. However, only those population-based original studies with a large sample (ie, ≥150 participants) and showing national representative results were included.^10^

### Data Source

This cross-sectional study used data on prevalent cases, YLD counts, and age-standardized prevalence and YLD rates of musculoskeletal rehabilitation needs in 191 countries and territories between January 1, 1990, and December 31, 2019, from the WHO Rehabilitation Need Estimator.^13^ Overall, 102 countries and territories had prevalence data inputs for low back pain; 26 for neck pain; 23 for hip osteoarthritis; 25 for knee osteoarthritis; 40 for hand osteoarthritis; 1 for other osteoarthritis; and 42 for rheumatoid arthritis.^10^ For fractures, amputation, and other injuries, prevalence estimates were calculated using incidence data inputs, which were classified by cause in the GBD 2019 study^10,11^ and are shown in eTable 1 in the Supplement. The number of countries with incidence data inputs by cause ranged from 6 to 75. Data inputs for YLD estimates resembled those for prevalence estimates for each health condition.^10^ Estimated rehabilitation needs (ie, prevalence and YLD estimates) by age, sex, region, country, and year were calculated for 191 countries and territories by using the GBD’s Bayesian meta-regression tool, Disease Modeling–Metaregression (DisMod-MR) version 2.1.^10^ Briefly, DisMod-MR 2.1 was used to fit a global model on data from all countries and territories.^14^ The global model accounted for extra covariance within each of 7 GBD super-regions (eTable 2 in the Supplement) by random effects and adjusted for sex and country. Based on the adjusted global model, estimates for 1 of 7 super-regions, 1 of 21 regions (eTable 2 in the Supplement), and a single country or territory were then obtained.^14,15^ In the GBD 2019 study, the nonweighted mean of the age structure for a country or territory with a population greater than 5 million was used to calculate the standard age distribution,^11,16^ which is also presented in eTable 3 in the Supplement. In addition, prevalence and YLD estimates were derived by sampling 1000 draws. The corresponding 95% uncertainty interval (UI) was the 25th and 975th value of these 1000 ordered draws.^10^

Musculoskeletal disorders included 7 health conditions: low back pain, neck pain, fractures, other injuries, osteoarthritis, amputation, and rheumatoid arthritis. These conditions were selected because they contributed to the highest number of YLDs and rehabilitation was the necessary and key intervention.^10^ From the GBD 2019 study, we also obtained the UHC effective coverage index (eTable 4 in the Supplement), which is a composite indicator representing promotion, prevention, and treatment health services.^17^ The UHC effective coverage index value ranges from 0 to 100, and a greater value reflects a better performance in delivering health services to the population.^17^

### Statistical Analysis

The changing patterns of age-standardized prevalence and YLD rates of rehabilitation needs were compared in World Bank high-income countries and low- and middle-income countries in the 6 WHO regions, including Africa, the Americas, Southeast Asia, Europe, the Eastern Mediterranean, and the Western Pacific regions.^10^ Based on previous studies,^18,19^ we used the estimated annual percentage changes (EAPCs) in age-standardized rates to measure and quantify the trends of rehabilitation needs. The lower bound of an EAPC greater than 0 suggests an increasing trend, and the upper bound of an EAPC smaller than 0 indicates a decreasing trend. However, 95% CIs of an EAPC that include 0 suggest the age-standardized rate has remained relatively stable during the study period. We mapped the age-standardized rates and EAPCs in musculoskeletal rehabilitation needs for each country and territory. Subsequently, sex and age differences in global musculoskeletal rehabilitation needs were compared. The associations between EAPCs and the age-standardized rates of musculoskeletal rehabilitation needs in 1990 were then examined by ρ coefficients in Pearson correlation analysis to determine the potential factor of EAPCs.^19,20^ Furthermore, given that health spending per capita (eTable 4 in the Supplement) was positively associated with UHC effective coverage index,^17^ we fitted a restricted cubic spline in a linear model to examine the associations between the age-standardized rates of musculoskeletal rehabilitation needs in 2019 and UHC effective coverage index after adjusting for health spending per capita.^17,21^ The models were fitted with 3, 4, 5, and 6 knots, and the model with 4 knots was selected given the highest adjusted *R*^2^ and smallest residual standard error. All data analyses were conducted in RStudio version 1.3.1093 (R Project for Statistical Computing), and a 2-sided *P* < .05 was considered statistically significant. Data analyses were performed from February to May 2021.

## Results

### Prevalence and YLD Estimates

**Figure 1.**
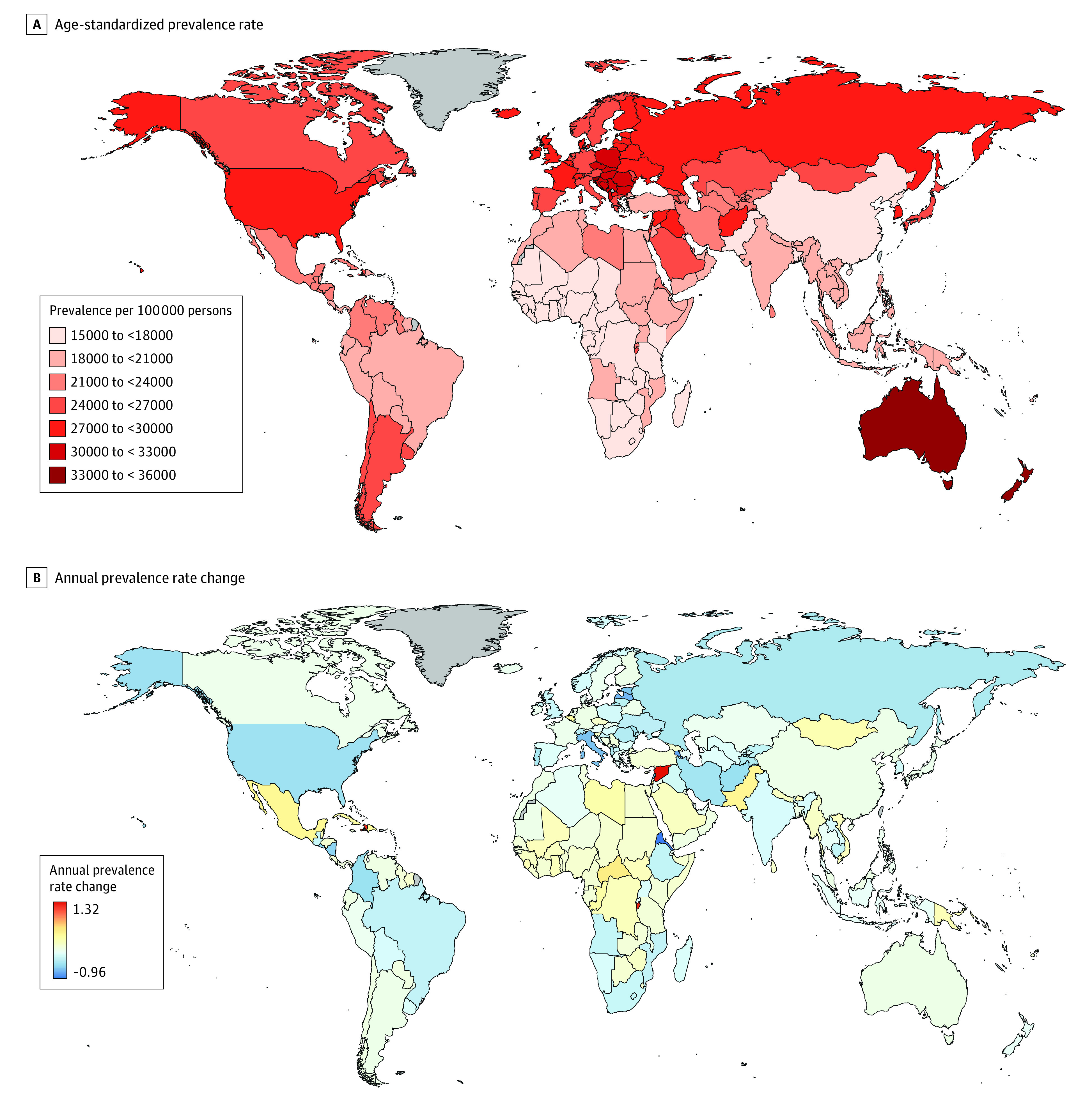
Prevalence Estimates for Musculoskeletal Rehabilitation Needs Worldwide Areas shaded gray did not have available data.

Globally, the number of prevalent cases of musculoskeletal disorders in need of rehabilitation increased significantly from 1060.6 (95% UI, 1009.1-1116.4) million in 1990 to 1713.6 (95% UI, 1632.4-1800.4) million in 2019, which contributed to a steady increase in the number of YLDs from 93.9 (95% UI, 67.7-123.6) million in 1990 to 149.0 (95% UI, 107.5-198.6) million in 2019. The global age-standardized prevalence and YLD rates of musculoskeletal disorders in need of rehabilitation decreased annually during the entire period with EAPCs of −0.34 (95% CI, −0.37 to −0.31) and −0.42 (95% CI, −0.51 to −0.32), respectively. The Western Pacific region had the largest increases in the prevalent cases and YLDs. Despite this, the highest rehabilitation needs were in the World Bank high-income countries in 2019. Overall, the age-standardized rates of rehabilitation needs decreased across all regions during this period annually. The fastest decreasing speeds were noted in Europe, with EAPCs of −0.47 (95% CI, −0.50 to −0.45) and −0.57 (95% CI, −0.65 to −0.48) in age-standardized prevalence and YLD rates, respectively (Table). At the national level, in 2019, the highest age-standardized prevalence rates per 100 000 persons were observed in New Zealand (35 498.2; 95% UI, 33 899.9-37 469.3), followed by Australia (33 348.4; 95% UI, 31 664.9-35 247.8) and Slovenia (33 030.3; 95% UI, 31 511.7-34 693.5) (Figure 1A; eTable 5 in the Supplement). The age-standardized prevalence rates increased in 55 countries and territories, with the Syrian Arab Republic showing the fastest growth rate, with an EAPC of 1.32 (95% CI, 1.29-1.35) per year (Figure 1B). In contrast, the highest age-standardized YLD rates per 100 000 persons existed in Afghanistan (2930.1; 95% UI, 2009.6-4392.8), the United States (2856.5; 95% UI, 2067.5-3783.0), and Denmark (2593.9; 95% UI, 1849.2-3441.0) (eFigure 1A and eTable 6 in the Supplement). Among 18 countries and territories with increases in age-standardized YLD rates, Burundi increased fastest, with an EAPC of 1.38 (95% CI, 1.29-1.46) (eFigure 1B in the Supplement).

**Table.** Musculoskeletal Rehabilitation Needs in 2019 and Trends Between 1990 and 2019 by World Health Organization Region

Region	Prevalence			YLDs		
	No. (95% UI)	ASRs per 100 000 persons (95% UI)	EAPC (95% CI)	No. (95% UI)	ASRs per 100 000 persons (95% UI)	EAPC (95% CI)
World Bank high-income countries	440 888 942 (421 545 909 to 461 006 107)	27 454.1 (26 198.7 to 28 814.1)	−0.31 (−0.33 to −0.28)	39 796 477 (28 491 329 to 53 575 691)	2404.6 (1722.5 to 3196.5)	−0.28 (−0.36 to −0.19)
Western Pacific	426 725 952 (400 919 594 to 454 208 920)	17 699.4 (16 670.3 to 18 824.2)	−0.06 (−0.09 to −0.03)	37 626 387 (26 820 254 to 51 100 520)	1557.8 (1113.6 to 2097.2)	−0.27 (−0.38 to −0.17)
Southeast Asia	369 339 406 (351 639 924 to 388 490 686)	19 107.5 (18 245.8 to 20 036.0)	−0.22 (−0.25 to −0.19)	32 638 938 (23 537 446 to 42 986 130)	1699.8 (1229.8 to 2238.2)	−0.41 (−0.50 to −0.31)
Europe	302 298 953 (289 346 326 to 316 450 308)	26 924.9 (25 727.8 to 28 206.2)	−0.47 (−0.50 to −0.45)	25 137 083 (18 097 385 to 33 543 852)	2203.7 (1580.1 to 2932.5)	−0.57 (−0.65 to −0.48)
Eastern Mediterranean	118 709 853 (111 157 257 to 127 159 399)	20 164.3 (18 985.9 to 21 498.0)	0.02 (−0.01 to 0.05)	10 533 856 (7 593 119 to 13 687 920)	1801.6 (1301.4 to 2346.5)	−0.11 (−0.21 to −0.01)
The Americas	226 449 897 (215 059 745 to 238 113 512)	21 558.6 (20 464.8 to 22 666.9)	−0.14 (−0.16 to −0.11)	18 176 795 (13 055 388 to 24 196 147)	1728.5 (1242.8 to 2298.1)	−0.18 (−0.28 to −0.08)
Africa	129 105 085 (121 518 605 to 137 027 944)	17 474.3 (16 530.4 to 18 491.2)	−0.07 (−0.10 to −0.04)	11 260 629 (8 105 431 to 14 865 745)	1548.5 (1118.3 to 2050.3)	−0.09 (−0.19 to 0.02)

Abbreviations: ASRs, age-standardized rates; EAPC, estimated annual percentage change; UI, uncertainty interval; YLDs, years lived with disability.

### Musculoskeletal Rehabilitation Needs by Category

**Figure 2.**
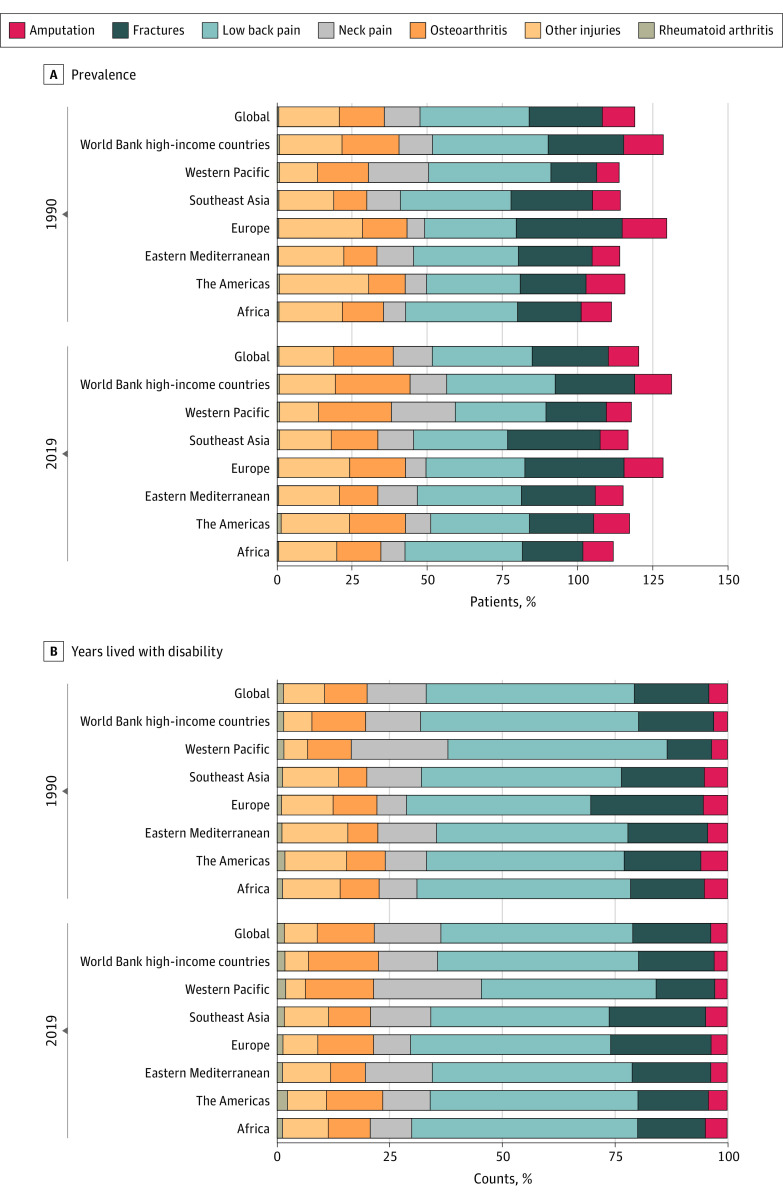
Proportion of Prevalent Cases and Years Lived With Disability Counts of Musculoskeletal Disorders A, Given that an individual may have more than 1 health condition, the total proportion is larger than 100%.

Based on the proportion of prevalent cases and YLD counts, low back pain contributed most to musculoskeletal rehabilitation needs over the past 3 decades across regions. In 2019, low back pain accounted for 568 444 532 of 1 713 557 313 global prevalent cases of musculoskeletal disorders (33.2%), followed by fractures (436 288 796 [25.5%]), osteoarthritis (343 943 653 [20.1%]), other injuries (305 001 551 [17.8%]), neck pain (222 718 453 [13.0%]), amputation 175 731 751 [10.3%]), and rheumatoid arthritis (13 487 835 [0.8%]) (Figure 2A). From 1990 to 2019, the global age-standardized rate of musculoskeletal disorders by category did not follow the same temporal trends. For age-standardized prevalence rates, increasing trends were found for osteoarthritis (EAPC, 0.13; 95% CI, 0.06-0.19) and rheumatoid arthritis (EAPC, 0.37; 95% CI, 0.04-0.70). In contrast, decreasing trends were found for low back pain (EAPC, −0.52; 95% CI, −0.57 to −0.47), neck pain (EAPC, −0.10; 95% CI, −0.18 to −0.02), fractures (EAPC, −0.35; 95% CI, −0.41 to −0.30), other injuries (EAPC, −0.75; 95% CI, −0.82 to −0.68), and amputation (EAPC, −0.64; 95% CI, −0.73 to −0.55). For age-standardized YLD rates, decreasing trends were found for low back pain (EAPC, −0.52; 95% CI, −0.66 to −0.37), fractures (EAPC, −0.42; 95% CI, −0.65 to −0.19), other injuries (EAPC, −1.04; 95% CI, −1.38 to −0.71), and amputation (EAPC, −1.13; 95% CI, −1.60 to −0.65), whereas age-standardized YLD rates remained stable for neck pain (EAPC, −0.09; 95% CI, −0.35 to 0.16), osteoarthritis (EAPC, 0.14; 95% CI, −0.14 to 0.42), and rheumatoid arthritis (EAPC, 0.38; 95% CI, −0.40 to 1.16) (eTable 7 in the Supplement). Both age-standardized rates and EAPCs of musculoskeletal disorders by category and country are presented in eFigures 2 to 15 in the Supplement.

### Sex and Age Patterns

From 1990 to 2019, age-standardized prevalence and YLD rates of low back pain, other injuries, and amputation in need of rehabilitation showed steady declining trends, whereas those of neck pain, fractures, osteoarthritis, and rheumatoid arthritis largely stabilized. Notably, age-standardized rates due to neck pain, fractures, other injuries, and amputation increased steadily overall from 2014 to 2019. The age-standardized prevalence and YLD rates of fractures, other injuries, and amputation were higher in male individuals, whereas those of low back pain, neck pain, osteoarthritis, and rheumatoid arthritis were higher in female individuals (eFigure 16 and eFigure 17 in the Supplement). The age-standardized prevalence rates of neck pain, fractures, other injuries, and amputation decreased faster in male individuals, with EAPCs of −0.10 (95% CI, −0.19 to −0.02), −0.38 (95% CI, −0.43 to −0.33), −0.82 (95% CI, −0.88 to −0.76), and −0.71 (95% CI, −0.79 to −0.64), respectively. In contrast, the age-standardized prevalence rate of low back pain decreased faster in female individuals, with an EAPC of −0.55 (95% CI, −0.60 to −0.51). Moreover, the age-standardized prevalence rate of osteoarthritis and rheumatoid arthritis increased faster in female individuals, with EAPCs of 0.19 (95% CI, 0.13-0.25) and 0.36 (95% CI, 0.08-0.64), respectively. The sex patterns of age-standardized YLD rates resembled those of age-standardized prevalence rates.

Generally, the prevalence rates of musculoskeletal disorders increased with age. Particularly, increasing trends in both sexes peaked at age 80 to 90 years for low back pain and at age 70 to 80 years for neck pain as well as rheumatoid arthritis, whereas decreasing trends presented after the peak point with increasing age. Comparatively similar patterns were observed in YLD rates by sex across all ages (eFigure 18 and eFigure 19 in the Supplement).

### Associations Between EAPCs and Rehabilitation Needs

**Figure 3.**
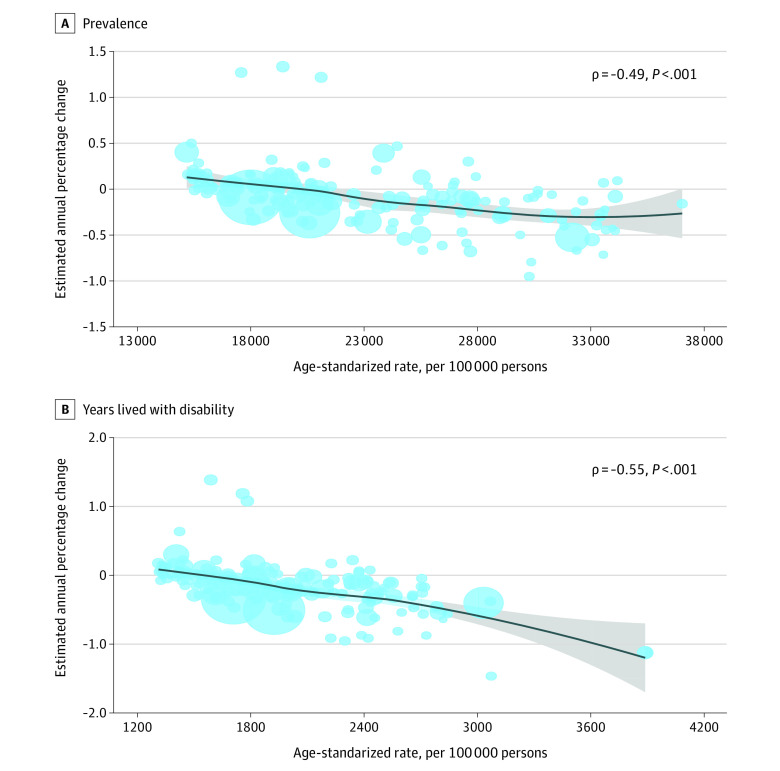
Correlations Between Estimated Annual Percentage Change and Musculoskeletal Rehabilitation Needs Age-Standardized Rates The sizes of circles represent increases in the corresponding prevalent cases or YLD counts of musculoskeletal disorders in need of rehabilitation. The ρ indices and *P* values were derived from Pearson correlation analysis. The line and shaded area represent ρ and its 95%CI.

The EAPC was negatively associated with the baseline age-standardized prevalence rate of musculoskeletal disorders in 1990 (ρ = −0.49; *P* < .001) (Figure 3A). Likewise, the EAPC was negatively associated with the baseline age-standardized YLD rate of musculoskeletal disorders in 1990 (ρ = −0.55; *P* < .001) (Figure 3B). Given the potential outliers, we reconducted the analysis after removing the outliers. The observed association between EAPCs and rehabilitation needs remained essentially the same (eFigure 20 in the Supplement). For category-specific correlations, EAPCs were significantly associated with the corresponding age-standardized rates in 1990, except for those of neck pain and rheumatoid arthritis (eFigures 21-27 in the Supplement).

### Observed vs Expected Rehabilitation Needs by UHC Effective Coverage Index

**Figure 4.**
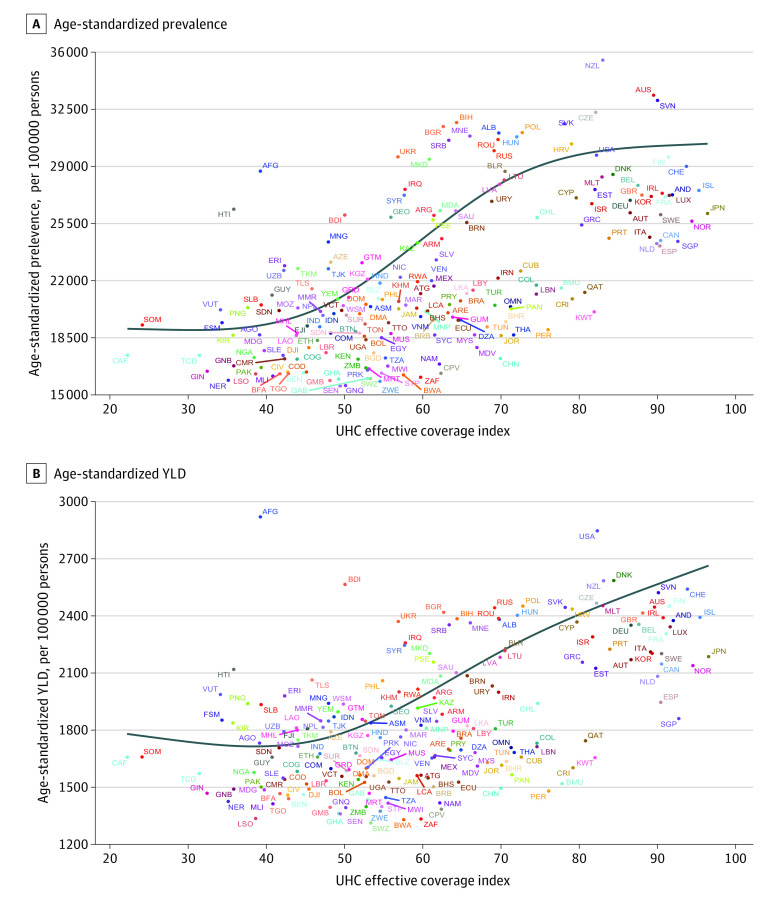
Associations Between Age-Standardized Rates and Universal Health Coverage (UHC) Effective Coverage Index in 2019 The associations were adjusted for health spending per capita, measured in 2017, and parity adjusted for 2019 purchasing power. Each point represents the observed value for each location, and the line indicates expected values. Location codes are presented in eTable 4 in the Supplement. YLD indicates years lived with disability.

Generally, a positive association was found between the age-standardized prevalence rate of musculoskeletal disorders and the UHC effective coverage index across locations after adjusting for health spending per capita (β = 749.04; SE, 288.52; *P* = .01) (Figure 4A). Observed age-standardized prevalence rates higher than expected were not only found in countries with higher UHC effective coverage indexes, such as New Zealand, Czechia, and Slovakia, but also some countries with lower UHC effective coverage indexes, including Afghanistan, Burundi, and Haiti. The association between the age-standardized YLD rate and UHC effective coverage index resembled that between the age-standardized prevalence rate and UHC effective coverage index after adjusting for health spending per capita (β = 49.70; SE, 22.89; *P* = .03) (Figure 4B). The model residuals are presented in eFigure 28 in the Supplement, which did not show substantial model inadequacy.

## Discussion

To our knowledge, this is the first study to present the secular trends in musculoskeletal rehabilitation needs over the past 3 decades at the global, regional, and national levels. From 1990 to 2019, although the global age-standardized prevalence and YLD rates of musculoskeletal disorders decreased annually with EAPCs of −0.34 (95% CI, −0.37 to −0.31) and −0.42 (95% CI, −0.51 to −0.32), respectively, substantial increases in prevalent cases and YLDs of musculoskeletal disorders were found, which was a result of population growth and aging. Importantly, nearly 1 in every 4 people needs musculoskeletal rehabilitation, which challenges the common belief that rehabilitation is needed by only a small population. Particularly, low back pain was the leading contributor to musculoskeletal rehabilitation needs worldwide. Additionally, low back pain is also the leading cause of early retirement among adults.^4^ People in Australia with low back pain experienced work incapacity, productivity decline, and early retirement, which accounted for approximately 66-fold less accumulated wealth (AUS $5038 vs $339 121) compared with their healthy counterparts working until the usual retirement age of 65 years.^4^ In addition to accumulated wealth reduction, being dependent and lack of social identity also affect people experiencing low back pain. As a result, they struggle to maintain a decent living and to take social responsibilities.^22^ Moreover, with population growth and aging, the number of people experiencing low back pain is expected to increase significantly in the following decades. Notably, in our study and supported by a previous study,^23^ the most rapid increasing trends of low back pain were in low- and middle-income countries. Therefore, the increasing disability related to low back problems contributed to productivity loss, which may perpetuate the cycle of poverty in those countries.^23^

For fractures, other injuries, and amputation, more rehabilitation resources were needed in male individuals, while for low back pain, neck pain, osteoarthritis, and rheumatoid arthritis, opposite patterns were found. This gender disparity could be explained by the combined effects of anatomic, hormonal, immunological, and traumatic factors on the development of musculoskeletal disorders.^19,24,25^ For example, male individuals need more rehabilitation resources due to fractures, as male individuals generally report a higher rate of severe traumas with increased morbidity and mortality, such as motor vehicle collisions, work-related bone overloading, and recreational injuries,^26^ although female individuals, especially those at postmenopausal stages, show a higher risk of fractures due to lower levels of bone density, declines in estrogen levels, and a higher fall rate.^27^ Similarly, higher rehabilitation needs due to amputation are found in male individuals, given that males show a higher rate of road traffic crashes^28^ and peripheral arterial disease^29^ as well as a higher risk of diabetes,^30^ which are also closely associated with individuals’ hormone levels and immune response. Notably, the age-standardized prevalence rates have increased faster in the Syrian Arab Republic, Palestine, and Pakistan, which may be an indication that traumatic events have contributed to the increasing needs of musculoskeletal rehabilitation, as those areas have been affected by conflict in recent decades. Rehabilitation needs increased with age, and rehabilitation needs due to low back pain, neck pain, and rheumatoid arthritis peaked at older ages. These results are confirmed by the GBD 2017 study findings.^1,31,32,33,34^ This has implications for health service delivery and rehabilitation resource distributions, as more attention should be paid to specific age and sex groups.

In our study, the age-standardized prevalence and YLD rates of rehabilitation needs are found to be generally higher in those countries with higher UHC effective coverage indexes. Despite this, remarkable gaps exist between observed and expected rehabilitation needs across countries. More importantly, excess rehabilitation needs reflect the limitations of health systems, and the burden of musculoskeletal disorders has exceeded health care capacity in these locations. This phenomenon existed in countries with higher and lower UHC effective coverage indexes, although countries with higher UHC effective coverage indexes tended to outperform those countries with lower UHC effective coverage indexes in terms of health care provision. One possible resolution is to identify and control risk factors, such as less exercise, overweight and obesity, an unbalanced diet, smoking, and alcohol consumption, and mitigate their impacts on the development of musculoskeletal disorders.^1,19,35^ In addition, it is essential to improve the effectiveness and efficiency of health systems.^17^ This reveals the importance of incorporating rehabilitation services into health systems and promoting rehabilitation, especially in primary health care.^10^ Furthermore, allocating sufficient funds will facilitate the provision of rehabilitation services, given that attaining UHC effective coverage indexes of 80 and greater requires $1398 per capita spending with the most effective health systems.^17^

### Limitations

This study has several limitations. First, the accuracy and precision of our research findings mainly relied on the quality and availability of data sources. For this reason, estimates for countries without available data but derived from modeled results using the GBD’s Bayesian metaregression tool, DisMod-MR 2.1, should be interpreted with caution. To ensure reliability and accuracy of results, these countries are encouraged to make additional efforts to collect data from statistics systems and sustain data quality, which may be difficult in some resource-limited areas and may warrant international cooperation. Second, rehabilitation needs were calculated based on 7 health conditions, which were included given their prevalence, severity, and the necessity of rehabilitation.^10^ Therefore, people in the early stages of health conditions might have less serve manifestations but in need of rehabilitation resources were excluded.^10^ Although this calculation might not be thorough, the aggregated results accounted for a considerable proportion of total YLDs attributable to chronic diseases and injuries in need of rehabilitation. Third, it is essential to assess the associations of risk factors with musculoskeletal disorders to provide better evidence for clinical practice and decision-making. However, these risk factors were not available from the WHO Rehabilitation Need Estimator, and thus their contributions were not assessed. Future studies may collate and assess the effects of these factors. Fourth, great variations in the effectiveness of promotion, prevention, and treatment approaches for musculoskeletal disorders among countries may affect individuals’ experience of health conditions.

## Conclusions

This study found that the number of prevalent cases and YLD counts in need of musculoskeletal rehabilitation has increased considerably during the past 30 years. High rehabilitation needs have become a global public health concern, although overall decreasing trends were found in age-standardized rates. Mitigating risk factors, strengthening rehabilitation in primary health care, and allocating sufficient funds for rehabilitation services are possible ways to meet the needs of rehabilitation.

## Notes

Supplement. **eFigure 1. **Years Lived With Disability (YLD) Estimates of Musculoskeletal Rehabilitation Needs Worldwide **eFigure 2. **Prevalence Estimates of Rehabilitation Needs Attributed to Low Back Pain Worldwide **eFigure 3. **Years Lived With Disability (YLD) Estimates of Rehabilitation Needs Attributed to Low Back Pain Worldwide **eFigure 4.** Prevalence Estimates of Rehabilitation Needs Attributed to Neck Pain Worldwide **eFigure 5.** Years Lived With Disability (YLD) Estimates of Rehabilitation Needs Attributed to Neck Pain Worldwide **eFigure 6. **Prevalence Estimates of Rehabilitation Needs Attributed to Fractures Worldwide **eFigure 7.** Years Lived With Disability (YLD) Estimates of Rehabilitation Needs Attributed to Fractures Worldwide **eFigure 8.** Prevalence Estimates of Rehabilitation Needs Attributed to Other Injuries Worldwide **eFigure 9.** Years Lived With Disability (YLD) Estimates of Rehabilitation Needs Attributed to Other Injuries Worldwide **eFigure 10.** Prevalence Estimates of Rehabilitation Needs Attributed to Osteoarthritis Worldwide **eFigure 11. **Years Lived With Disability (YLD) Estimates of Rehabilitation Needs Attributed to Osteoarthritis Worldwide **eFigure 12.** Prevalence Estimates of Rehabilitation Needs Attributed to Amputation Worldwide **eFigure 13. **Years Lived With Disability (YLD) Estimates of Rehabilitation Needs Attributed to Amputation Worldwide **eFigure 14.** Prevalence Estimates of Rehabilitation Needs Attributed to Rheumatoid Arthritis Worldwide **eFigure 15.** Years Lived With Disability (YLD) Estimates of Rehabilitation Needs Attributed to Rheumatoid Arthritis Worldwide **eFigure 16. **Age-Standardized Prevalence Rates of Musculoskeletal Rehabilitation Needs Between 1990 and 2019 **eFigure 17. **Age-Standardized Years Lived With Disability Rates of Musculoskeletal Rehabilitation Needs Between 1990 and 2019 **eFigure 18.** Prevalence Rates of Musculoskeletal Rehabilitation Needs by Sex and Age in 2019 **eFigure 19.** Years Lived With Disability Rates of Musculoskeletal Rehabilitation Needs by Sex and Age in 2019 **eFigure 20.** Correlations Between Estimated Annual Percentage Change and Musculoskeletal Rehabilitation Needs Age-Standardized Rates, Excluding Outliers **eFigure 21.** Correlations Between Estimated Annual Percentage Change and Low Back Pain Age-Standardized Rates **eFigure 22.** Correlations Between Estimated Annual Percentage Change and Neck Pain Age-Standardized Rates **eFigure 23.** Correlations Between Estimated Annual Percentage Change and Fractures Age-Standardized Rates **eFigure 24. **Correlations Between Estimated Annual Percentage Change and Other Injuries Age-Standardized Rates **eFigure 25.** Correlations Between Estimated Annual Percentage Change and Osteoarthritis Age-Standardized Rates **eFigure 26. **Correlations Between Estimated Annual Percentage Change and Amputation Age-Standardized Rates **eFigure 27.** Correlations Between Estimated Annual Percentage Change and Rheumatoid Arthritis Age-Standardized Rates **eFigure 28.** Residuals for Health Spending per Capita Adjusted Linear Regression Model **eTable 1.** Data Inputs for Injuries Incidence Modelling **eTable 2. **The List of 7 Super-Regions and 21 Regions **eTable 3.** Population Age Standard Used in the GBD 2019 Study **eTable 4.** Location Codes, Universal Health Coverage (UHC) Effective Coverage Index, and Health Spending per Capita **eTable 5.** The Age-Standardized Prevalence Rates (per 100 000 Persons) of Musculoskeletal Rehabilitation Needs in 2019 **eTable 6.** The Age-Standardized YLD Rates (per 100 000 Persons) of Musculoskeletal Rehabilitation Needs in 2019 **eTable 7. **Musculoskeletal Rehabilitation Needs and Trends Between 1990 and 2019 by Health Condition
