# Supplementary material for: Secular Trends in Musculoskeletal Rehabilitation Needs in 191 Countries and Territories From 1990 to 2019
Source: JAMA Netw Open. 2022 Jan 19;5(1):e2144198. doi: 10.1001/jamanetworkopen.2021.44198 (PMC8771302; doi:10.1001/jamanetworkopen.2021.44198)
Supplement: Supplement. — eFigure 1. Years Lived With Disability (YLD) Estimates of Musculoskeletal Rehabilitation Needs Worldwide eFigure 2. Prevalence Estimates of Rehabilitation Needs Attributed to Low Back Pain Worldwide eFigure 3. Years Lived With Disability (YLD) Estimates of Rehabilitation Needs Attributed to Low Back Pain Worldwide eFigure 4. Prevalence Estimates of Rehabilitation Needs Attributed to Neck Pain Worldwide eFigure 5. Years Lived With Disability (YLD) Estimates of Rehabilitation Needs Attributed to Neck Pain Worldwide eFigure 6. Prevalence Estimates of Rehabilitation Needs Attributed to Fractures Worldwide eFigure 7. Years Lived With Disability (YLD) Estimates of Rehabilitation Needs Attributed to Fractures Worldwide eFigure 8. Prevalence Estimates of Rehabilitation Needs Attributed to Other Injuries Worldwide eFigure 9. Years Lived With Disability (YLD) Estimates of Rehabilitation Needs Attributed to Other Injuries Worldwide eFigure 10. Prevalence Estimates of Rehabilitation Needs Attributed to Osteoarthritis Worldwide eFigure 11. Years Lived With Disability (YLD) Estimates of Rehabilitation Needs Attributed to Osteoarthritis Worldwide eFigure 12. Prevalence Estimates of Rehabilitation Needs Attributed to Amputation Worldwide eFigure 13. Years Lived With Disability (YLD) Estimates of Rehabilitation Needs Attributed to Amputation Worldwide eFigure 14. Prevalence Estimates of Rehabilitation Needs Attributed to Rheumatoid Arthritis Worldwide eFigure 15. Years Lived With Disability (YLD) Estimates of Rehabilitation Needs Attributed to Rheumatoid Arthritis Worldwide eFigure 16. Age-Standardized Prevalence Rates of Musculoskeletal Rehabilitation Needs Between 1990 and 2019 eFigure 17. Age-Standardized Years Lived With Disability Rates of Musculoskeletal Rehabilitation Needs Between 1990 and 2019 eFigure 18. Prevalence Rates of Musculoskeletal Rehabilitation Needs by Sex and Age in 2019 eFigure 19. Years Lived With Disability Rates of Musculoskeletal Rehabilitation Needs by Se [file jamanetwopen-e2144198-s001.pdf]

Supplemental Online Content

Chen N, Fong DYT, Wong JYH. Secular trends in musculoskeletal rehabilitation needs in 191 countries and territories from 1990 to 2019. *JAMA Netw Open.* 2022;5(1):e2144198. doi:10.1001/jamanetworkopen.2021.44198

eFigure 1. Years Lived With Disability (YLD) Estimates of Musculoskeletal Rehabilitation Needs Worldwide
eFigure 2. Prevalence Estimates of Rehabilitation Needs Attributed to Low Back Pain Worldwide
eFigure 3. Years Lived With Disability (YLD) Estimates of Rehabilitation Needs Attributed to Low Back Pain Worldwide
eFigure 4. Prevalence Estimates of Rehabilitation Needs Attributed to Neck Pain Worldwide
eFigure 5. Years Lived With Disability (YLD) Estimates of Rehabilitation Needs Attributed to Neck Pain Worldwide
eFigure 6. Prevalence Estimates of Rehabilitation Needs Attributed to Fractures Worldwide
eFigure 7. Years Lived With Disability (YLD) Estimates of Rehabilitation Needs Attributed to Fractures Worldwide
eFigure 8. Prevalence Estimates of Rehabilitation Needs Attributed to Other Injuries Worldwide
eFigure 9. Years Lived With Disability (YLD) Estimates of Rehabilitation Needs Attributed to Other Injuries Worldwide
eFigure 10. Prevalence Estimates of Rehabilitation Needs Attributed to Osteoarthritis Worldwide
eFigure 11. Years Lived With Disability (YLD) Estimates of Rehabilitation Needs Attributed to Osteoarthritis Worldwide
eFigure 12. Prevalence Estimates of Rehabilitation Needs Attributed to Amputation Worldwide
eFigure 13. Years Lived With Disability (YLD) Estimates of Rehabilitation Needs Attributed to Amputation Worldwide
eFigure 14. Prevalence Estimates of Rehabilitation Needs Attributed to Rheumatoid Arthritis Worldwide
eFigure 15. Years Lived With Disability (YLD) Estimates of Rehabilitation Needs Attributed to Rheumatoid Arthritis Worldwide
eFigure 16. Age-Standardized Prevalence Rates of Musculoskeletal Rehabilitation Needs Between 1990 and 2019
eFigure 17. Age-Standardized Years Lived With Disability Rates of Musculoskeletal Rehabilitation Needs Between 1990 and 2019
eFigure 18. Prevalence Rates of Musculoskeletal Rehabilitation Needs by Sex and Age in 2019

© 2022 Chen N et al. *JAMA Network Open.*

eFigure 19. Years Lived With Disability Rates of Musculoskeletal Rehabilitation Needs by Sex and Age in 2019
eFigure 20. Correlations Between Estimated Annual Percentage Change and Musculoskeletal Rehabilitation Needs Age-Standardized Rates, Excluding Outliers
eFigure 21. Correlations Between Estimated Annual Percentage Change and Low Back Pain Age-Standardized Rates
eFigure 22. Correlations Between Estimated Annual Percentage Change and Neck Pain Age-Standardized Rates
eFigure 23. Correlations Between Estimated Annual Percentage Change and Fractures Age-Standardized Rates
eFigure 24. Correlations Between Estimated Annual Percentage Change and Other Injuries Age-Standardized Rates
eFigure 25. Correlations Between Estimated Annual Percentage Change and Osteoarthritis Age-Standardized Rates
eFigure 26. Correlations Between Estimated Annual Percentage Change and Amputation Age-Standardized Rates
eFigure 27. Correlations Between Estimated Annual Percentage Change and Rheumatoid Arthritis Age-Standardized Rates
eFigure 28. Residuals for Health Spending per Capita Adjusted Linear Regression Model
eTable 1. Data Inputs for Injuries Incidence Modelling
eTable 2. The List of 7 Super-Regions and 21 Regions
eTable 3. Population Age Standard Used in the GBD 2019 Study
eTable 4. Location Codes, Universal Health Coverage (UHC) Effective Coverage Index, and Health Spending per Capita
eTable 5. The Age-Standardized Prevalence Rates (per 100 000 Persons) of Musculoskeletal Rehabilitation Needs in 2019
eTable 6. The Age-Standardized YLDs Rates (per 100 000 Persons) of Musculoskeletal Rehabilitation Needs in 2019
eTable 7. Musculoskeletal Rehabilitation Needs and Trends Between 1990 and 2019 by Health Condition

This supplemental material has been provided by the authors to give readers additional information about their work.

eFigure 1. Years Lived With Disability (YLD) Estimates of Musculoskeletal Rehabilitation Needs Worldwide

A

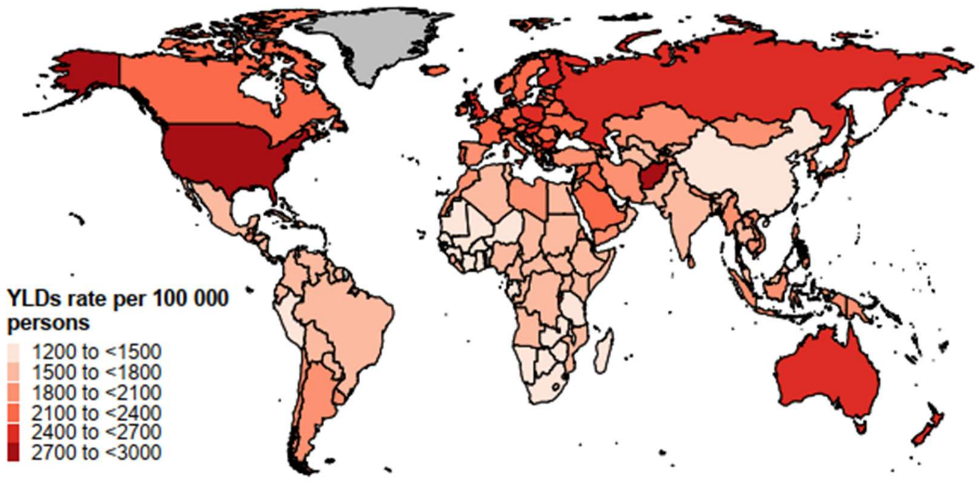

B

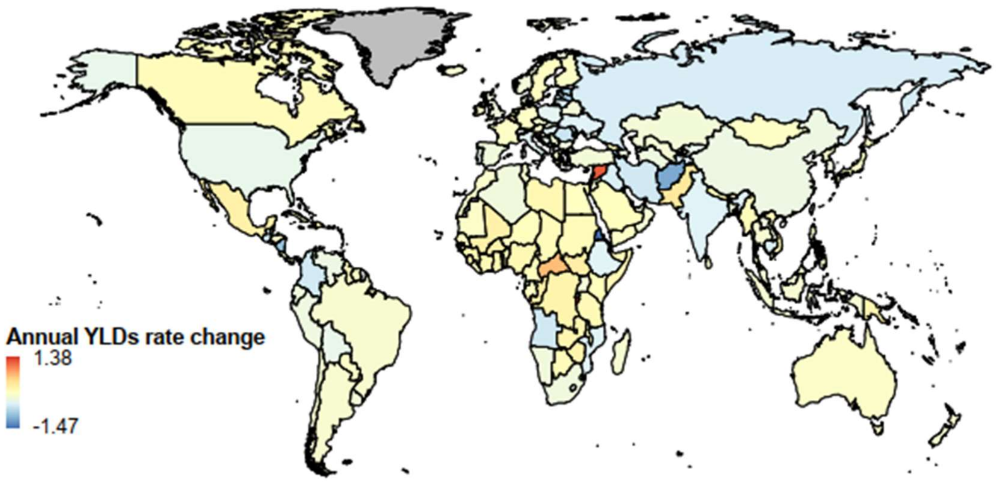

Age-standardized YLDs rate (per 100 000 persons) in 2019 (A), and the annual YLDs rate change, 1990-2019 (B).

© 2022 Chen N et al. *JAMA Network Open.*

eFigure 2. Prevalence Estimates of Rehabilitation Needs Attributed to Low Back Pain Worldwide

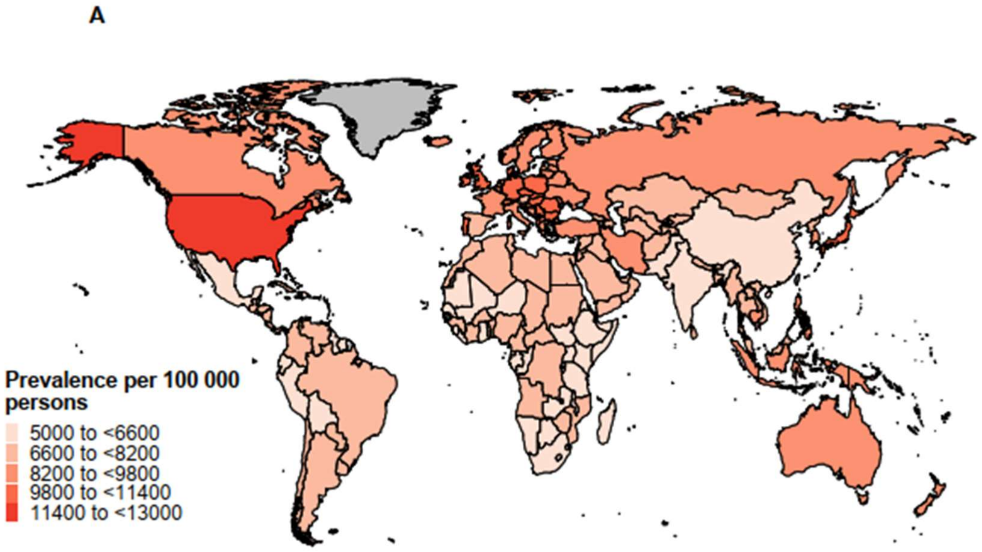

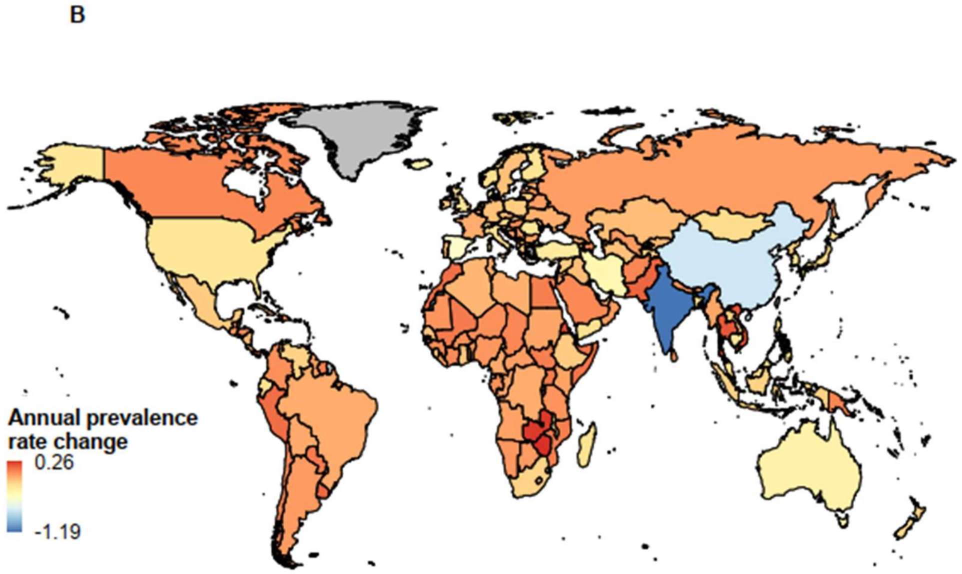

Age-standardized prevalence rate (per 100 000 persons) in 2019 (A), and the annual prevalence rate change, 1990-2019 (B).

© 2022 Chen N et al. *JAMA Network Open.*

eFigure 3. Years Lived With Disability (YLD) Estimates of Rehabilitation Needs Attributed to Low Back Pain Worldwide

A

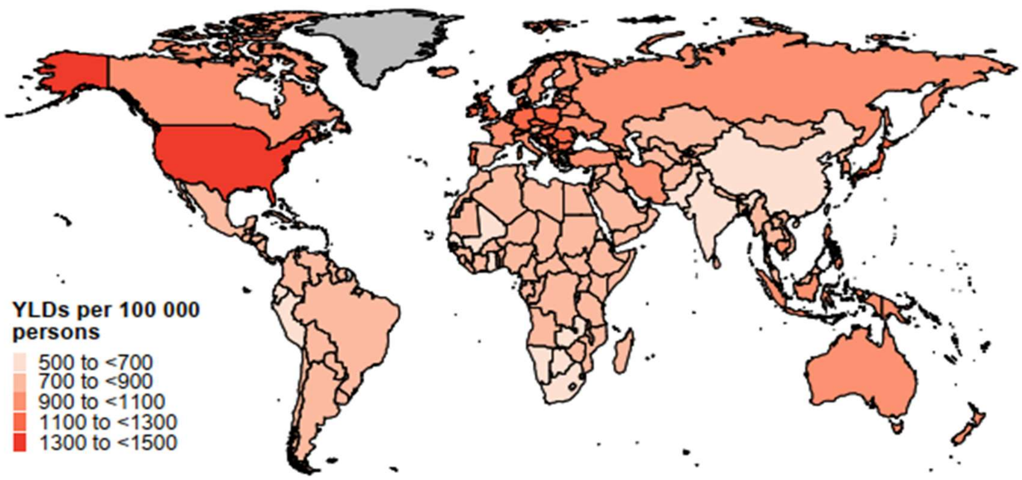

B

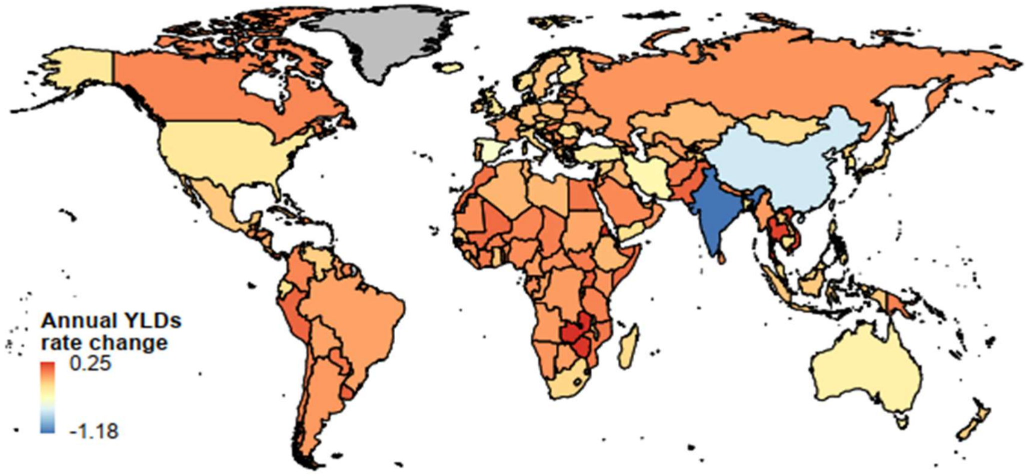

Age-standardized YLDs rate (per 100 000 persons) in 2019 (A), and the annual YLDs rate change, 1990-2019 (B).

© 2022 Chen N et al. *JAMA Network Open.*

eFigure 4. Prevalence Estimates of Rehabilitation Needs Attributed to Neck Pain Worldwide

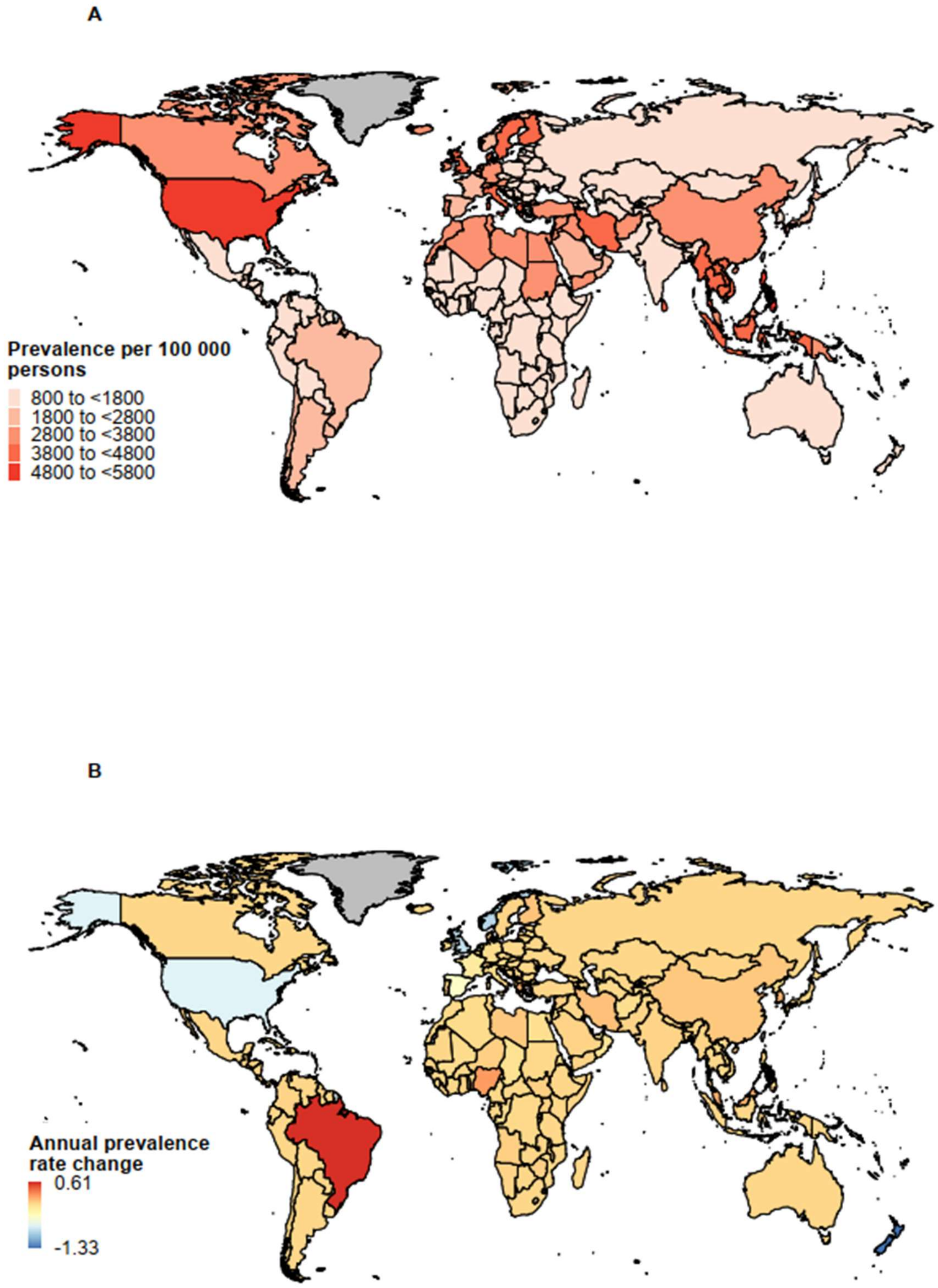

Age-standardized prevalence rate (per 100 000 persons) in 2019 (A), and the annual prevalence rate change, 1990-2019 (B).

© 2022 Chen N et al. *JAMA Network Open.*

eFigure 5. Years Lived With Disability (YLD) Estimates of Rehabilitation Needs Attributed to Neck Pain Worldwide

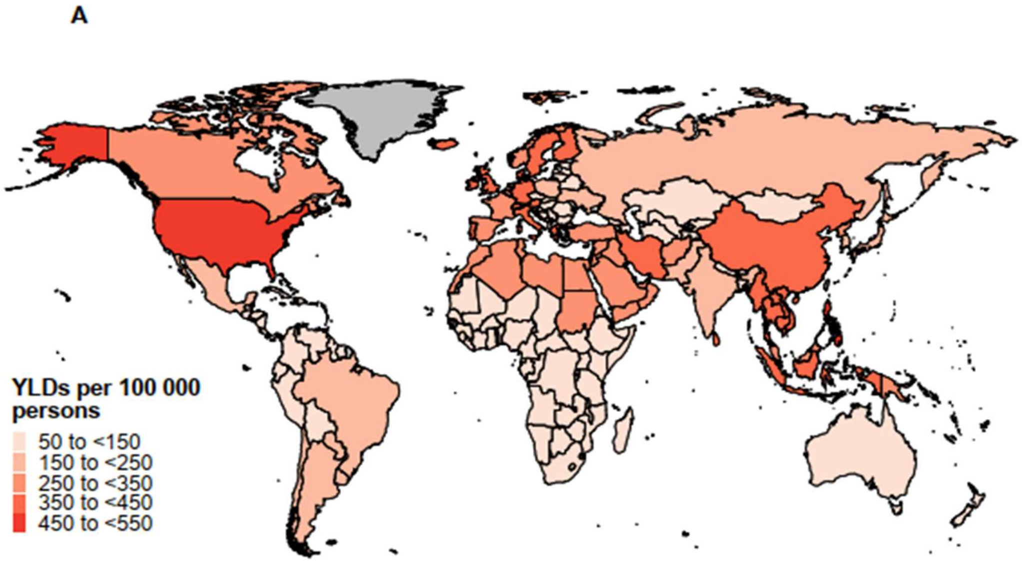

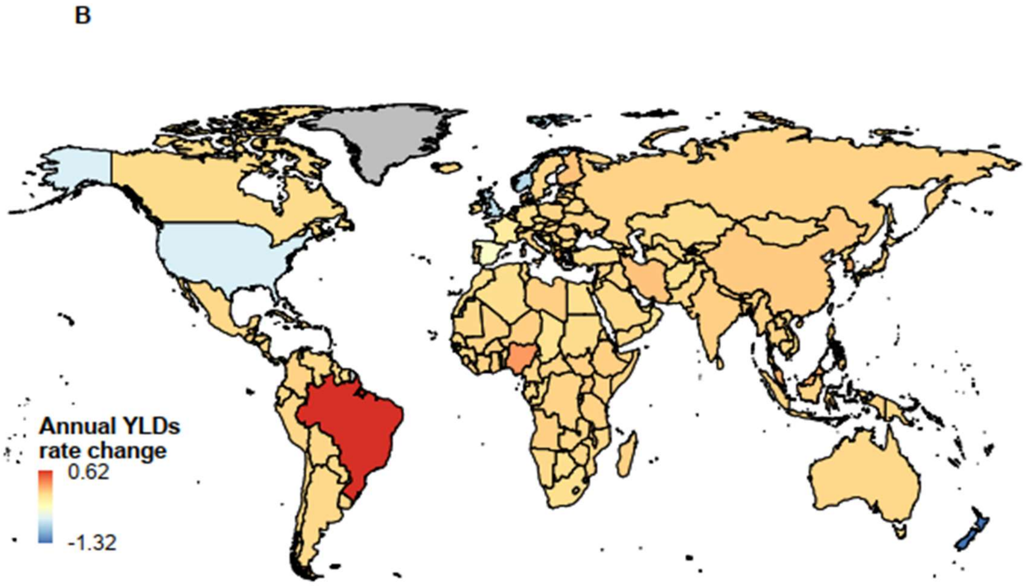

Age-standardized YLDs rate (per 100 000 persons) in 2019 (A), and the annual YLDs rate change, 1990-2019 (B).

© 2022 Chen N et al. *JAMA Network Open.*

eFigure 6. Prevalence Estimates of Rehabilitation Needs Attributed to Fractures Worldwide

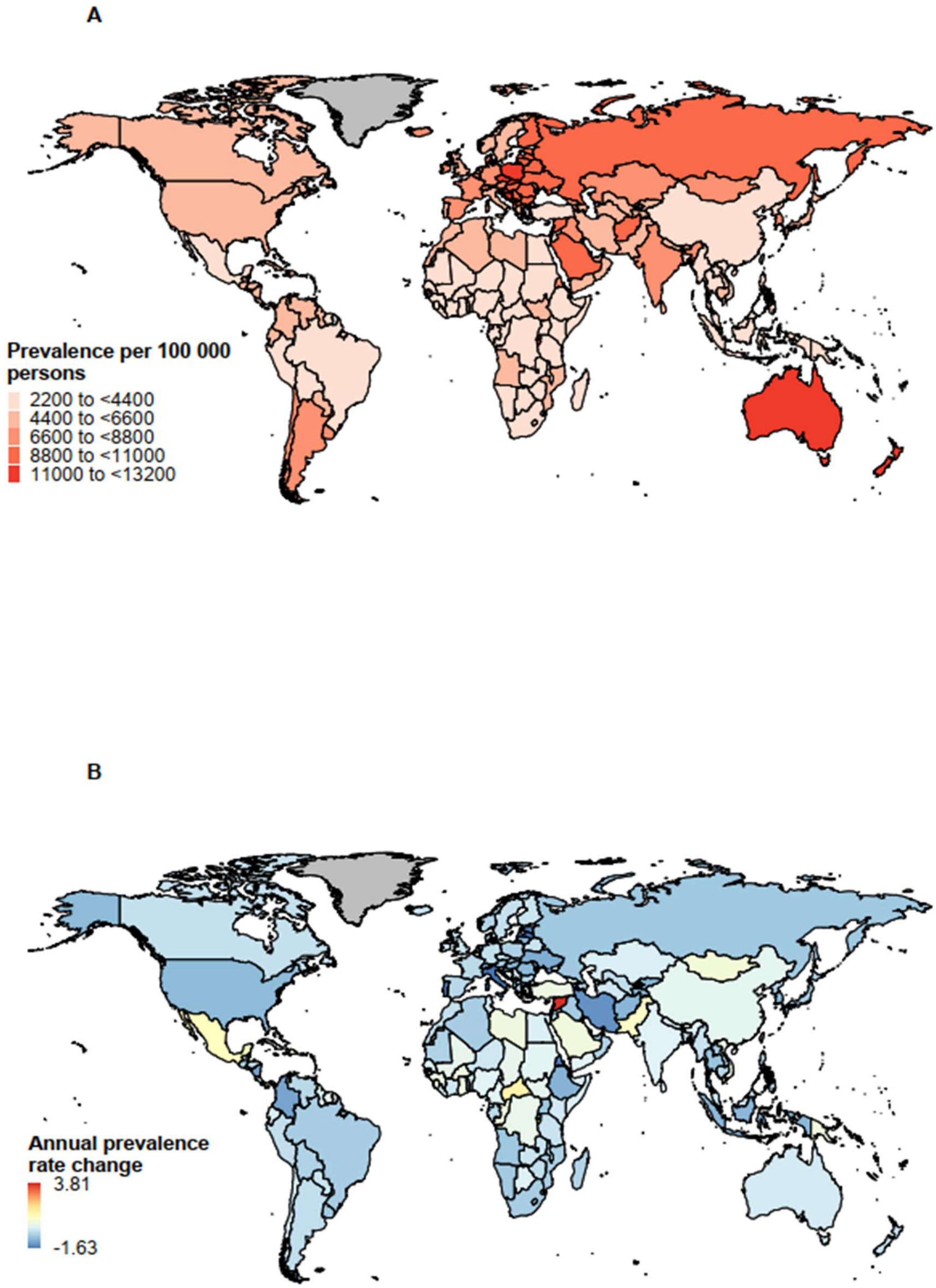

Age-standardized prevalence rate (per 100 000 persons) in 2019 (A), and the annual prevalence rate change, 1990-2019 (B).

© 2022 Chen N et al. *JAMA Network Open.*

eFigure 7. Years Lived With Disability (YLD) Estimates of Rehabilitation Needs Attributed to Fractures Worldwide

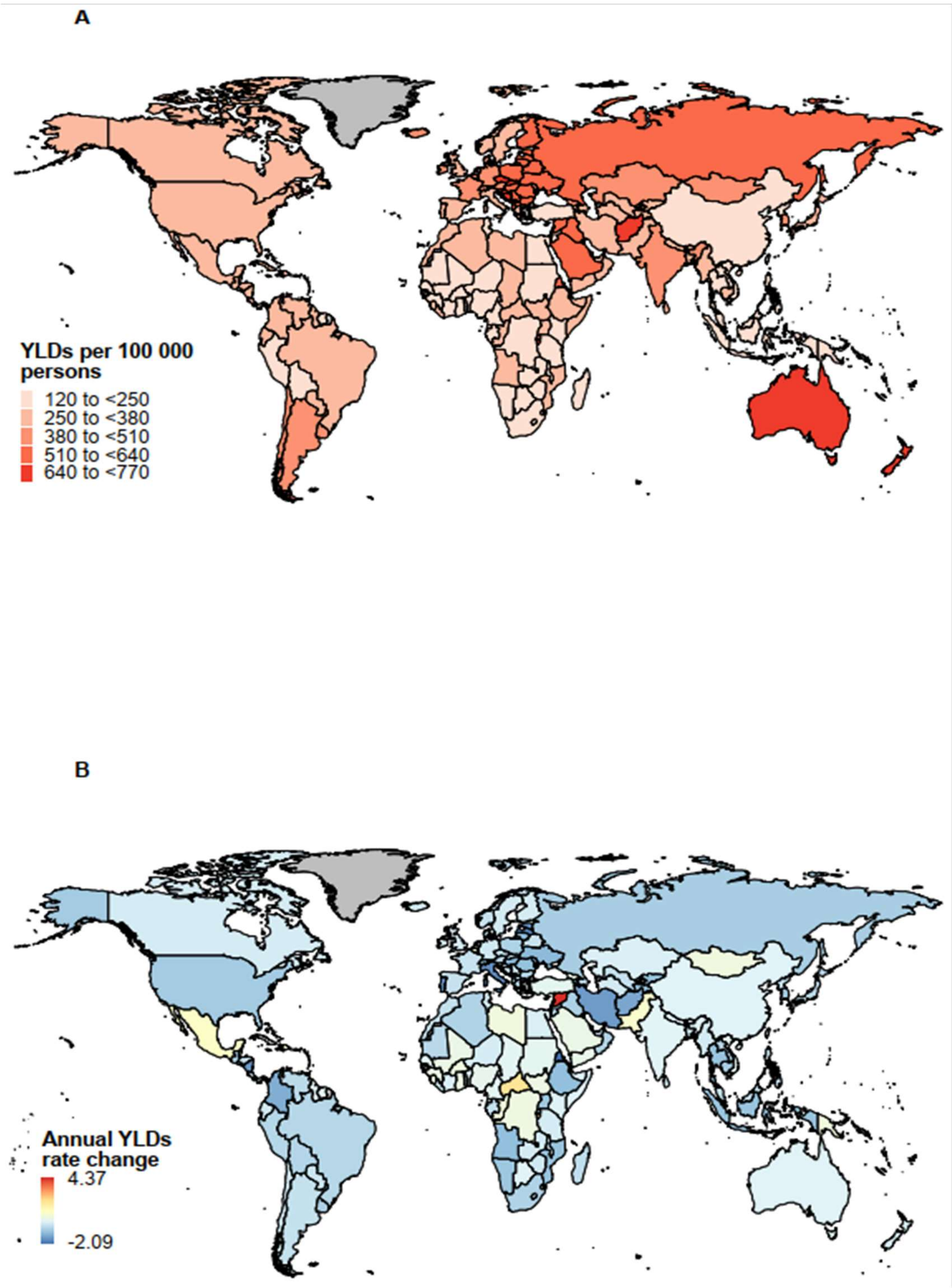

Age-standardized YLDs rate (per 100 000 persons) in 2019 (A), and the annual YLDs rate change, 1990-2019 (B).

© 2022 Chen N et al. *JAMA Network Open.*

eFigure 8. Prevalence Estimates of Rehabilitation Needs Attributed to Other Injuries Worldwide

A

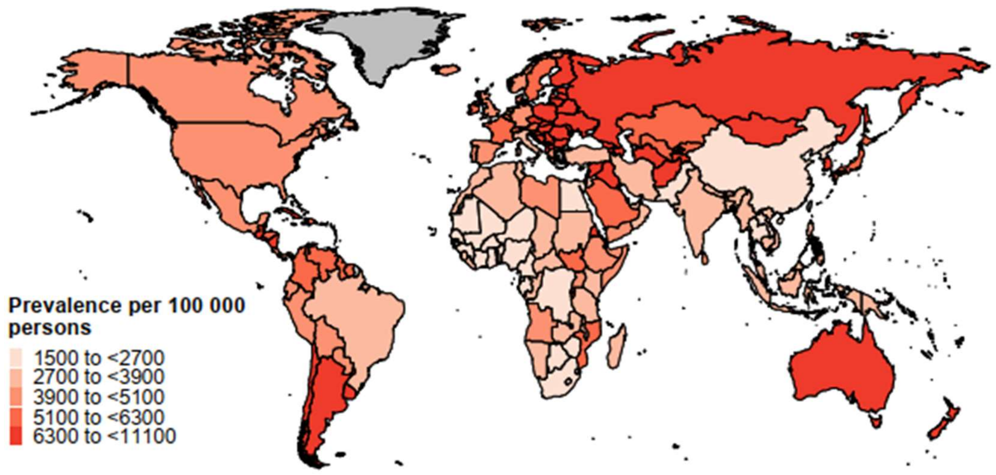

B

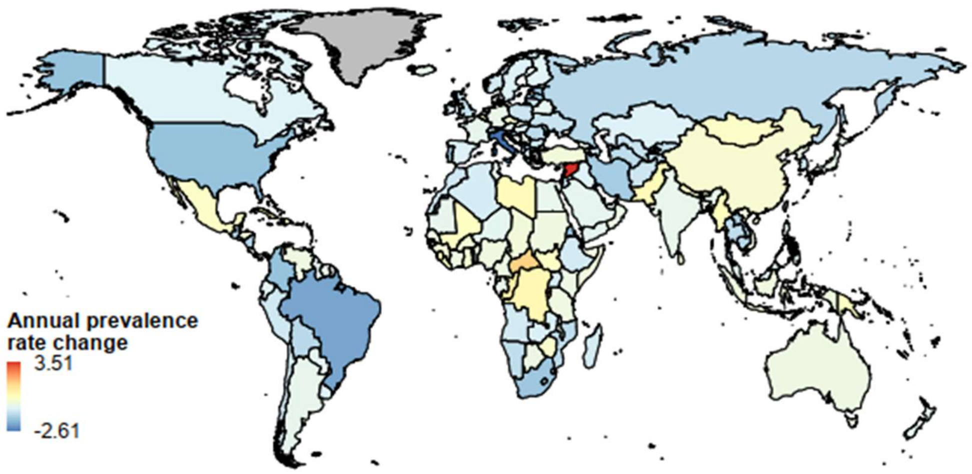

Age-standardized prevalence rate (per 100 000 persons) in 2019 (A), and the annual prevalence rate change, 1990-2019 (B).

© 2022 Chen N et al. *JAMA Network Open.*

eFigure 9. Years Lived With Disability (YLD) Estimates of Rehabilitation Needs Attributed to Other Injuries Worldwide

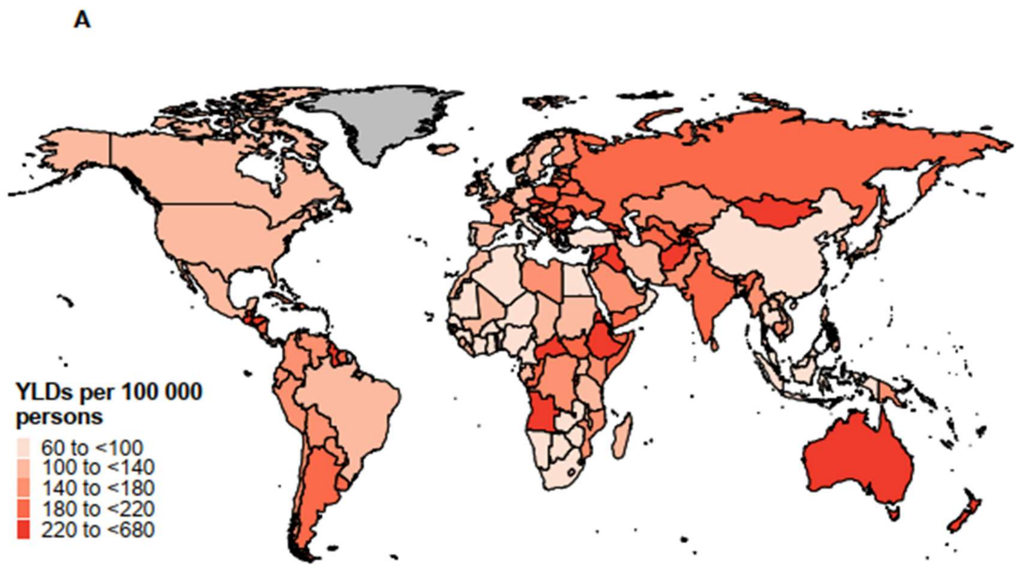

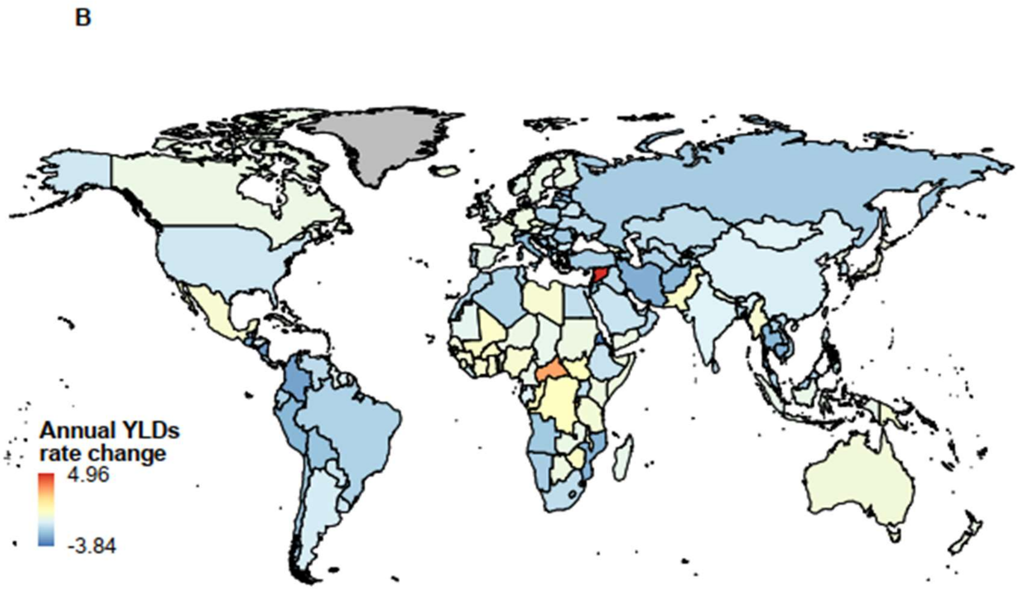

Age-standardized YLDs rate (per 100 000 persons) in 2019 (A), and the annual YLDs rate change, 1990-2019 (B).

© 2022 Chen N et al. *JAMA Network Open.*

eFigure 10. Prevalence Estimates of Rehabilitation Needs Attributed to Osteoarthritis Worldwide

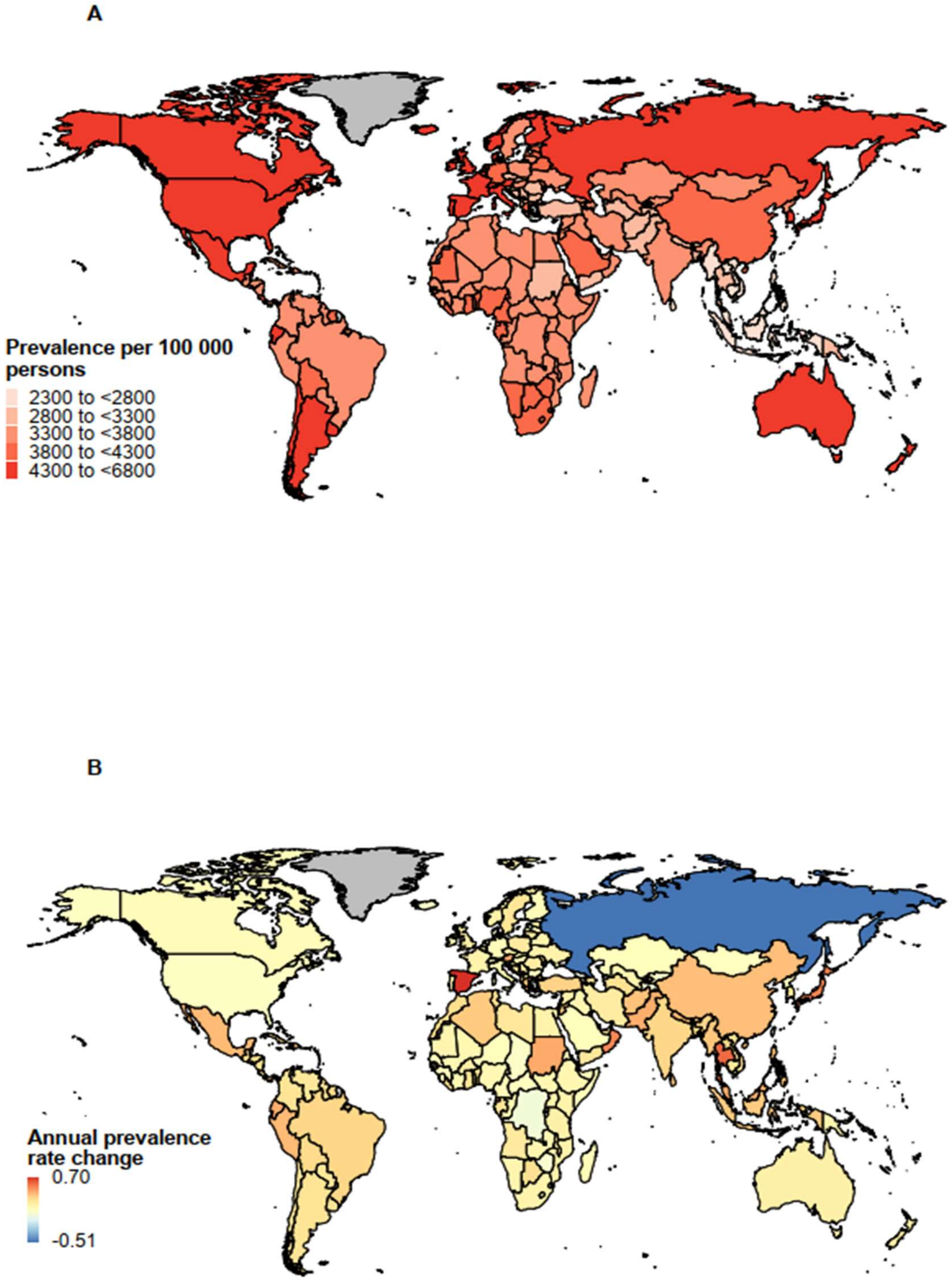

Age-standardized prevalence rate (per 100 000 persons) in 2019 (A), and the annual prevalence rate change, 1990-2019 (B).

© 2022 Chen N et al. *JAMA Network Open.*

eFigure 11. Years Lived With Disability (YLD) Estimates of Rehabilitation Needs Attributed to Osteoarthritis Worldwide

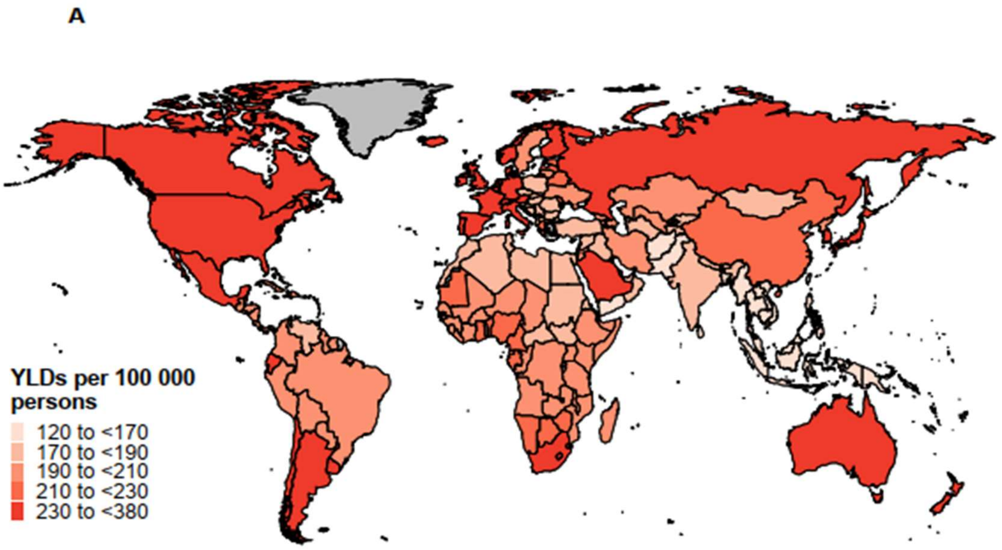

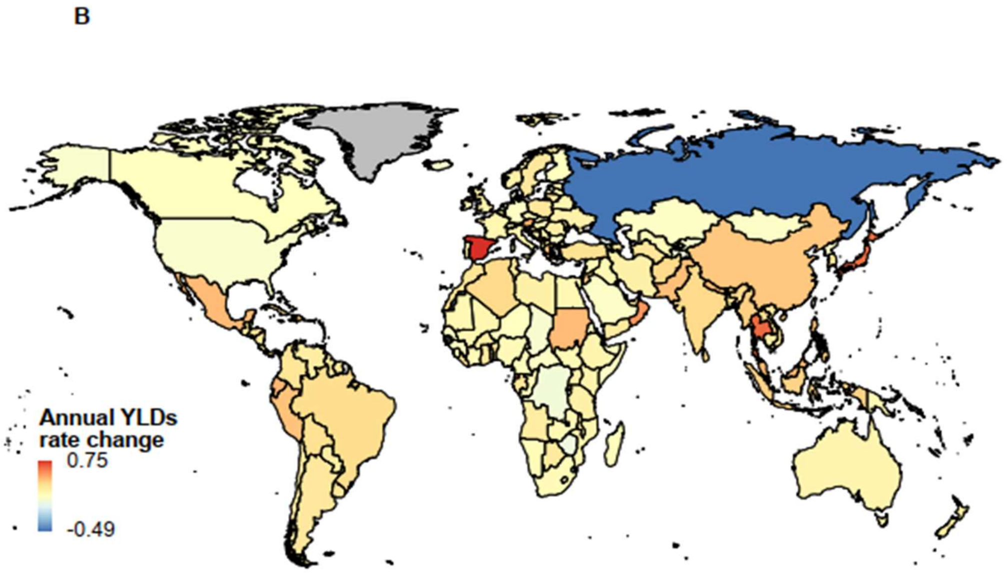

Age-standardized YLDs rate (per 100 000 persons) in 2019 (A), and the annual YLDs rate change, 1990-2019 (B).

© 2022 Chen N et al. *JAMA Network Open.*

eFigure 12. Prevalence Estimates of Rehabilitation Needs Attributed to Amputation Worldwide

A

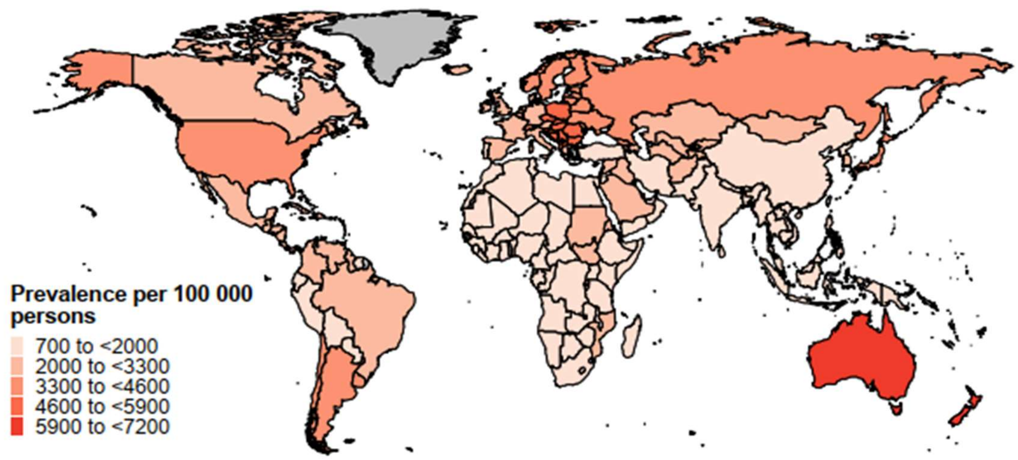

B

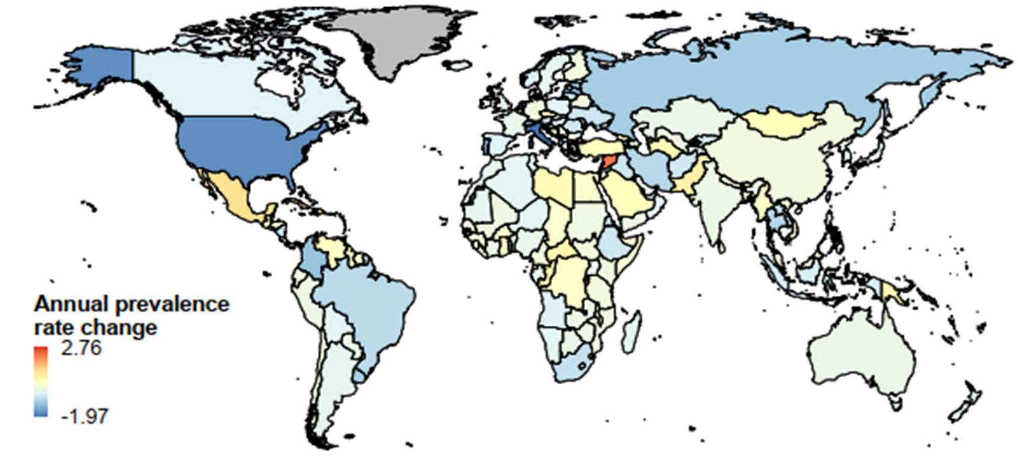

Age-standardized prevalence rate (per 100 000 persons) in 2019 (A), and the annual prevalence rate change, 1990-2019 (B).

© 2022 Chen N et al. *JAMA Network Open.*

eFigure 13. Years Lived With Disability (YLD) Estimates of Rehabilitation Needs Attributed to Amputation Worldwide

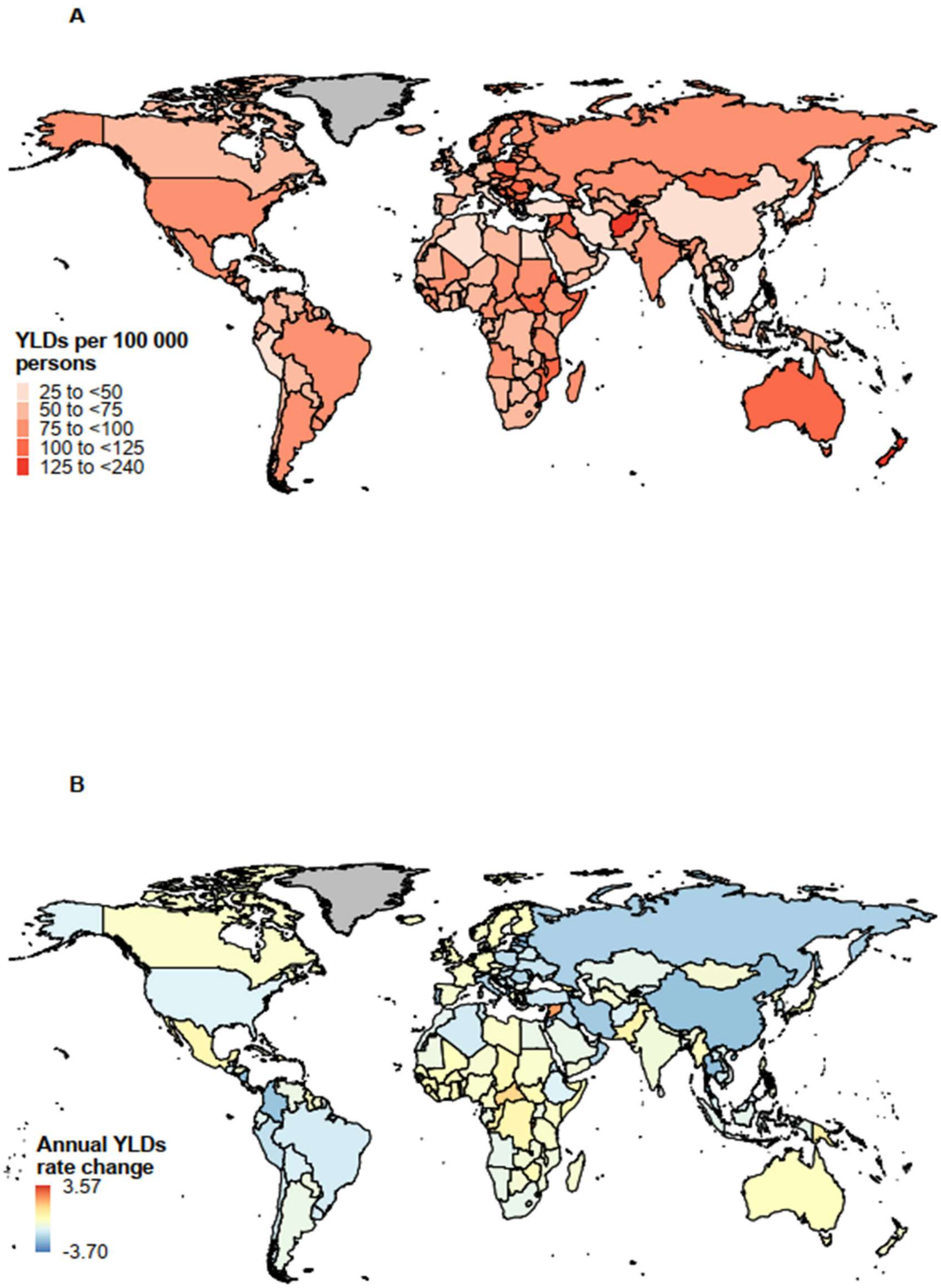

Age-standardized YLDs rate (per 100 000 persons) in 2019 (A), and the annual YLDs rate change, 1990-2019 (B).

© 2022 Chen N et al. *JAMA Network Open.*

eFigure 14. Prevalence Estimates of Rehabilitation Needs Attributed to Rheumatoid Arthritis Worldwide

A

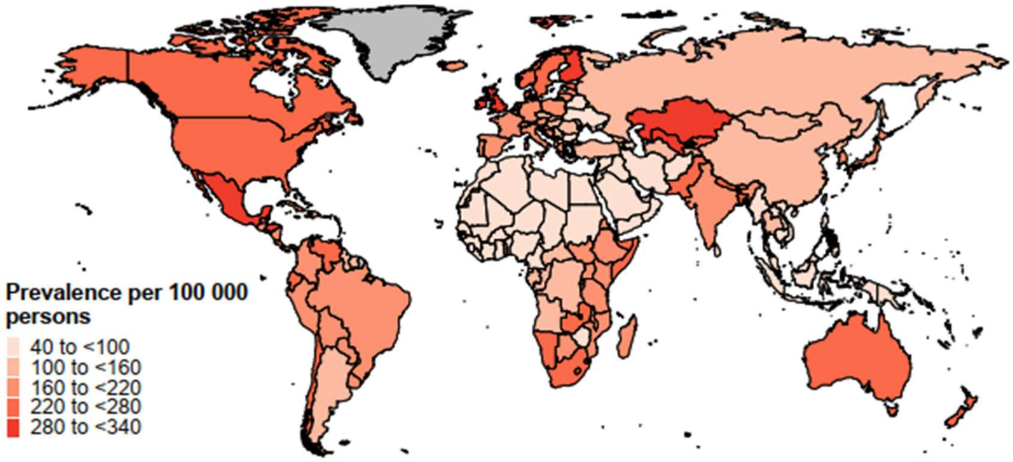

B

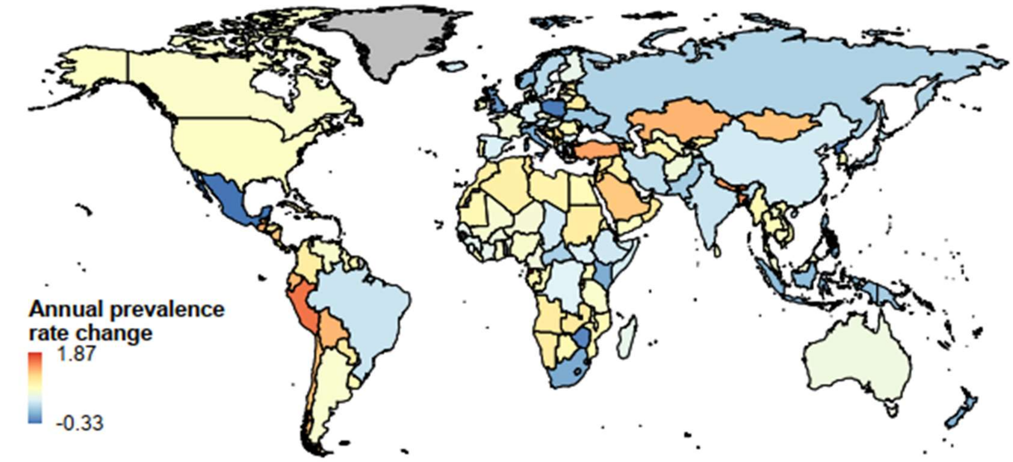

Age-standardized prevalence rate (per 100 000 persons) in 2019 (A), and the annual prevalence rate change, 1990-2019 (B).

© 2022 Chen N et al. *JAMA Network Open.*

eFigure 15. Years Lived With Disability (YLD) Estimates of Rehabilitation Needs Attributed to Rheumatoid Arthritis Worldwide

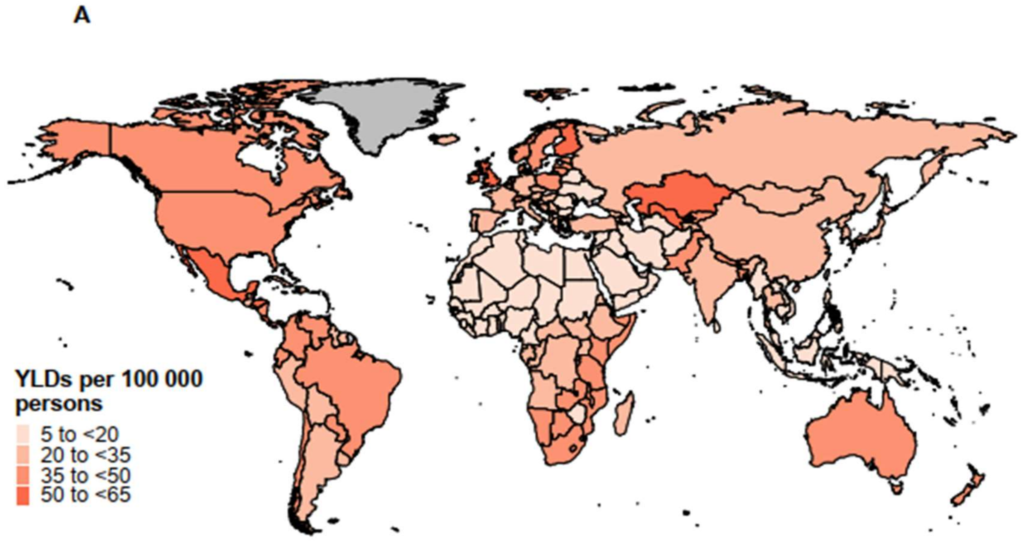

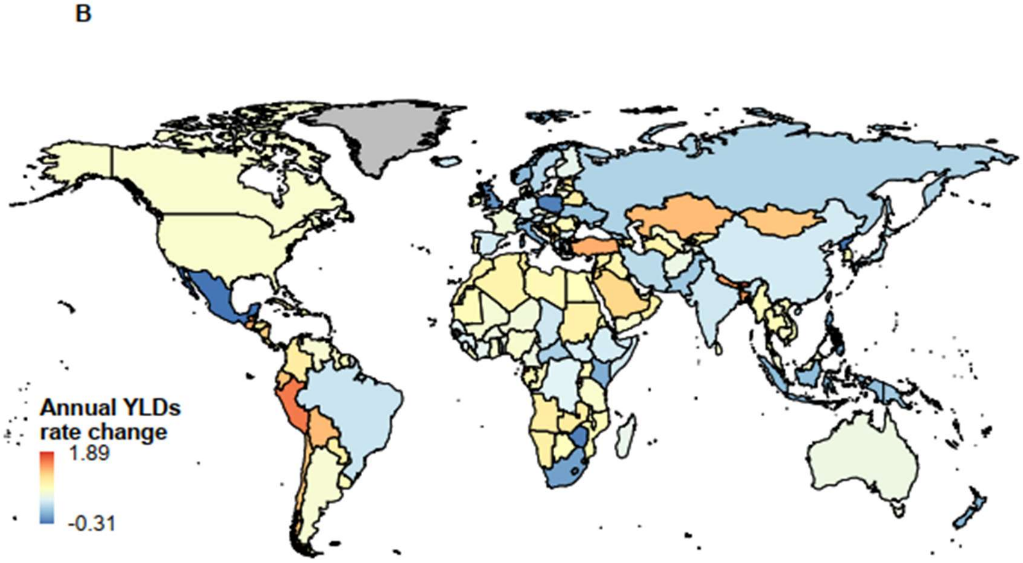

Age-standardized YLDs rate (per 100 000 persons) in 2019 (A), and the annual YLDs rate change, 1990-2019 (B).

© 2022 Chen N et al. *JAMA Network Open.*

eFigure 16. Age-Standardized Prevalence Rates of Musculoskeletal Rehabilitation Needs Between 1990 and 2019

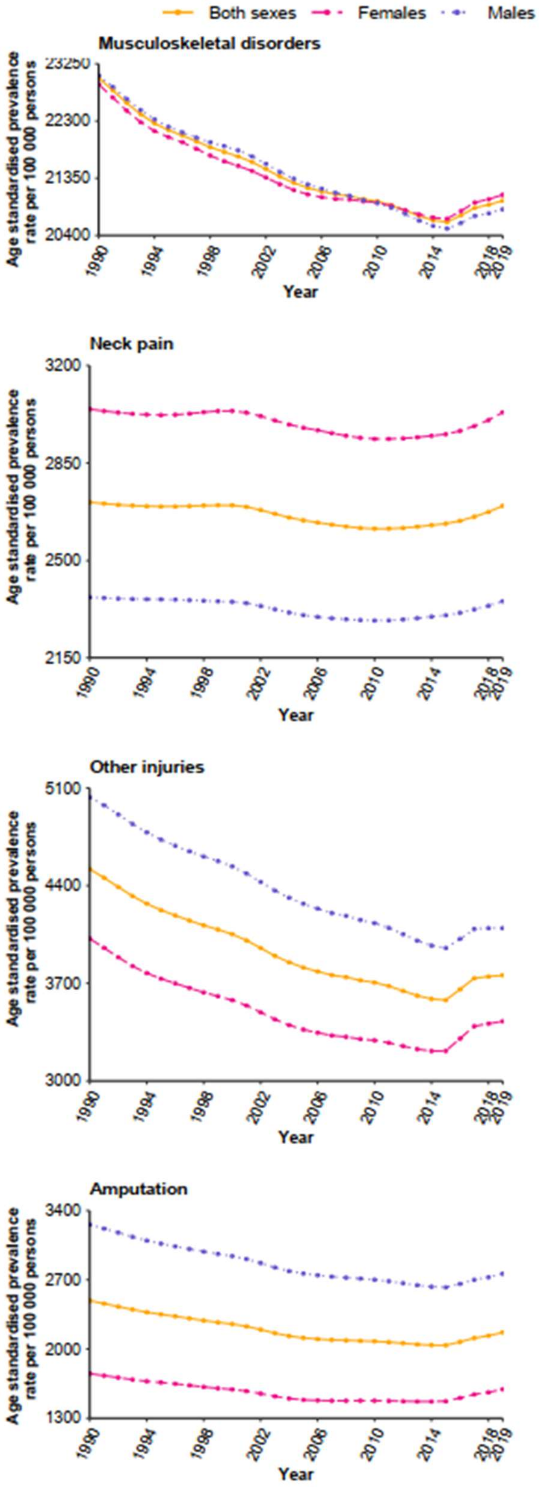

© 2022 Chen N et al. *JAMA Network Open.*

eFigure 17. Age-Standardized Years Lived With Disability Rates of Musculoskeletal Rehabilitation Needs Between 1990 and 2019

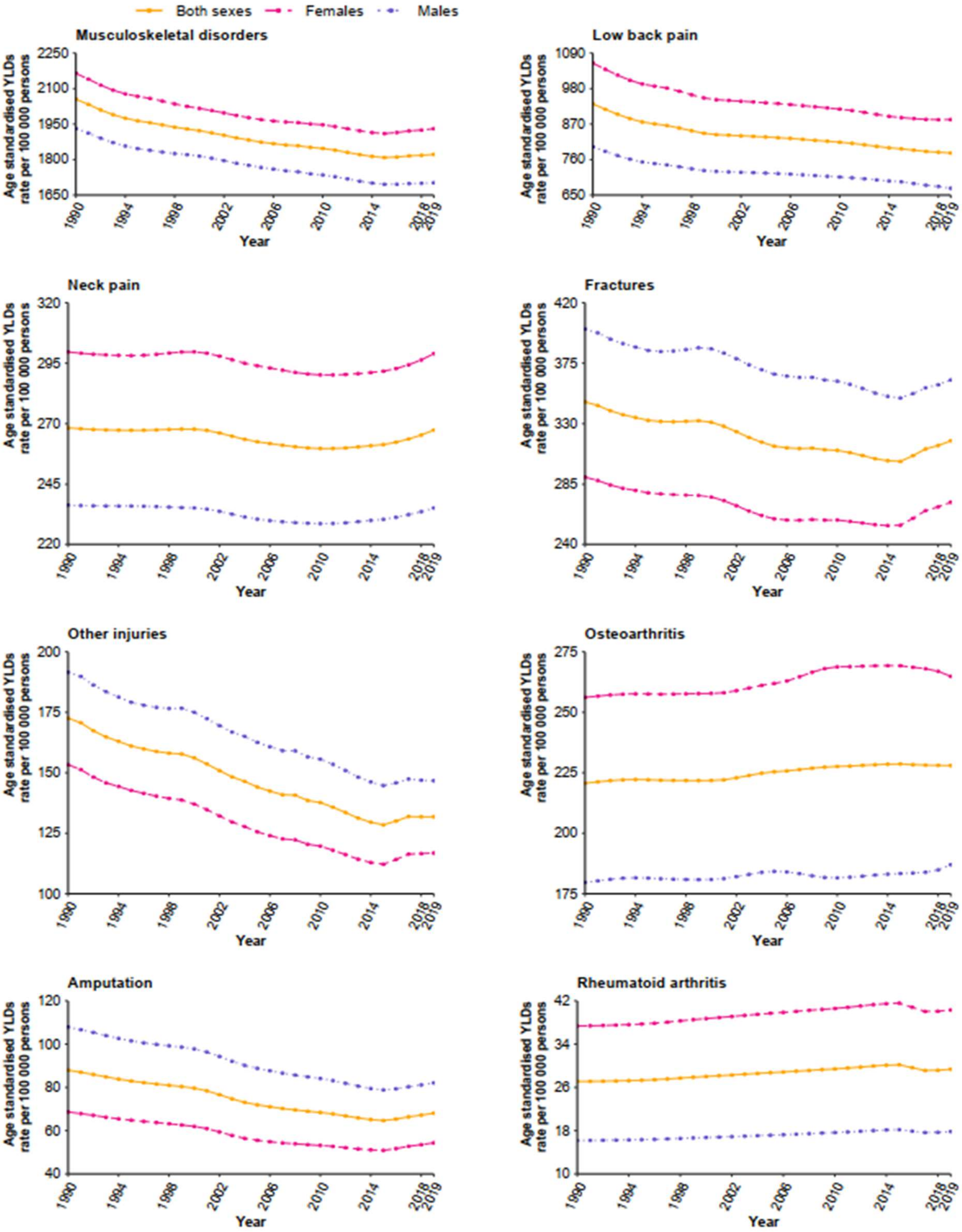

© 2022 Chen N et al. *JAMA Network Open.*

eFigure 18. Prevalence Rates of Musculoskeletal Rehabilitation Needs by Sex and Age in 2019

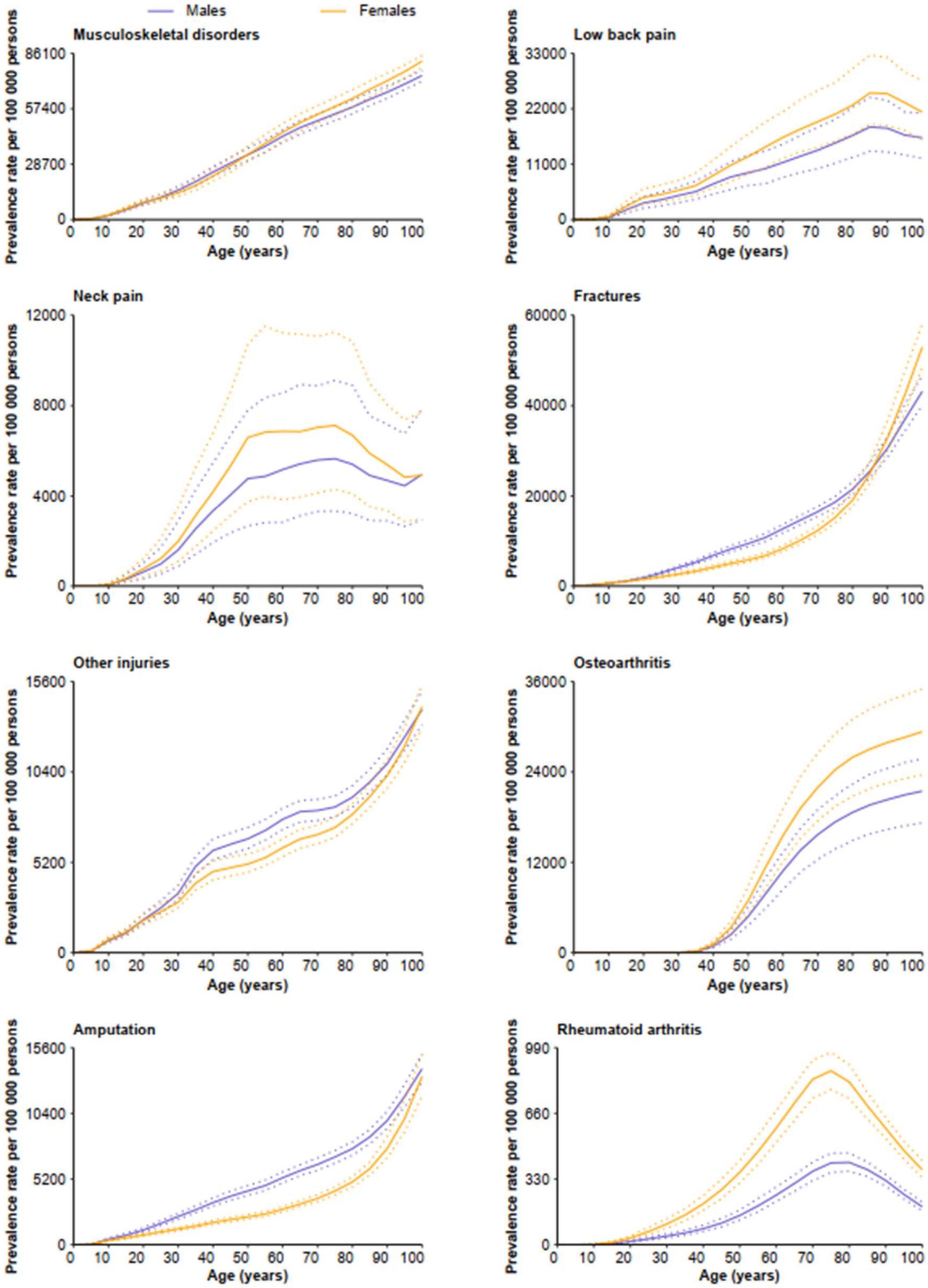

Dashed lines show 95% uncertainty interval.

© 2022 Chen N et al. *JAMA Network Open.*

eFigure 19. Years Lived With Disability Rates of Musculoskeletal Rehabilitation Needs by Sex and Age in 2019

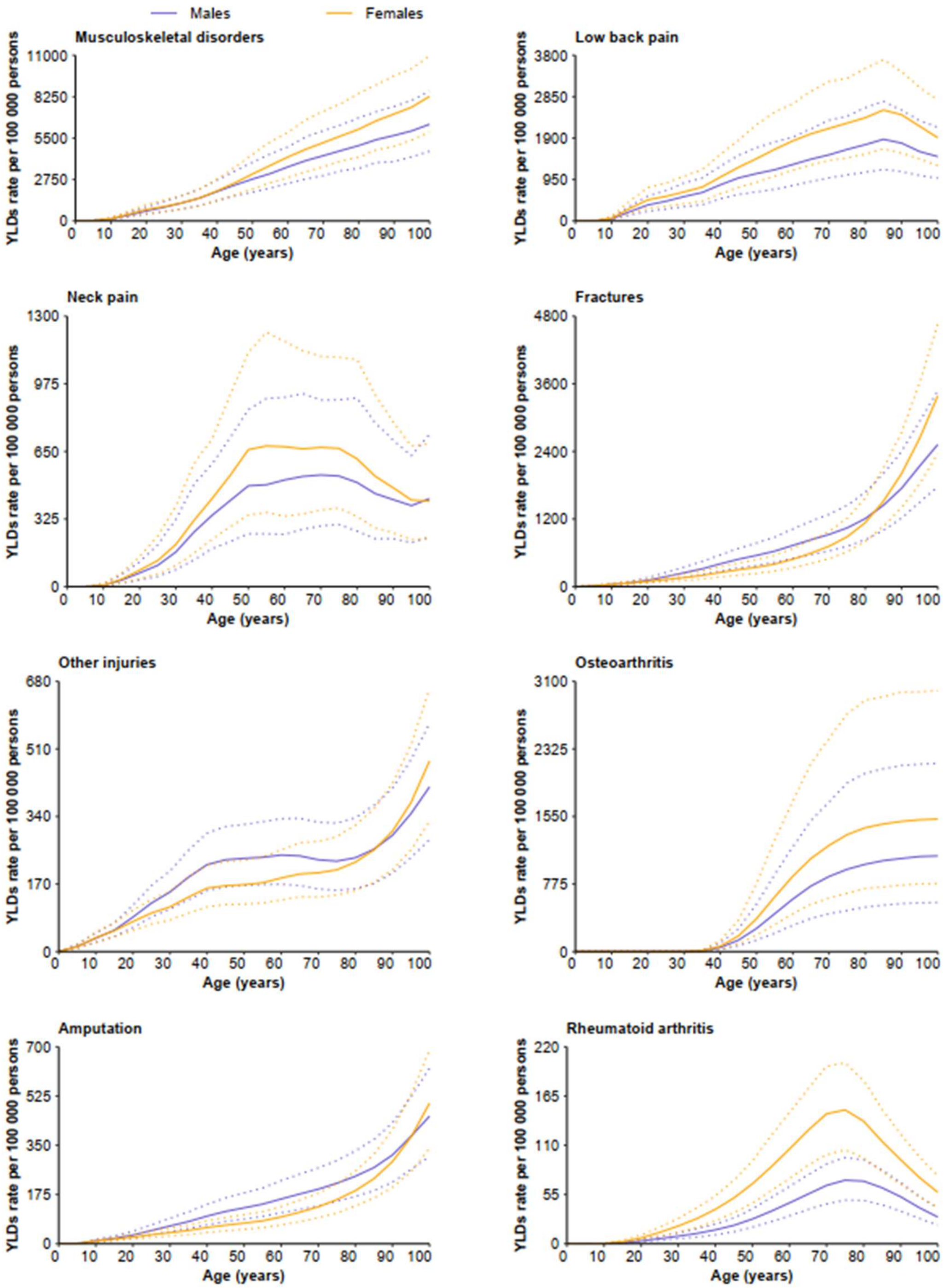

Dashed lines show 95% uncertainty interval.

© 2022 Chen N et al. *JAMA Network Open.*

eFigure 20. Correlations Between Estimated Annual Percentage Change and Musculoskeletal Rehabilitation Needs Age-Standardized Rates, Excluding Outliers

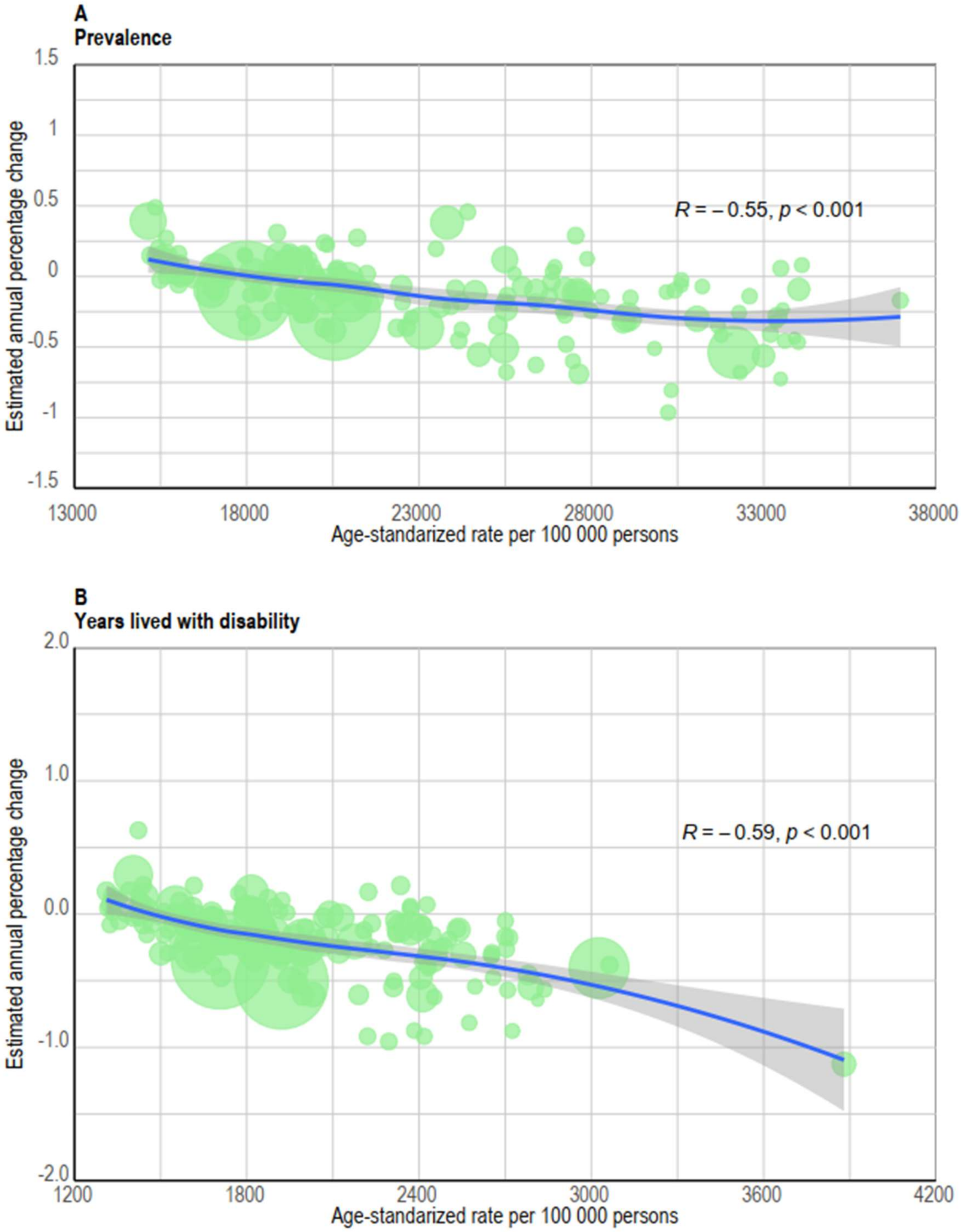

Correlation with age-standardized prevalence rate (A), and age-standardized years lived with disability (YLDs) rate (B). The size of green circle represents increases in the corresponding prevalent cases or YLDs counts of musculoskeletal disorders in need of rehabilitation. The ρ indices and *P* values were derived from Pearson correlation analysis. Blue line and shaded area represent ρ and its 95%CI.

© 2022 Chen N et al. *JAMA Network Open.*

eFigure 21. Correlations Between Estimated Annual Percentage Change and Low Back Pain Age-Standardized Rates

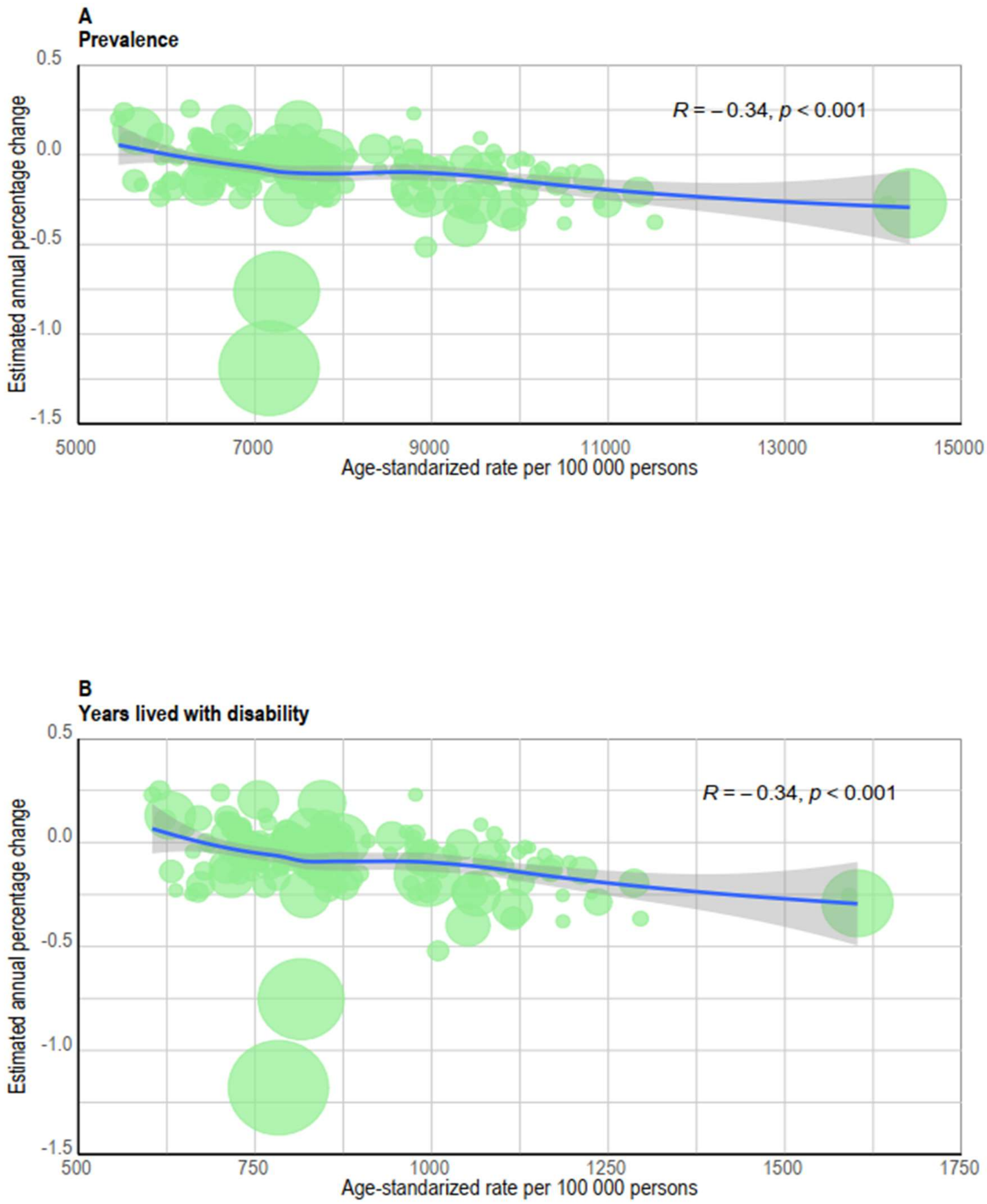

Correlation with age-standardized prevalence rate (A), and age-standardized years lived with disability (YLDs) rate (B). The size of green circle represents increases in the corresponding prevalent cases or YLDs counts of low back pain in need of rehabilitation. The ρ indices and *P* values were derived from Pearson correlation analysis. Blue line and shaded area represent ρ and its 95%CI.

© 2022 Chen N et al. *JAMA Network Open.*

eFigure 22. Correlations Between Estimated Annual Percentage Change and Neck Pain Age-Standardized Rates

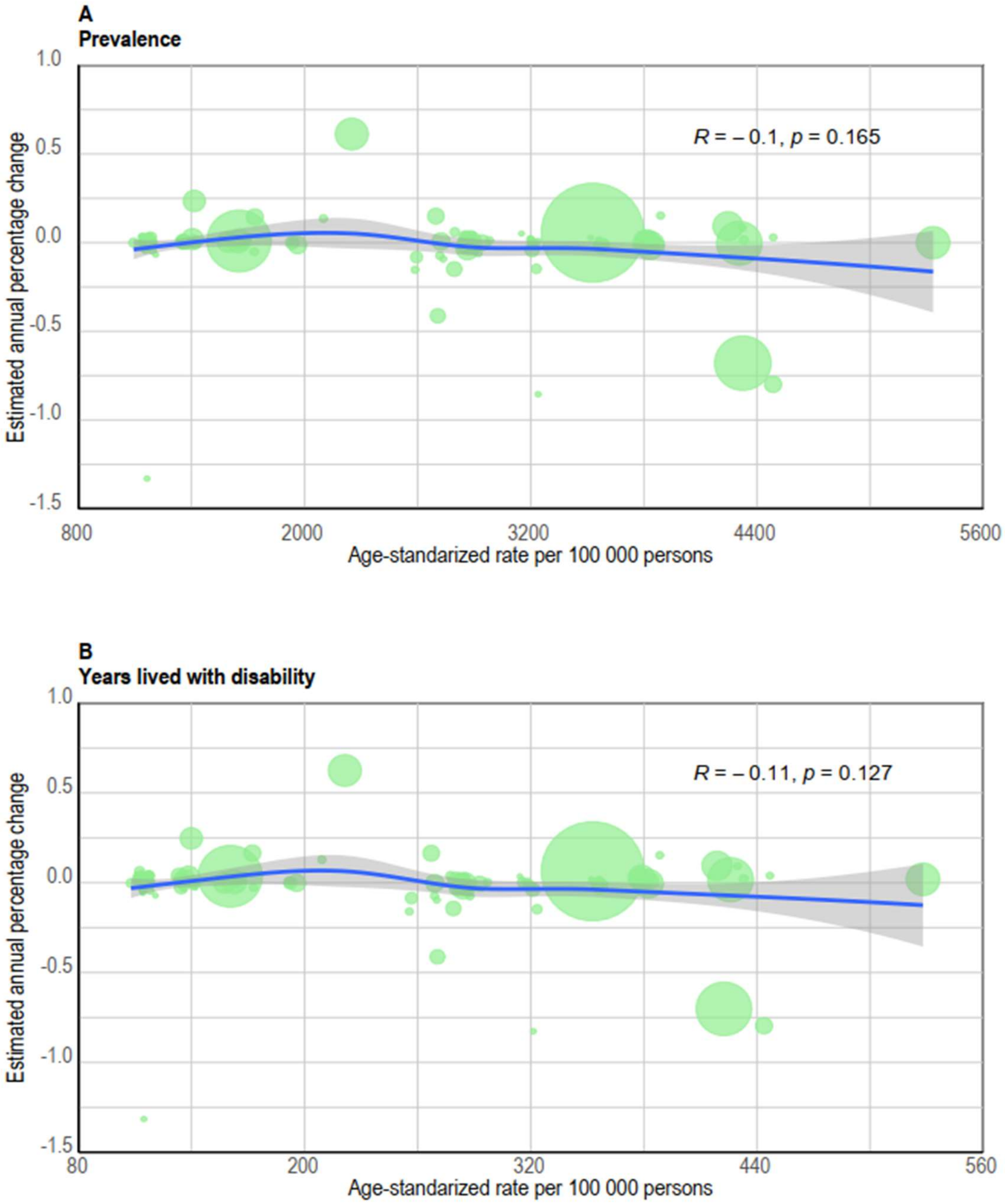

Correlation with age-standardized prevalence rate (A), and age-standardized years lived with disability (YLDs) rate (B). The size of green circle represents increases in the corresponding prevalent cases or YLDs counts of neck pain in need of rehabilitation. The ρ indices and *P* values were derived from Pearson correlation analysis. Blue line and shaded area represent ρ and its 95%CI.

© 2022 Chen N et al. *JAMA Network Open.*

eFigure 23. Correlations Between Estimated Annual Percentage Change and Fractures Age-Standardized Rates

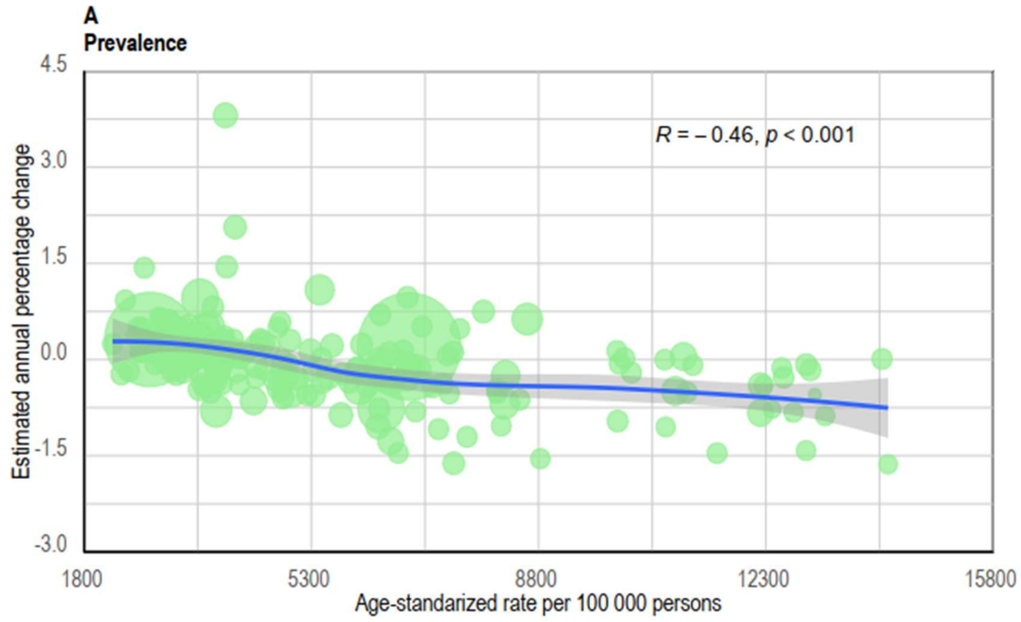

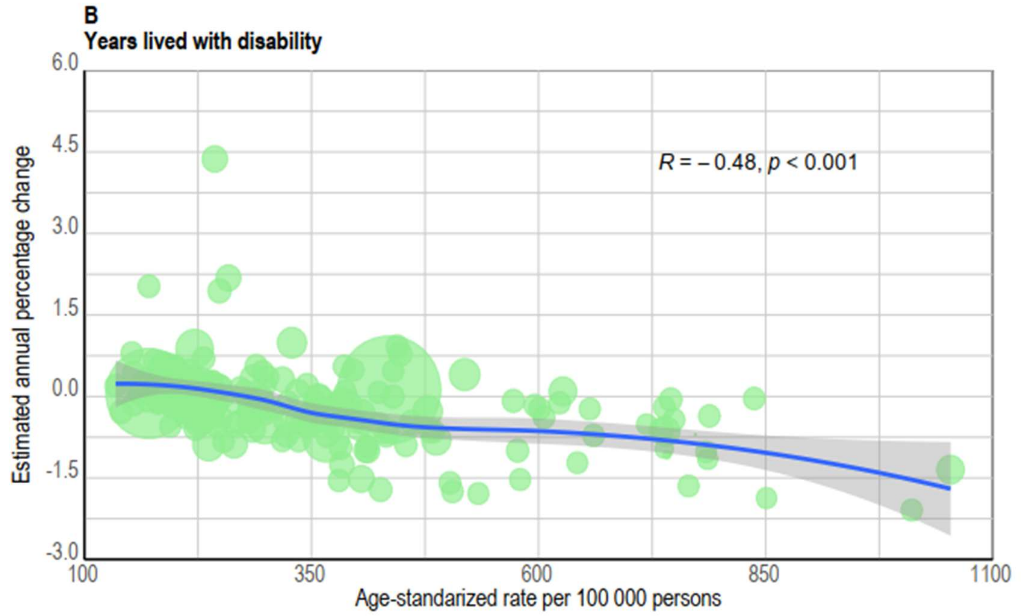

Correlation with age-standardized prevalence rate (A), and age-standardized years lived with disability (YLDs) rate (B). The size of green circle represents increases in the corresponding prevalent cases or YLDs counts of fractures in need of rehabilitation. The ρ indices and *P* values were derived from Pearson correlation analysis. Blue line and shaded area represent ρ and its 95%CI.

© 2022 Chen N et al. *JAMA Network Open.*

eFigure 24. Correlations Between Estimated Annual Percentage Change and Other Injuries Age-Standardized Rates

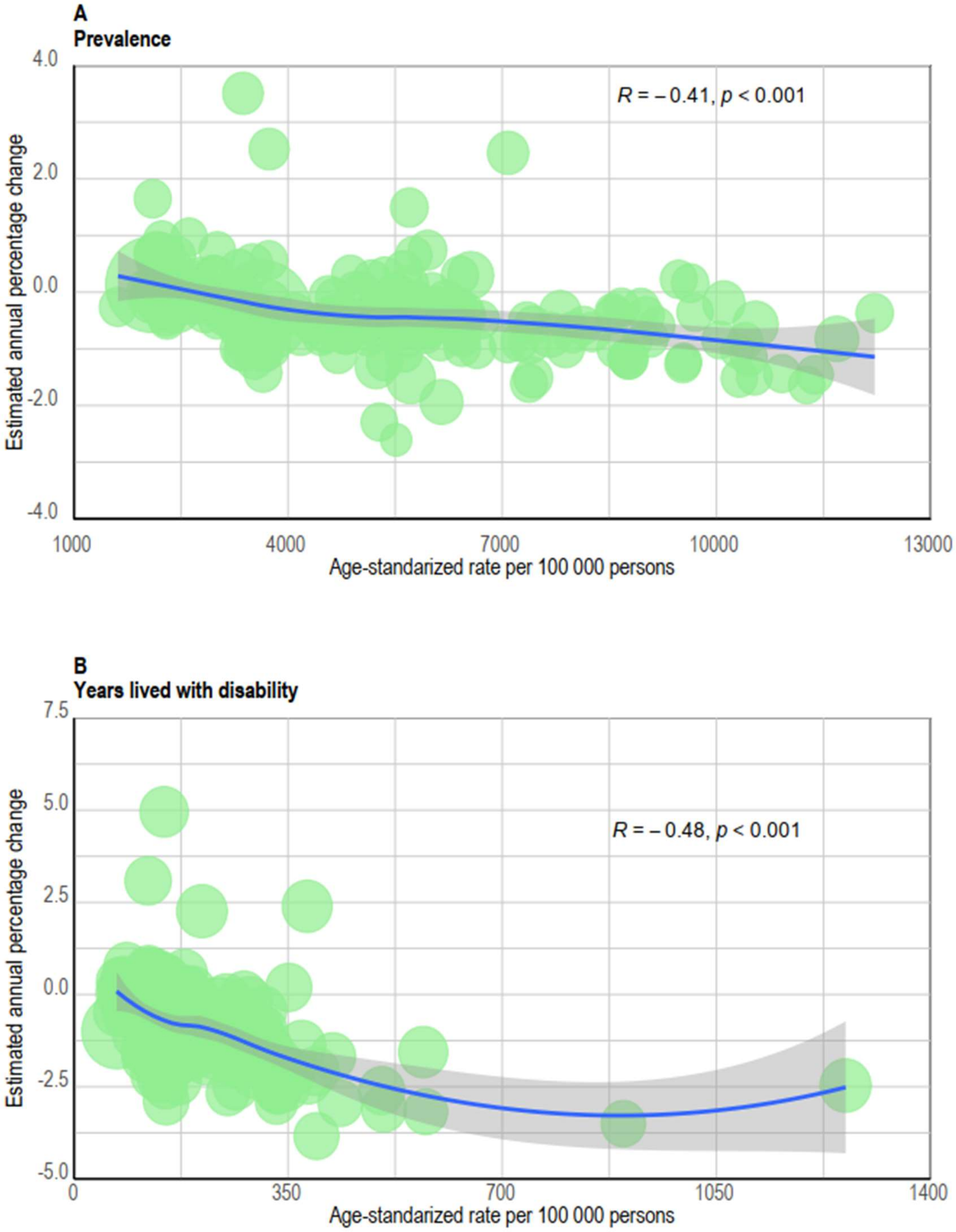

Correlation with age-standardized prevalence rate (A), and age-standardized years lived with disability (YLDs) rate (B). The size of green circle represents increases in the corresponding prevalent cases or YLDs counts of other injuries in need of rehabilitation. The ρ indices and *P* values were derived from Pearson correlation analysis. Blue line and shaded area represent ρ and its 95%CI.

© 2022 Chen N et al. *JAMA Network Open.*

eFigure 25. Correlations Between Estimated Annual Percentage Change and Osteoarthritis Age-Standardized Rates

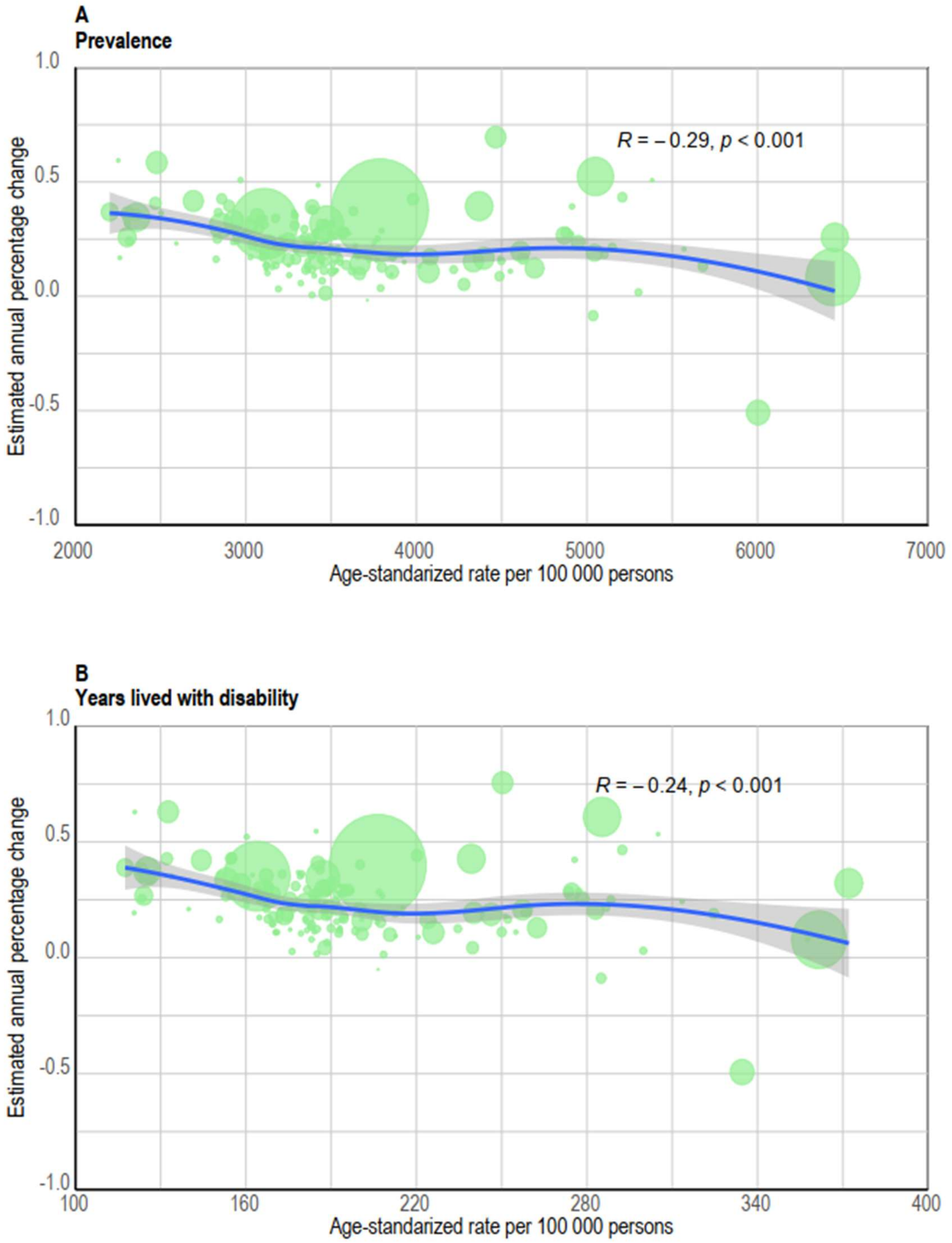

Correlation with age-standardized prevalence rate (A), and years lived with disability (YLDs) rate (B). The size of green circle represents increases in the corresponding prevalent cases or YLDs counts of osteoarthritis in need of rehabilitation. The ρ indices and *P* values were derived from Pearson correlation analysis. Blue line and shaded area represent ρ and its 95%CI.

eFigure 26. Correlations Between Estimated Annual Percentage Change and Amputation Age-Standardized Rates

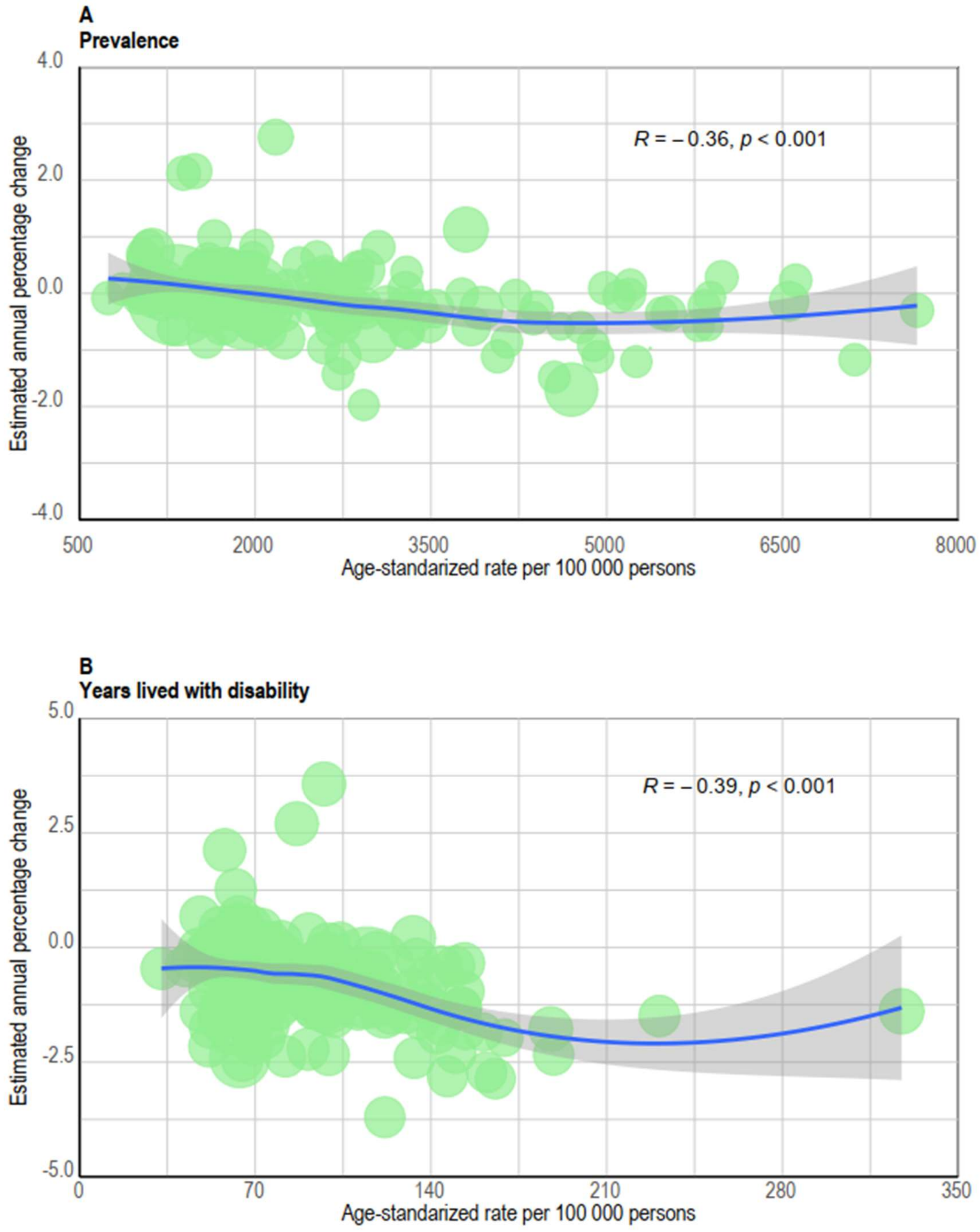

Correlation with age-standardized prevalence rate (A), and years lived with disability (YLDs) rate (B). The size of green circle represents increases in the corresponding prevalent cases or YLDs counts of amputation in need of rehabilitation. The ρ indices and *P* values were derived from Pearson correlation analysis. Blue line and shaded area represent ρ and its 95%CI.

© 2022 Chen N et al. *JAMA Network Open.*

eFigure 27. Correlations Between Estimated Annual Percentage Change and Rheumatoid Arthritis Age-Standardized Rates

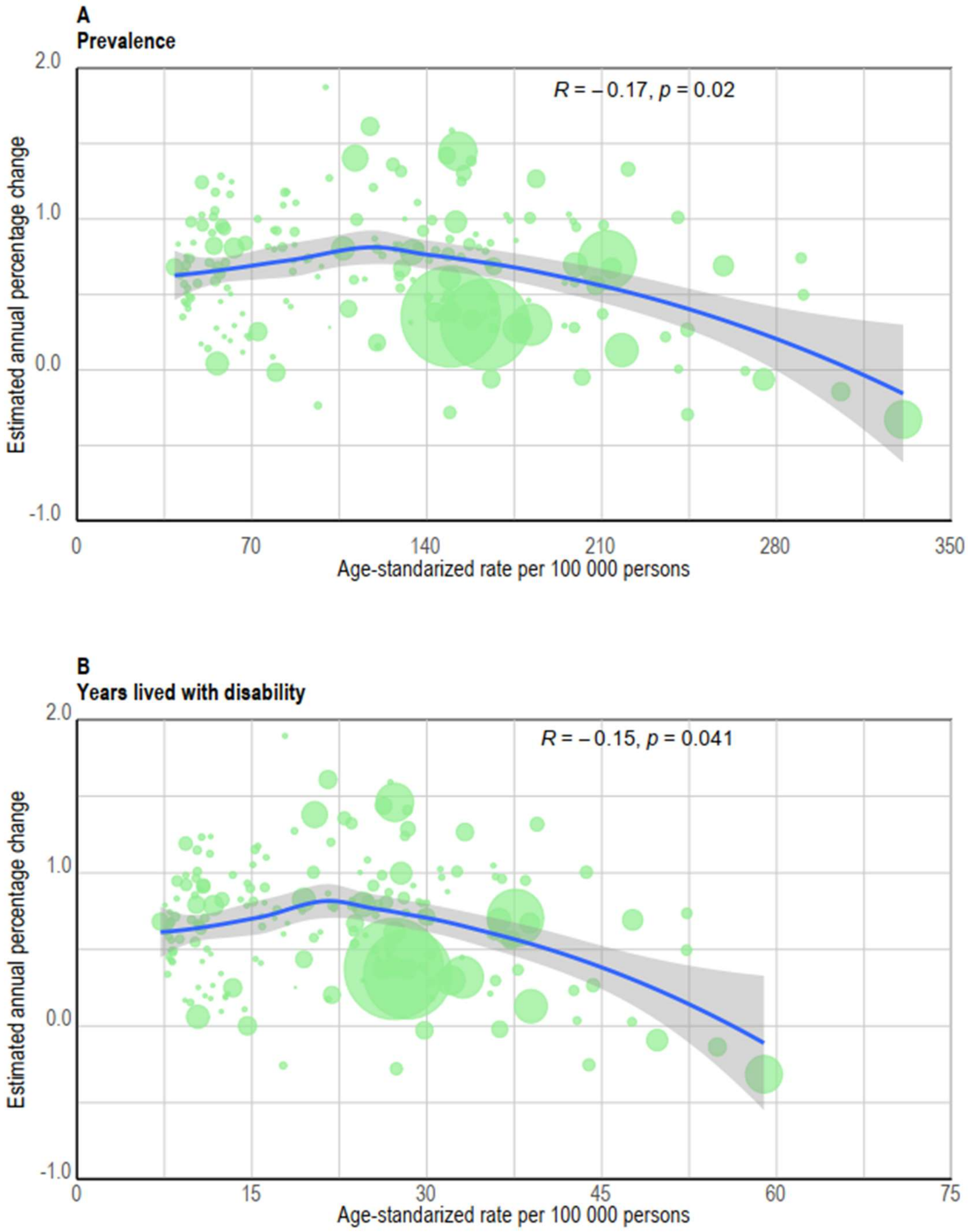

Correlation with age-standardized prevalence rate (A), and years lived with disability (YLDs) rate (B). The size of green circle represents increases in the corresponding prevalent cases or YLDs counts of rheumatoid arthritis in need of rehabilitation. The ρ indices and *P* values were derived from Pearson correlation analysis. Blue line and shaded area represent ρ and its 95%CI.

© 2022 Chen N et al. *JAMA Network Open.*

eFigure 28. Residuals for Health Spending per Capita Adjusted Linear Regression Model

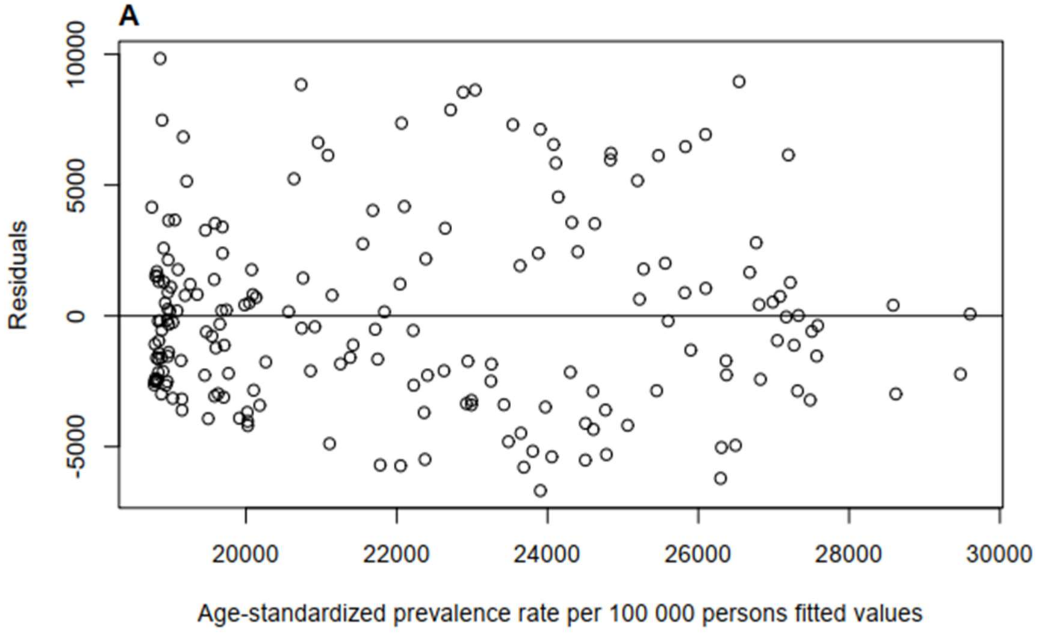

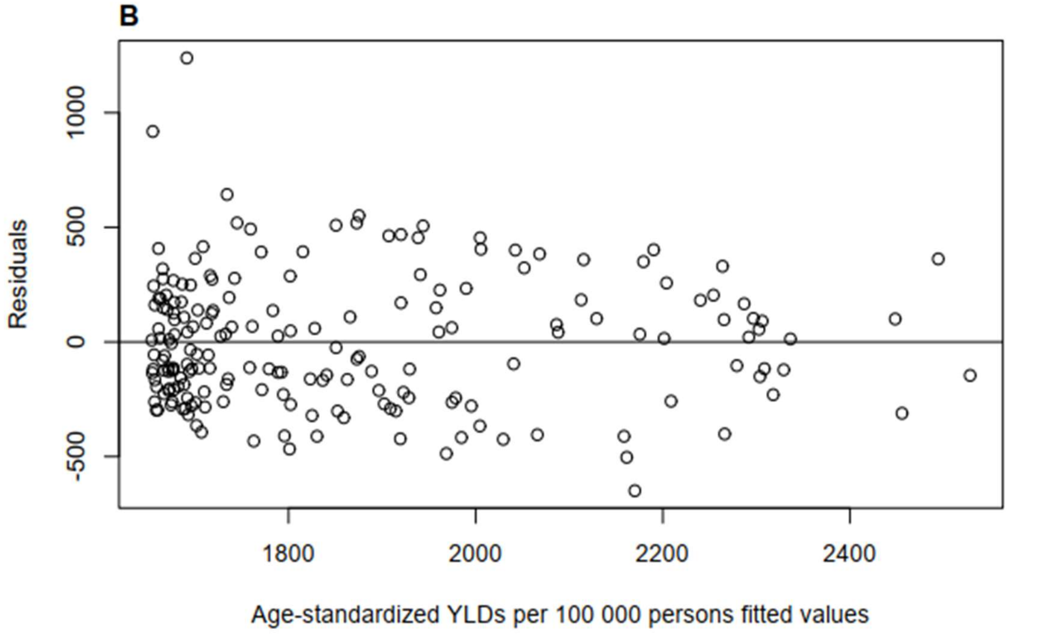

Residuals for age-standardized prevalence rate per 100 000 persons fitted values (A), and residuals for age-standardized years lived with disability (YLDs) per 100 000 persons fitted values (B).

eTable 1. Data Inputs for Injuries Incidence Modelling

Cause	The number of countries with data
Road injuries	75
Pedestrian road injuries	23
Cyclist road injuries	23
Motorcyclist road injuries	23
Motor vehicle road injuries	23
Other road injuries	19
Other transport injuries	20
Falls (Excess mortality modeling, EMR)	38
Drowning (EMR)	11
Fire, heat, and hot substances	34
Poisonings	34
Poisoning by carbon monoxide (EMR)	19
Poisoning by other means	20
Exposure to mechanical forces	23
Unintentional firearm injuries	19
Other exposure to mechanical forces	22
Adverse effects of medical treatment	44
Animal contact	31
Venomous animal contact	21
Non-venomous animal contact	21
Pulmonary aspiration and foreign body in airway	21
Foreign body in eyes	21
Foreign body in other body part	24
Environmental heat and cold exposure	24
Other unintentional injuries	21
Self-harm (EMR)	38
Self-harm by firearm (EMR)	27
Self-harm by other specified means	21
Interpersonal violence	33
Physical violence by firearm (EMR)	6
Physical violence by sharp object	25
Physical violence by other means	22

eTable 2. The List of 7 Super-Regions and 21 Regions

7 super-regions	Central Europe, Eastern Europe, and Central Asia
	High income
	Latin America and Caribbean
	North Africa and Middle East
	South Asia
	South East Asia, East Asia, and Oceania
	sub-Saharan Africa
21 regions	East Asia
	South East Asia
	Oceania
	Central Asia
	Central Europe
	Eastern Europe
	High income Asia Pacific
	Australasia
	Western Europe
	Southern Latin America
	High income North America
	Caribbean
	Andean Latin America
	Central Latin America
	Tropical Latin America
	North Africa and Middle East
	South Asia
	Central sub-Saharan Africa
	Eastern sub-Saharan Africa
	Southern sub-Saharan Africa
	Western sub-Saharan Africa

© 2022 Chen N et al. *JAMA Network Open.*

eTable 3. Population Age Standard Used in the GBD 2019 Study

Age Group	Percent of Population	Rounded
Early Neonatal	0.039706188	0.04
Late Neonatal	0.118021789	0.12
Post Neonatal	1.868264909	1.87
<1	2.025992886	2.03
1 to 4	7.909875913	7.91
5 to 9	9.568418272	9.57
10 to 14	8.990277942	8.99
15 to 19	8.324362192	8.32
20 to 24	7.866450176	7.87
25 to 29	7.632917343	7.63
30 to 34	7.331511124	7.33
35 to 39	6.811055	6.81
40 to 44	6.136798184	6.14
45 to 49	5.509495973	5.51
50 to 54	4.921822565	4.92
55 to 59	4.345633072	4.35
60 to 64	3.684473754	3.68
65 to 69	2.991239718	2.99
70 to 74	2.272487547	2.27
75 to 79	1.607371655	1.61
80 to 84	1.113034599	1.11
85 to 89	0.61707008	0.62
90 to 94	0.255008068	0.26
95 plus	0.084703935	0.08

Abbreviations: GBD, Global Burden of Diseases, Injuries, and Risk Factors Study.

© 2022 Chen N et al. *JAMA Network Open.*

eTable 4. Location Codes, Universal Health Coverage (UHC) Effective Coverage Index, and Health Spending per Capita

Location	ISO3 code	UHC effective coverage index in 2019	Health spending per capita, 2017 measured, 2019 purchasing-power parity-adjusted $
Afghanistan	AFG	39.2	203
Albania	ALB	69.7	933
Algeria	DZA	64.8	988
American Samoa	ASM	53.3	694
Andorra	AND	91.9	9712
Angola	AGO	39.1	205
Antigua and Barbuda	ATG	59.7	1063
Argentina	ARG	61.4	2006
Armenia	ARM	62.4	966
Australia	AUS	89.5	5181
Austria	AUT	86.5	5391
Azerbaijan	AZE	48.2	1268
Bahrain	BHR	70.6	2422
Bangladesh	BGD	53.7	107
Barbados	BRB	61.3	1224
Belarus	BLR	70.5	1173
Belgium	BEL	87.5	4995
Belize	BLZ	54.3	505
Benin	BEN	44.7	97
Bermuda	BMU	77.7	4430
Bhutan	BTN	51.4	316
Bolivia (Plurinational State of)	BOL	52.5	443
Bosnia and Herzegovina	BIH	64.3	1325
Botswana	BWA	57.5	1017
Brazil	BRA	64.8	1505
Brunei Darussalam	BRN	65.6	1919
Bulgaria	BGR	62.6	1853
Burkina Faso	BFA	41.7	116
Burundi	BDI	50	70
Cambodia	KHM	56.9	236

Cameroon	CMR	42.3	158
Canada	CAN	90.4	5410
Cabo Verde	CPV	62.3	349
Central African Republic	CAF	22.2	36
Chad	TCD	31.4	83
Chile	CHL	74.6	2365
China	CHN	69.9	875
Colombia	COL	74.5	1147
Comoros	COM	48.2	150
Congo	COG	43.9	175
Costa Rica	CRI	79.1	1408
Cote d'Ivoire	CIV	42.7	195
Croatia	HRV	79	1680
Cuba	CUB	72.5	3262
Cyprus	CYP	79.6	1780
Czechia	CZE	82.1	2694
Denmark	DNK	84.3	5364
Djibouti	DJI	45.4	104
Dominica	DMA	51.9	699
Dominican Republic	DOM	52.7	1037
Democratic Republic of the Congo	COD	45.1	30
Ecuador	ECU	64.5	996
Egypt	EGY	54.7	675
El Salvador	SLV	61.7	650
Equatorial Guinea	GNQ	50.1	711
Eritrea	ERI	42.3	33
Estonia	EST	82	2164
Eswatini	SWZ	53.3	773
Ethiopia	ETH	46.5	81
Micronesia (Federated States of)	FSM	34.3	136
Fiji	FJI	45.2	356
Finland	FIN	91.4	4298
France	FRA	91	5100
Gabon	GAB	52.9	702
Georgia	GEO	55.9	870
Germany	DEU	86.5	5864
Ghana	GHA	49.2	208
Greece	GRC	80.3	2368
Grenada	GRD	50.5	772
Guam	GUM	63.8	1468
Guatemala	GTM	52.2	493
Guinea	GIN	32.4	110
Guinea-Bissau	GNB	35.8	142

© 2022 Chen N et al. *JAMA Network Open.*

Guyana	GUY	40.7	456
Haiti	HTI	35.8	117
Honduras	HND	54.5	387
Hungary	HUN	72	2157
Iceland	ISL	95.3	4680
India	IND	46.8	265
Indonesia	IDN	48.6	405
Iran (Islamic Republic of)	IRN	69.6	1763
Iraq	IRQ	57.7	609
Ireland	IRL	90.6	5433
Israel	ISR	81.6	2710
Italy	ITA	89	3535
Jamaica	JAM	56.9	583
Japan	JPN	96.4	4784
Jordan	JOR	70	643
Kazakhstan	KAZ	59.3	949
Kenya	KEN	51.7	185
Kiribati	KIR	35.7	290
Kuwait	KWT	81.9	3640
Kyrgyzstan	KGZ	52.9	256
Lao People's Democratic Republic	LAO	43.8	173
Latvia	LVA	69.8	1741
Lebanon	LBN	74.5	1481
Lesotho	LSO	38.6	336
Liberia	LBR	47.6	130
Libya	LBY	66.4	821
Lithuania	LTU	70.4	2171
Luxembourg	LUX	91.5	5928
Madagascar	MDG	39.7	81
Malawi	MWI	55.5	151
Malaysia	MYS	66.6	1190
Maldives	MDV	66.9	1479
Mali	MLI	40.8	85
Malta	MLT	82.9	4353
Marshall Islands	MHL	43.9	408
Mauritania	MRT	53.1	213
Mauritius	MUS	55.9	1309
Mexico	MEX	61.4	1158
Republic of Moldova	MDA	62.2	500
Mongolia	MNG	47.9	563
Montenegro	MNE	66	1555
Morocco	MAR	57.8	471
Mozambique	MOZ	44	91

© 2022 Chen N et al. *JAMA Network Open.*

Myanmar	MMR	46.9	279
Namibia	NAM	62.1	1100
Nepal	NPL	47.2	162
Netherlands	NLD	89.9	5753
New Zealand	NZL	83	4066
Nicaragua	NIC	57.1	516
Niger	NER	35.1	69
Nigeria	NGA	38.4	212
Democratic People's Republic of Korea	PRK	52.9	45
North Macedonia	MKD	60.8	1170
Northern Mariana Islands	MNP	60.4	752
Norway	NOR	94.4	7959
Oman	OMN	71.2	1702
Pakistan	PAK	39.3	159
Palestine	PSE	61.3	139
Panama	PAN	71.3	1883
Papua New Guinea	PNG	37.6	82
Paraguay	PRY	63.4	937
Peru	PER	76	687
Philippines	PHL	54.8	374
Poland	POL	72.7	2003
Portugal	PRT	83.8	2744
Qatar	QAT	80.7	3750
Romania	ROU	69.6	1320
Russian Federation	RUS	69.1	1537
Rwanda	RWA	59.3	133
Saint Lucia	LCA	59.2	743
Saint Vincent and the Grenadines	VCT	49.6	532
Samoa	WSM	49.8	298
Sao Tome and Principe	STP	54.7	184
Saudi Arabia	SAU	64.2	3046
Senegal	SEN	49.4	170
Serbia	SRB	63.3	1163
Seychelles	SYC	61.5	1394
Sierra Leone	SLE	42.1	223
Singapore	SGP	92.6	4393
Slovakia	SVK	78.1	2336
Slovenia	SVN	90	2974
Solomon Islands	SLB	39.3	119
Somalia	SOM	24.1	14
South Africa	ZAF	59.7	1195
Republic of Korea	KOR	89.2	2993
South Sudan	SDN	41.6	217

© 2022 Chen N et al. *JAMA Network Open.*

Spain	ESP	90.3	3526
Sri Lanka	LKA	65.6	534
Sudan	SDN	51.9	315
Suriname	SUR	50.2	1044
Sweden	SWE	90.4	5917
Switzerland	CHE	93.7	7898
Syrian Arab Republic	SYR	57.6	922
Tajikistan	TJK	47.9	247
United Republic of Tanzania	TZA	55.2	129
Thailand	THA	71.6	702
Bahamas	BHS	60.6	1967
Gambia	GMB	48.1	162
Timor-Leste	TLS	45.8	197
Togo	TGO	42.8	111
Tonga	TON	52.6	291
Trinidad and Tobago	TTO	55.6	2247
Tunisia	TUN	68.2	916
Turkey	TUR	69.2	1228
Turkmenistan	TKM	44	1417
Uganda	UGA	52.7	149
United Kingdom	GBR	88	4430
Ukraine	UKR	56.8	618
United Arab Emirates	ARE	63.2	2630
Uruguay	URY	68.8	2218
United States of America	USA	82.2	10243
Uzbekistan	UZB	42.2	479
Vanuatu	VUT	34.1	89
Venezuela (Bolivarian Republic of)	VEN	61.1	555
Viet Nam	VNM	59.7	399
Yemen	YEM	49.1	123
Zambia	ZMB	52.7	210
Zimbabwe	ZWE	54.5	285

© 2022 Chen N et al. *JAMA Network Open.*

eTable 5. The Age-Standardized Prevalence Rates (per 100 000 Persons) of Musculoskeletal Rehabilitation Needs in 2019

Location	Musculoskeletal disorders	Low back pain	Neck pain	Fractures	Other injuries	Osteoarthritis	Amputation	Rheumatoid arthritis
Afghanistan	28699.8 (23812.7 to 36624.5)	7307.0 (6427.0 to 8281.8)	2872.2 (2250.5 to 3640.5)	10103.6 (6477.6 to 16948.3)	9673.9 (5763.5 to 16504.0)	3097.0 (2454.9 to 3746.5)	3075.6 (1854.1 to 6516.0)	63.4 (54.6 to 73.3)
Albania	31034.8 (29581.8 to 32636.8)	10063.1 (8850.1 to 11433.9)	1413.3 (1123.6 to 1787.5)	10374.8 (9624.2 to 11226.0)	8407.0 (7643.0 to 9238.0)	3126.0 (2483.3 to 3781.8)	5628.6 (5109.2 to 6201.9)	118.3 (101.9 to 137.0)
Algeria	19588.8 (18466.4 to 20815.2)	7237.5 (6353.5 to 8175.9)	2865.2 (2243.8 to 3627.9)	4433.8 (4143.0 to 4786.6)	3174.8 (2861.0 to 3585.7)	3395.6 (2694.9 to 4105.0)	1662.3 (1529.3 to 1821.4)	75.7 (65.3 to 87.9)
American Samoa	20403.4 (19183.4 to 21681.6)	8832.7 (7801.3 to 9912.9)	3224.6 (2568.0 to 4104.5)	3002.0 (2781.5 to 3248.9)	3438.7 (3052.9 to 3956.4)	3707.8 (2914.1 to 4518.8)	1703.3 (1553.5 to 1891.2)	74.7 (62.8 to 89.0)
Andorra	27249.9 (25857.2 to 28793.0)	9202.2 (8033.9 to 10573.7)	3538.0 (2820.0 to 4475.8)	7655.9 (7065.3 to 8300.4)	5027.1 (4600.6 to 5569.8)	5642.7 (4525.7 to 6879.0)	2832.0 (2545.2 to 3162.7)	203.1 (174.8 to 237.0)
Angola	18676.3 (17082.8	6721.1	1153.8	4733.3	4029.9	3608.2 (2881.2	1404.9	141.8 (124.8

© 2022 Chen N et al. *JAMA Network Open.*

	to 20768.1)	(5930.2 to 7608.9)	(910.6 to 1473.0)	(3978.3 to 5873.7)	(2886.4 to 6118.5)	to 4365.7)	(1146.1 to 1984.5)	to 161.5)
Antigua and Barbuda	21203.1 (20104.7 to 22411.3)	6327.4 (5567.6 to 7140.2)	1358.4 (1071.0 to 1741.2)	4445.6 (4188.3 to 4747.2)	6044.6 (5510.8 to 6715.9)	3725.5 (2942.2 to 4478.3)	2728.2 (2477.0 to 3008.6)	160.2 (137.5 to 186.3)
Argentina	25992.0 (24736.2 to 27267.5)	7015.4 (6099.0 to 8065.2)	1934.0 (1539.4 to 2447.8)	6695.3 (6281.8 to 7229.6)	7239.5 (6639.6 to 7969.6)	5265.5 (4206.0 to 6349.6)	3470.7 (3176.1 to 3827.4)	158.8 (139.4 to 180.9)
Armenia	24565.5 (23317.1 to 25886.1)	8046.6 (7122.0 to 9105.8)	1418.1 (1129.4 to 1791.9)	6004.9 (5548.1 to 6507.6)	7516.5 (6674.5 to 8703.3)	3496.0 (2786.9 to 4239.8)	3057.7 (2729.5 to 3470.9)	118.7 (102.7 to 136.7)
Australia	33348.4 (31664.9 to 35247.8)	8785.5 (7641.1 to 10087.8)	1092.7 (861.2 to 1386.0)	11197.4 (10383.4 to 12215.0)	9719.3 (8816.3 to 10740.8)	5532.8 (4405.0 to 6673.0)	6401.1 (5732.8 to 7268.2)	237.1 (208.5 to 270.5)
Austria	26150.0 (24811.1 to 27647.9)	7788.1 (6777.7 to 8945.5)	3561.5 (2841.4 to 4510.2)	7401.3 (6834.0 to 8077.8)	5114.5 (4659.7 to 5682.5)	5438.0 (4354.7 to 6586.5)	2860.0 (2576.9 to 3199.1)	176.0 (153.8 to 203.1)
Azerbaijan	23129.6 (22009.4 to 24375.2)	7403.7 (6533.9 to	1415.7 (1126.8 to 1789.3)	5524.9 (5149.1 to 5955.2)	6914.4 (6279.4 to 7724.2)	3658.6 (2898.0 to 4423.4)	2508.1 (2293.8 to 2757.8)	99.7 (84.5 to 118.4)

© 2022 Chen N et al. *JAMA Network Open.*

		8384.5)						
Bahamas	20136.7 (19072.0 to 21221.5)	6337.2 (5536.4 to 7182.4)	1359.6 (1072.9 to 1742.6)	4052.7 (3844.7 to 4287.3)	5749.7 (5230.7 to 6372.0)	3847.0 (3039.0 to 4663.7)	1835.8 (1695.3 to 1994.9)	159.1 (137.0 to 184.8)
Bahrain	19480.9 (18353.1 to 20720.9)	7213.6 (6350.1 to 8149.0)	2679.5 (2109.3 to 3403.7)	4206.2 (3924.6 to 4598.6)	3184.9 (2871.4 to 3571.4)	3500.4 (2773.0 to 4224.7)	1732.9 (1585.8 to 1899.6)	112.3 (97.9 to 129.7)
Bangladesh	17570.1 (16630.3 to 18543.8)	6808.1 (6001.4 to 7736.0)	1411.3 (1120.1 to 1784.8)	4307.7 (4035.3 to 4584.3)	2869.2 (2579.2 to 3235.0)	3088.5 (2455.8 to 3709.0)	1495.6 (1342.8 to 1736.8)	222.7 (195.2 to 253.8)
Barbados	19575.0 (18520.6 to 20704.4)	6397.1 (5599.5 to 7262.3)	1359.4 (1072.5 to 1742.6)	3727.8 (3523.8 to 3948.7)	5184.6 (4684.9 to 5796.8)	3856.4 (3074.6 to 4666.4)	1833.8 (1680.5 to 2012.1)	201.9 (175.0 to 233.5)
Belarus	28685.9 (27440.5 to 30163.3)	8825.2 (7818.6 to 9977.1)	1419.8 (1131.9 to 1793.9)	10308.4 (9593.8 to 11219.4)	7513.3 (6856.6 to 8330.8)	4044.2 (3200.8 to 4846.3)	3967.3 (3619.9 to 4365.9)	93.0 (78.8 to 108.9)
Belgium	27827.1 (26333.3 to 29464.7)	9296.8 (8117.0 to 10584.3)	2976.2 (2347.3 to 3759.9)	8635.0 (7948.6 to 9397.4)	5504.2 (5015.2 to 6108.1)	5082.9 (4071.9 to 6159.3)	3172.3 (2845.6 to 3539.9)	188.6 (165.1 to 220.2)
Belize	20905.2 (19897.1	6502.8	1354.7	4622.0	5603.9	3865.2 (3054.5	2310.9	163.0 (139.9

© 2022 Chen N et al. *JAMA Network Open.*

	to 21987.9)	(5699.5 to 7358.5)	(1067.1 to 1736.9)	(4386.2 to 4861.8)	(5126.7 to 6212.2)	to 4658.2)	(2137.8 to 2502.2)	to 190.0)
Benin	16345.0 (15436.0 to 17291.1)	6719.0 (5925.3 to 7600.9)	1189.4 (939.5 to 1526.1)	3342.4 (3179.1 to 3517.9)	2072.9 (1899.1 to 2291.5)	3859.0 (3060.8 to 4676.3)	1530.8 (1426.2 to 1641.0)	52.2 (44.9 to 60.4)
Bermuda	21544.5 (20355.6 to 22812.9)	6483.3 (5713.7 to 7341.6)	1357.5 (1071.8 to 1740.3)	3566.5 (3362.1 to 3799.3)	6663.8 (5840.0 to 7673.5)	3972.9 (3147.5 to 4793.5)	2998.4 (2721.0 to 3327.8)	171.6 (146.5 to 203.4)
Bhutan	18860.8 (17941.2 to 19851.5)	6866.0 (6069.2 to 7792.2)	1407.3 (1117.2 to 1780.1)	5619.8 (5318.4 to 5956.9)	2888.5 (2675.7 to 3121.8)	3298.5 (2620.4 to 3985.2)	1809.8 (1691.6 to 1937.8)	224.2 (195.7 to 256.8)
Bolivia (Plurinational State of)	18591.3 (17631.7 to 19648.8)	6250.7 (5500.0 to 7048.1)	1356.3 (1069.7 to 1739.4)	3701.1 (3507.3 to 3970.1)	4401.3 (4039.3 to 4905.8)	3817.5 (3065.8 to 4619.0)	1700.8 (1577.4 to 1838.5)	188.6 (165.8 to 218.0)
Bosnia and Herzegovina	31675.3 (30142.8 to 33475.6)	9019.5 (7908.9 to 10276.9)	1414.4 (1125.1 to 1787.9)	11249.4 (10342.4 to 12341.7)	9603.4 (8545.4 to 11253.0)	3152.1 (2522.0 to 3799.6)	5407.5 (4845.1 to 6068.5)	176.2 (159.0 to 195.5)
Botswana	16224.2 (15321.8 to 17179.7)	5870.0 (5177.3 to	1153.4 (909.9 to 1471.9)	2908.5 (2746.8 to 3086.5)	2554.8 (2306.2 to 2845.6)	4149.8 (3307.9 to 5005.7)	1763.3 (1641.1 to 1913.3)	212.0 (188.0 to 239.9)

© 2022 Chen N et al. *JAMA Network Open.*

		6668.3)						
Brazil	20756.6 (19628.6 to 21964.3)	7682.1 (6735.5 to 8682.0)	2241.9 (1770.5 to 2870.6)	4307.5 (3986.2 to 4678.0)	3724.8 (3393.3 to 4153.7)	3786.1 (3004.3 to 4546.3)	2446.2 (2244.7 to 2678.2)	198.1 (176.7 to 220.4)
Brunei Darussalam	25552.8 (24263.4 to 27009.4)	8572.8 (7441.1 to 9809.8)	1716.1 (1358.4 to 2191.4)	6506.7 (6096.6 to 7034.0)	5828.2 (5293.2 to 6485.9)	5903.5 (4722.1 to 7071.6)	2886.4 (2647.1 to 3163.9)	136.5 (118.3 to 157.3)
Bulgaria	31430.7 (30003.2 to 33051.0)	9993.9 (8832.3 to 11338.8)	1413.7 (1124.8 to 1787.0)	10919.7 (10151.5 to 11837.1)	8417.5 (7647.6 to 9274.3)	3249.0 (2595.5 to 3909.7)	5550.7 (5017.6 to 6153.7)	96.4 (82.3 to 113.1)
Burkina Faso	16290.6 (15374.9 to 17252.6)	6634.6 (5842.4 to 7511.5)	1195.4 (945.0 to 1534.3)	3544.9 (3361.6 to 3745.6)	2052.1 (1889.8 to 2256.9)	3732.3 (2966.6 to 4514.8)	1501.5 (1403.4 to 1606.4)	50.4 (43.3 to 58.8)
Burundi	26011.2 (22641.5 to 30433.1)	6496.5 (5715.4 to 7394.1)	1141.8 (900.8 to 1458.7)	9008.7 (6860.8 to 11992.2)	8781.1 (5847.0 to 14045.2)	3397.0 (2689.9 to 4115.2)	2490.8 (1688.9 to 4356.7)	168.9 (150.0 to 190.7)
Cabo Verde	16321.1 (15379.6 to 17289.5)	6471.7 (5697.9 to 7333.2)	1187.3 (936.5 to 1519.4)	3024.6 (2863.7 to 3205.9)	2366.1 (2157.4 to 2611.9)	3895.3 (3104.9 to 4709.2)	1694.0 (1565.8 to 1834.7)	50.9 (43.2 to 60.4)
Cambodia	20725.2 (19214.8	8306.9	3827.0	4853.3	3582.4	2474.3 (1934.3	1443.3	71.6 (62.2 to

© 2022 Chen N et al. *JAMA Network Open.*

	to 22855.5)	(7365.1 to 9349.9)	(3027.9 to 4841.9)	(4197.5 to 6155.5)	(2808.5 to 5353.1)	to 3014.3)	(1248.4 to 1898.3)	83.3)
Cameroon	17208.9 (16269.0 to 18192.9)	7007.8 (6168.9 to 7951.1)	1185.6 (936.4 to 1520.4)	3896.8 (3699.1 to 4111.6)	2176.4 (1992.8 to 2415.5)	3967.8 (3177.3 to 4788.1)	1627.9 (1522.5 to 1746.7)	56.9 (49.1 to 66.3)
Canada	24448.2 (23175.1 to 25722.2)	8894.6 (7789.4 to 10147.9)	2942.3 (2325.8 to 3731.6)	6131.4 (5786.8 to 6530.5)	4836.2 (4427.0 to 5349.8)	4960.3 (3906.5 to 6118.7)	2419.4 (2240.5 to 2607.8)	253.4 (242.7 to 263.7)
Central African Republic	17432.3 (16260.8 to 18835.5)	6791.5 (6018.7 to 7733.9)	1151.2 (908.3 to 1470.9)	3790.4 (3365.0 to 4400.9)	3150.0 (2550.5 to 4198.5)	3488.3 (2774.3 to 4219.3)	1253.9 (1101.0 to 1547.4)	121.2 (106.9 to 137.0)
Chad	17420.4 (16400.1 to 18522.3)	7155.0 (6341.2 to 8050.6)	1179.5 (931.6 to 1516.0)	3876.7 (3550.7 to 4383.7)	2723.2 (2303.0 to 3475.9)	3531.9 (2806.3 to 4288.7)	1648.1 (1524.4 to 1805.5)	47.3 (40.3 to 54.7)
Chile	25859.0 (24610.2 to 27117.6)	6951.7 (6068.0 to 7979.6)	1932.1 (1538.8 to 2445.6)	6848.5 (6402.5 to 7352.6)	6940.1 (6348.9 to 7627.3)	5330.1 (4273.6 to 6415.5)	3453.5 (3150.1 to 3795.7)	266.9 (236.9 to 300.7)
China	17225.4 (16211.4 to 18325.4)	5134.7 (4548.5 to	3572.0 (2868.2 to 4517.6)	3514.8 (3283.8 to 3744.4)	2222.7 (2011.1 to 2509.3)	4095.5 (3235.1 to 5011.9)	1502.7 (1395.5 to 1627.6)	157.4 (141.8 to 174.7)

© 2022 Chen N et al. *JAMA Network Open.*

		5787.0)						
Colombia	21719.7 (20598.6 to 22881.1)	7819.8 (6920.3 to 8757.1)	1359.5 (1072.2 to 1743.2)	4817.5 (4513.8 to 5129.0)	5170.2 (4624.4 to 5842.7)	3725.9 (2974.3 to 4492.8)	2127.7 (1933.8 to 2343.6)	197.4 (171.8 to 227.3)
Comoros	18783.2 (17822.5 to 19829.8)	6654.8 (5845.4 to 7535.3)	1149.4 (906.9 to 1467.8)	4296.7 (4078.0 to 4542.3)	4081.7 (3649.8 to 4661.9)	3598.4 (2871.8 to 4343.9)	2020.9 (1879.6 to 2174.5)	196.4 (175.1 to 221.4)
Congo	17190.6 (16018.8 to 18498.2)	6588.2 (5812.7 to 7462.7)	1146.6 (904.9 to 1464.8)	3789.8 (3327.3 to 4468.6)	3026.7 (2433.0 to 4095.7)	3720.8 (2953.9 to 4496.3)	1251.0 (1069.8 to 1644.6)	142.8 (126.4 to 162.1)
Costa Rica	20879.0 (19826.2 to 22064.6)	6746.0 (5912.3 to 7616.2)	1359.4 (1071.9 to 1742.5)	4807.9 (4503.0 to 5145.7)	5061.2 (4491.4 to 5684.3)	3807.7 (3023.6 to 4591.7)	2152.0 (1946.7 to 2391.9)	275.9 (244.8 to 312.8)
Croatia	30364.4 (28972.7 to 31910.9)	10029.3 (8819.9 to 11394.1)	1414.1 (1124.8 to 1787.9)	11498.4 (10740.7 to 12409.2)	7227.4 (6586.9 to 7933.8)	3243.7 (2608.2 to 3887.8)	4714.1 (4292.5 to 5170.8)	174.1 (153.2 to 199.7)
Cuba	22586.6 (21528.1 to 23738.3)	6689.6 (6058.5 to 7328.3)	1356.2 (1069.5 to 1739.8)	5398.5 (5125.5 to 5693.1)	7006.0 (6300.2 to 7879.6)	3737.5 (2971.8 to 4536.3)	2476.5 (2271.3 to 2701.3)	179.0 (154.3 to 208.1)
Cyprus	27064.4 (25617.5	9756.4	3575.5	7012.2	4974.9	5188.5 (4147.7	2776.1	251.2 (220.0

© 2022 Chen N et al. *JAMA Network Open.*

	to 28605.6)	(8534.7 to 11117.0)	(2854.5 to 4529.5)	(6473.9 to 7664.3)	(4509.8 to 5512.8)	to 6296.7)	(2485.6 to 3115.3)	to 289.0)
Czechia	32299.8 (30878.9 to 33918.7)	10013.9 (8782.3 to 11365.6)	1412.7 (1123.3 to 1786.2)	11667.9 (10871.8 to 12610.7)	9024.2 (8220.9 to 10022.0)	3273.7 (2621.5 to 3943.7)	5566.4 (5025.4 to 6198.8)	133.8 (116.6 to 152.7)
Ivory Coast	16398.5 (15498.5 to 17355.9)	6694.8 (5919.3 to 7591.4)	1174.4 (926.9 to 1506.9)	3457.1 (3282.3 to 3658.0)	2094.7 (1915.4 to 2312.8)	3764.1 (2991.6 to 4549.4)	1586.5 (1480.5 to 1705.7)	52.5 (44.9 to 60.5)
North Korea	16516.6 (15460.2 to 17623.3)	7401.2 (6580.3 to 8354.4)	3168.9 (2505.7 to 4005.2)	2206.3 (2090.1 to 2317.5)	1505.4 (1398.2 to 1634.3)	3769.7 (2984.0 to 4581.5)	722.0 (679.2 to 768.6)	138.9 (121.7 to 159.2)
Democratic Republic of the Congo	16399.5 (15371.8 to 17446.4)	6689.9 (5899.4 to 7559.8)	1149.8 (906.9 to 1468.1)	3380.3 (3102.0 to 3756.2)	2614.7 (2237.5 to 3277.6)	3524.8 (2802.8 to 4268.6)	1156.0 (1052.0 to 1326.9)	121.4 (105.9 to 138.5)
Denmark	28495.4 (26907.0 to 30193.0)	11082.3 (9681.3 to 12714.3)	4477.6 (3571.2 to 5607.7)	6820.1 (6223.4 to 7528.6)	5069.4 (4597.1 to 5613.5)	5172.2 (4131.0 to 6274.2)	2752.6 (2461.0 to 3084.0)	230.7 (206.5 to 257.8)
Djibouti	17893.8 (16956.8 to 18939.4)	6060.0 (5312.9 to	1138.5 (898.1 to 1454.1)	3901.3 (3661.0 to 4176.3)	3979.7 (3539.8 to 4596.1)	3610.4 (2879.1 to 4360.0)	1947.6 (1804.4 to 2097.0)	214.1 (190.9 to 240.2)

© 2022 Chen N et al. *JAMA Network Open.*

		6869.0)						
Dominica	19966.6 (18917.1 to 21067.9)	6405.2 (5663.1 to 7220.4)	1352.8 (1067.6 to 1734.6)	4012.4 (3803.4 to 4226.9)	5558.2 (5074.2 to 6168.2)	3710.7 (2955.3 to 4445.9)	1902.8 (1755.5 to 2069.4)	146.7 (126.1 to 171.5)
Dominican Republic	20544.4 (19553.2 to 21618.6)	6186.6 (5429.5 to 6982.8)	1355.2 (1068.9 to 1738.3)	4752.9 (4529.4 to 5000.6)	5643.3 (5159.6 to 6234.2)	3756.5 (2986.8 to 4532.3)	2128.5 (1972.1 to 2304.3)	114.5 (95.4 to 137.2)
Ecuador	19572.0 (18627.9 to 20578.7)	5628.6 (5125.0 to 6222.2)	1356.8 (1069.7 to 1739.7)	4444.2 (4192.9 to 4761.2)	4765.4 (4361.9 to 5273.8)	4487.8 (3594.2 to 5360.1)	1897.1 (1753.9 to 2052.4)	234.7 (205.5 to 270.0)
Egypt	18501.1 (17446.6 to 19701.6)	7461.2 (6540.7 to 8414.6)	2834.6 (2219.4 to 3593.2)	3587.3 (3369.3 to 3862.4)	2628.1 (2372.6 to 2924.9)	3431.3 (2721.3 to 4102.5)	1334.6 (1231.6 to 1457.3)	71.2 (60.1 to 84.4)
El Salvador	23264.0 (21728.9 to 25143.6)	6960.0 (6113.4 to 7903.1)	1366.1 (1077.1 to 1750.0)	6199.8 (5436.8 to 7298.0)	6808.0 (5602.6 to 9003.6)	3799.7 (3031.2 to 4580.3)	2432.5 (2089.4 to 3080.6)	165.3 (142.1 to 194.3)
Equatorial Guinea	15572.2 (14659.3 to 16564.9)	6545.0 (5779.6 to 7365.6)	1156.3 (912.0 to 1475.0)	2867.9 (2723.3 to 3020.6)	2029.7 (1861.9 to 2248.2)	3863.4 (3078.4 to 4651.1)	1064.5 (989.6 to 1151.9)	152.4 (133.5 to 174.1)
Eritrea	22906.6 (20184.5	5701.7	1152.7	7487.8	7609.6	3483.9 (2773.9	2438.4	191.0 (170.9

© 2022 Chen N et al. *JAMA Network Open.*

	to 26576.2)	(5022.6 to 6433.1)	(909.1 to 1471.2)	(5620.5 to 10786.3)	(5241.1 to 12128.2)	to 4232.5)	(1936.7 to 3560.3)	to 214.9)
Estonia	27575.1 (26279.9 to 29038.5)	8388.2 (7424.3 to 9440.8)	1416.7 (1128.4 to 1790.3)	9117.7 (8479.0 to 9955.4)	7268.6 (6639.6 to 8201.1)	4091.1 (3247.8 to 4940.9)	3784.5 (3436.5 to 4139.4)	237.0 (215.5 to 261.2)
Eswatini	15995.8 (15063.8 to 16931.1)	5594.1 (4916.0 to 6307.5)	1157.1 (913.0 to 1476.9)	2855.0 (2688.3 to 3033.8)	2601.8 (2337.3 to 2890.2)	4252.3 (3398.3 to 5103.8)	1669.1 (1545.2 to 1806.8)	219.5 (195.1 to 247.7)
Ethiopia	18338.3 (17165.4 to 20088.2)	6234.9 (5520.4 to 7074.7)	1355.4 (1070.9 to 1746.1)	4319.9 (3838.5 to 5230.3)	4210.6 (3517.6 to 5593.5)	3546.1 (2822.4 to 4294.7)	1534.2 (1360.1 to 1875.5)	168.2 (149.9 to 188.2)
Fiji	19220.2 (18080.2 to 20421.8)	8727.6 (7699.0 to 9818.0)	3220.5 (2567.5 to 4097.1)	2853.5 (2651.8 to 3088.0)	2939.7 (2702.6 to 3256.1)	3495.4 (2755.4 to 4236.2)	1046.2 (964.7 to 1146.7)	61.5 (50.8 to 74.0)
Finland	29564.1 (28033.7 to 31322.8)	8274.6 (7315.3 to 9355.8)	4306.0 (3419.8 to 5365.8)	9800.5 (8971.9 to 10761.3)	6423.1 (5829.1 to 7096.3)	4369.4 (3511.9 to 5213.4)	3701.6 (3296.6 to 4218.8)	320.4 (290.8 to 357.1)
France	27126.2 (25739.5 to 28641.9)	9613.9 (8448.7 to	2792.7 (2251.0 to 3529.6)	7933.1 (7334.4 to 8641.1)	5450.7 (4993.9 to 5997.9)	4593.8 (3681.7 to 5537.1)	3005.5 (2706.9 to 3359.3)	174.2 (151.8 to 202.0)

© 2022 Chen N et al. *JAMA Network Open.*

		10875.4)						
Gabon	16002.6 (15034.6 to 17026.3)	6535.2 (5771.9 to 7404.7)	1150.0 (907.0 to 1469.0)	3170.4 (3011.0 to 3325.9)	2191.8 (2021.6 to 2423.9)	3921.2 (3114.4 to 4722.3)	1144.3 (1070.8 to 1227.1)	149.7 (131.7 to 171.4)
Gambia	15877.7 (14992.8 to 16803.5)	6371.1 (5615.5 to 7216.2)	1184.5 (935.7 to 1520.1)	3124.3 (2958.7 to 3306.0)	2127.1 (1929.8 to 2372.5)	3776.0 (3003.1 to 4572.1)	1533.7 (1422.1 to 1648.7)	51.2 (43.9 to 59.5)
Georgia	25876.4 (24724.2 to 27126.0)	6833.8 (6040.3 to 7771.5)	1417.5 (1129.3 to 1791.0)	8196.7 (7681.8 to 8828.6)	7830.4 (7181.5 to 8638.8)	3751.0 (2981.3 to 4572.6)	3477.0 (3186.8 to 3821.0)	110.1 (93.4 to 130.1)
Germany	26914.8 (25495.3 to 28430.4)	10403.9 (9190.3 to 11787.1)	3552.8 (2833.5 to 4497.0)	6923.8 (6365.6 to 7570.9)	5013.4 (4548.1 to 5585.6)	4226.6 (3376.0 to 5067.4)	2712.9 (2431.8 to 3026.7)	164.5 (142.5 to 192.1)
Ghana	15970.9 (15126.2 to 16832.1)	5503.8 (4932.5 to 6108.6)	1195.4 (944.9 to 1533.1)	3851.6 (3629.0 to 4101.7)	2157.9 (1977.4 to 2362.6)	3885.5 (3087.3 to 4700.5)	1641.6 (1519.9 to 1762.4)	55.4 (46.9 to 64.8)
Greece	25405.2 (23950.1 to 26969.3)	8852.2 (7725.9 to 10143.4)	3888.6 (3120.4 to 4903.0)	6522.3 (5978.3 to 7152.7)	4768.8 (4341.7 to 5301.4)	3843.5 (3084.6 to 4613.9)	2640.3 (2367.4 to 2943.4)	161.5 (142.6 to 182.3)
Grenada	20970.5 (19934.1	6389.7	1354.4	4508.9	6025.1	3651.1 (2903.3	2391.7	115.8 (97.7 to

© 2022 Chen N et al. *JAMA Network Open.*

	to 22128.6)	(5618.0 to 7208.1)	(1070.0 to 1736.9)	(4271.7 to 4765.2)	(5488.1 to 6725.9)	to 4409.7)	(2190.4 to 2615.1)	137.1)
Guam	19761.1 (18574.6 to 21001.4)	8621.6 (7640.1 to 9652.3)	3211.2 (2560.5 to 4083.7)	2902.6 (2688.7 to 3136.3)	3164.8 (2794.4 to 3617.4)	3632.7 (2860.0 to 4433.7)	1540.2 (1421.7 to 1680.4)	74.6 (62.2 to 89.1)
Guatemala	23089.2 (21903.4 to 24367.4)	7146.5 (6269.1 to 8075.6)	1362.5 (1073.7 to 1746.3)	5778.1 (5294.3 to 6453.2)	6458.3 (5697.8 to 7633.1)	3596.2 (2865.8 to 4309.9)	2805.2 (2539.5 to 3126.2)	190.9 (168.1 to 218.0)
Guinea	16446.6 (15545.4 to 17390.5)	6661.9 (5884.1 to 7535.7)	1188.0 (939.4 to 1526.5)	3518.6 (3335.1 to 3727.8)	2222.5 (2042.2 to 2443.2)	3653.7 (2899.3 to 4433.0)	1606.1 (1500.4 to 1720.1)	50.9 (43.4 to 59.7)
Guinea-Bissau	16778.2 (15881.6 to 17760.4)	6473.2 (5710.8 to 7311.0)	1193.3 (943.2 to 1531.6)	3969.0 (3734.0 to 4238.3)	2313.4 (2104.4 to 2577.4)	3675.5 (2926.1 to 4424.8)	1622.4 (1507.3 to 1732.2)	49.4 (42.4 to 57.3)
Guyana	21109.7 (20061.0 to 22198.0)	6419.9 (5656.6 to 7266.0)	1357.7 (1071.5 to 1740.4)	4904.5 (4652.4 to 5182.6)	5992.1 (5484.3 to 6647.6)	3678.6 (2908.5 to 4420.3)	2171.5 (2015.3 to 2349.8)	110.6 (94.4 to 130.2)
Haiti	26368.9 (24260.7 to 28716.5)	6483.4 (5701.1 to	1359.5 (1071.7 to 1742.7)	5208.1 (4601.6 to 6096.3)	10875.9 (8892.2 to 13353.6)	3343.2 (2641.0 to 4041.8)	3686.1 (2820.6 to 5257.1)	150.3 (131.2 to 171.5)

© 2022 Chen N et al. *JAMA Network Open.*

		7364.8)						
Honduras	21842.1 (20744.5 to 22968.0)	7023.4 (6132.1 to 7903.0)	1359.5 (1071.8 to 1742.6)	4546.6 (4277.8 to 4837.7)	6095.3 (5490.9 to 6810.7)	3631.8 (2876.2 to 4388.8)	2425.8 (2179.6 to 2754.8)	323.9 (291.0 to 360.3)
Hungary	30798.3 (29335.6 to 32486.8)	10119.3 (8908.0 to 11503.5)	1415.9 (1127.2 to 1789.6)	10726.6 (9937.2 to 11702.0)	8004.5 (7285.3 to 8921.6)	3315.5 (2652.0 to 3999.4)	5075.0 (4579.9 to 5650.9)	159.6 (144.0 to 177.6)
Iceland	27514.5 (26070.9 to 29129.0)	9546.0 (8375.7 to 10936.0)	3550.7 (2830.9 to 4492.6)	6887.1 (6323.4 to 7578.9)	4957.1 (4494.4 to 5520.5)	6359.6 (5017.6 to 7783.1)	2781.7 (2495.1 to 3125.7)	177.6 (153.8 to 207.0)
India	19171.3 (18341.5 to 20051.2)	5246.5 (4613.5 to 5943.6)	1655.5 (1326.3 to 2094.8)	7227.1 (6799.5 to 7661.7)	3331.1 (3108.6 to 3595.2)	3389.2 (2700.3 to 4083.1)	1883.0 (1755.7 to 1997.1)	178.3 (160.4 to 197.4)
Indonesia	19974.3 (18838.5 to 21260.0)	8358.7 (7409.6 to 9413.4)	4302.6 (3410.3 to 5473.6)	3226.6 (3049.4 to 3418.2)	2991.4 (2739.3 to 3338.4)	2593.1 (2041.5 to 3160.7)	1818.1 (1669.7 to 1990.9)	56.8 (50.5 to 63.7)
Ira (Islamic Republic of)	22150.1 (20906.9 to 23505.1)	8486.5 (7533.8 to 9528.3)	4288.7 (3391.7 to 5448.4)	4602.9 (4247.0 to 5019.4)	3646.4 (3225.8 to 4308.1)	3578.3 (2855.6 to 4323.8)	1866.0 (1691.8 to 2078.7)	79.2 (70.5 to 88.3)
Iraq	27582.9 (24748.5	7172.3	2862.7	8733.5	8536.6	3397.2 (2705.3	3237.5	73.5 (62.7 to

© 2022 Chen N et al. *JAMA Network Open.*

	to 31361.0)	(6322.7 to 8116.5)	(2242.9 to 3626.1)	(7059.7 to 11243.9)	(6106.2 to 12835.7)	to 4070.7)	(2566.9 to 4678.7)	86.1)
Ireland	27344.5 (25889.6 to 28982.9)	10175.8 (8962.9 to 11568.8)	3567.2 (2847.2 to 4518.8)	6888.4 (6305.0 to 7612.5)	5231.5 (4718.0 to 5827.3)	4721.0 (3791.4 to 5662.3)	2787.8 (2491.9 to 3145.9)	339.6 (303.9 to 382.3)
Israel	26706.1 (25221.7 to 28318.5)	9469.7 (8308.6 to 10807.4)	3567.1 (2846.2 to 4518.6)	6436.7 (5898.7 to 7062.6)	5132.0 (4609.5 to 5806.9)	5261.0 (4191.3 to 6372.5)	2652.0 (2379.9 to 2966.6)	153.4 (131.5 to 180.1)
Italy	24653.3 (23225.8 to 26196.1)	9573.6 (8345.4 to 10925.2)	3849.0 (3080.4 to 4874.4)	5995.9 (5538.6 to 6552.6)	3529.5 (3215.8 to 3903.6)	4459.9 (3558.5 to 5372.8)	2219.9 (1992.0 to 2473.3)	162.9 (145.3 to 181.9)
Jamaica	20268.5 (19226.0 to 21501.5)	6579.2 (5783.0 to 7449.6)	1356.2 (1069.9 to 1738.5)	3900.7 (3684.3 to 4153.3)	5571.1 (5042.2 to 6260.5)	3715.7 (2963.7 to 4501.4)	2198.8 (2020.5 to 2410.6)	129.8 (110.8 to 151.5)
Japan	26106.7 (24745.5 to 27527.8)	9960.4 (8700.7 to 11329.5)	1954.6 (1544.4 to 2477.9)	5491.0 (5094.2 to 6012.7)	5656.3 (5160.8 to 6294.3)	5328.7 (4224.1 to 6376.9)	3729.7 (3378.4 to 4133.2)	179.0 (160.2 to 200.4)
Jordan	18633.2 (17564.9 to 19833.7)	7253.9 (6391.6 to	2815.8 (2209.2 to 3566.8)	3618.9 (3366.8 to 3965.5)	2787.9 (2515.1 to 3120.5)	3498.1 (2785.7 to 4201.4)	1452.1 (1338.2 to 1595.5)	74.1 (63.0 to 87.4)

© 2022 Chen N et al. *JAMA Network Open.*

		8222.1)						
Kazakhstan	24306.5 (23199.8 to 25475.1)	7627.3 (6752.7 to 8631.9)	1419.3 (1130.9 to 1793.0)	7174.8 (6712.8 to 7733.6)	6263.8 (5744.0 to 6960.1)	3641.8 (2900.9 to 4400.0)	3019.5 (2762.7 to 3283.6)	298.3 (266.1 to 333.9)
Kenya	17185.7 (16238.9 to 18170.4)	6558.9 (5792.8 to 7428.2)	1358.7 (1073.3 to 1750.2)	3574.9 (3383.2 to 3763.7)	3054.9 (2787.0 to 3411.4)	3707.4 (2955.9 to 4486.2)	1400.7 (1298.6 to 1508.6)	198.3 (177.9 to 221.0)
Kiribati	18652.0 (17496.4 to 19921.5)	9216.8 (8162.6 to 10397.2)	3248.6 (2582.5 to 4140.8)	2437.2 (2280.6 to 2614.2)	2249.7 (2067.8 to 2508.9)	3352.1 (2624.8 to 4090.9)	891.7 (831.4 to 954.3)	53.1 (44.3 to 63.8)
Kuwait	20085.0 (18963.3 to 21293.1)	7412.0 (6519.9 to 8339.5)	2167.5 (1705.4 to 2779.4)	4828.7 (4478.8 to 5258.9)	3547.5 (3167.2 to 4048.0)	3603.1 (2854.8 to 4327.2)	1821.7 (1661.4 to 2020.4)	82.2 (69.5 to 97.4)
Kyrgyzstan	22082.2 (20988.6 to 23257.9)	7283.7 (6401.3 to 8234.1)	1417.1 (1128.5 to 1790.5)	5582.9 (5230.3 to 5994.9)	5749.4 (5230.9 to 6433.8)	3404.8 (2701.9 to 4120.4)	2390.0 (2182.8 to 2613.6)	255.8 (228.3 to 288.0)
Lao People's Democratic Republic	18640.8 (17513.7 to 19815.8)	7932.6 (7015.4 to 8888.5)	3807.7 (3020.2 to 4805.7)	3368.4 (3202.7 to 3556.5)	2686.5 (2491.5 to 2941.0)	2508.3 (1965.2 to 3059.6)	1158.1 (1083.5 to 1240.3)	67.9 (58.6 to 78.8)
Latvia	27895.0 (26621.4	8739.9	1418.7	9394.5	7233.0	4088.5 (3258.0	3845.7	206.2 (183.9

© 2022 Chen N et al. *JAMA Network Open.*

	to 29276.3)	(7745.0 to 9869.0)	(1130.8 to 1792.5)	(8761.8 to 10167.5)	(6629.0 to 8031.4)	to 4947.1)	(3517.2 to 4197.0)	to 235.1)
Lebanon	21169.9 (19277.7 to 24069.0)	6493.5 (5721.5 to 7349.7)	2919.5 (2279.7 to 3701.0)	5513.8 (4449.0 to 7634.8)	4799.9 (3605.2 to 7096.7)	3429.0 (2733.2 to 4151.3)	2026.1 (1628.4 to 3040.5)	80.8 (67.8 to 95.7)
Lesotho	16285.0 (15376.8 to 17199.5)	5715.8 (5027.4 to 6494.9)	1154.8 (910.7 to 1473.0)	3044.3 (2868.9 to 3236.5)	2821.8 (2558.3 to 3134.2)	4012.8 (3210.5 to 4844.5)	1763.9 (1642.0 to 1902.7)	186.0 (164.5 to 210.6)
Liberia	17586.6 (16192.9 to 19317.7)	6462.7 (5678.4 to 7292.4)	1178.1 (930.2 to 1512.6)	4042.0 (3430.1 to 5204.7)	3170.5 (2326.5 to 4857.4)	3841.4 (3049.4 to 4629.5)	1586.9 (1413.3 to 1895.3)	52.9 (45.2 to 61.9)
Libya	21414.4 (20076.9 to 22964.5)	7103.7 (6233.9 to 8024.1)	2848.6 (2231.1 to 3608.4)	5475.9 (4952.6 to 6155.5)	4247.8 (3554.1 to 5407.5)	3461.6 (2761.7 to 4180.4)	1936.1 (1727.3 to 2256.4)	68.3 (57.4 to 80.5)
Lithuania	28152.3 (26907.3 to 29572.8)	8772.9 (7759.4 to 9949.9)	1418.9 (1130.8 to 1792.6)	10123.8 (9449.6 to 10928.4)	7238.9 (6636.5 to 8000.0)	3540.6 (2823.5 to 4263.7)	3979.0 (3656.4 to 4347.3)	217.5 (194.8 to 241.8)
Luxembourg	27212.9 (25832.4 to 28737.0)	9644.8 (8475.6 to	3551.0 (2831.3 to 4490.9)	7538.0 (6961.0 to 8223.1)	5217.8 (4733.6 to 5791.3)	4713.9 (3771.7 to 5663.4)	2875.0 (2588.1 to 3212.6)	164.6 (142.6 to 190.6)

© 2022 Chen N et al. *JAMA Network Open.*

		11049.2)						
Madagascar	17709.5 (16742.1 to 18734.8)	6422.8 (5645.7 to 7336.7)	1148.2 (905.9 to 1467.4)	3531.7 (3320.9 to 3749.5)	3879.0 (3426.6 to 4460.5)	3460.9 (2730.8 to 4216.7)	1885.3 (1738.8 to 2037.8)	169.1 (151.6 to 190.5)
Malawi	16763.9 (15815.2 to 17806.5)	6276.7 (5532.6 to 7109.2)	1151.9 (909.1 to 1471.1)	3077.2 (2896.9 to 3271.5)	3320.1 (2916.5 to 3887.5)	3613.9 (2873.3 to 4387.1)	1635.4 (1514.4 to 1773.1)	194.5 (174.0 to 217.1)
Malaysia	18674.3 (17646.9 to 19808.8)	7691.6 (6814.6 to 8681.8)	3013.6 (2369.8 to 3818.7)	4018.6 (3819.0 to 4235.6)	2743.7 (2539.4 to 3031.1)	2764.6 (2171.6 to 3358.5)	1348.2 (1258.5 to 1454.1)	67.6 (57.1 to 80.1)
Maldives	17901.5 (16804.2 to 19079.8)	7078.7 (6264.0 to 7950.9)	3758.8 (2981.0 to 4736.5)	3143.1 (2964.0 to 3337.7)	2657.2 (2427.4 to 2943.5)	2606.0 (2037.9 to 3162.3)	1271.5 (1172.6 to 1380.8)	81.6 (70.1 to 95.5)
Mali	16149.5 (15174.0 to 17174.0)	5915.9 (5209.3 to 6670.3)	1182.2 (934.0 to 1518.6)	3605.9 (3316.7 to 4029.8)	2542.9 (2183.8 to 3159.5)	3618.8 (2871.5 to 4399.4)	1552.3 (1438.2 to 1697.1)	52.5 (44.7 to 60.8)
Malta	28343.9 (26823.8 to 29955.2)	10535.2 (9284.4 to 11980.3)	3552.9 (2832.7 to 4495.0)	7586.0 (6919.8 to 8361.6)	5584.6 (5093.1 to 6196.4)	4733.2 (3785.6 to 5662.4)	3023.1 (2683.8 to 3414.6)	158.1 (137.4 to 185.5)
Marshall Islands	18792.8 (17684.7	8511.3	3213.5	3144.8	2546.6	3187.2 (2499.6	1100.2	68.9 (58.9 to

© 2022 Chen N et al. *JAMA Network Open.*

	to 19941.9)	(7507.2 to 9537.8)	(2558.4 to 4085.9)	(2951.2 to 3372.4)	(2360.0 to 2802.5)	to 3871.2)	(1024.7 to 1179.9)	81.3)
Mauritania	16595.8 (15689.5 to 17576.4)	6503.5 (5743.9 to 7416.0)	1187.5 (937.9 to 1524.8)	3666.1 (3464.0 to 3897.0)	2116.9 (1944.8 to 2317.5)	3938.9 (3144.0 to 4753.7)	1628.1 (1513.3 to 1751.3)	60.0 (51.4 to 69.6)
Mauritius	18754.9 (17617.4 to 19934.3)	7177.0 (6316.4 to 8070.6)	3809.6 (3020.2 to 4806.1)	3071.3 (2891.2 to 3281.8)	3094.3 (2790.6 to 3461.9)	2771.0 (2192.0 to 3364.4)	1737.3 (1598.7 to 1893.1)	99.4 (85.9 to 118.6)
Mexico	21662.0 (20545.4 to 22798.9)	6426.7 (5632.2 to 7282.2)	1595.9 (1258.9 to 2058.8)	4338.7 (4044.3 to 4665.3)	5037.3 (4421.4 to 5775.4)	4818.3 (3861.1 to 5820.4)	3049.0 (2777.5 to 3370.4)	310.4 (279.9 to 345.4)
Micronesia (Federated States of)	19420.6 (18258.2 to 20643.8)	8769.2 (7754.3 to 9899.1)	3223.7 (2569.1 to 4101.5)	3298.6 (3085.7 to 3548.1)	2702.3 (2480.7 to 2998.4)	3391.2 (2690.7 to 4134.5)	1179.7 (1092.4 to 1279.1)	72.3 (61.7 to 84.4)
Mongolia	24362.7 (23246.2 to 25567.6)	7270.7 (6378.8 to 8235.9)	1417.2 (1128.7 to 1790.0)	7334.5 (6863.2 to 7923.7)	6670.5 (6104.9 to 7387.7)	3403.3 (2695.1 to 4124.6)	3131.0 (2848.4 to 3446.3)	134.1 (116.9 to 154.1)
Montenegro	30848.0 (29414.6 to 32405.9)	9793.1 (8571.3 to	1414.1 (1124.9 to 1788.0)	10804.6 (10039.4 to	8293.2 (7577.6 to 9181.0)	3228.2 (2585.4 to 3909.8)	5181.3 (4703.0 to 5723.7)	112.4 (97.0 to 131.0)

© 2022 Chen N et al. *JAMA Network Open.*

		11143.0)		11710.8)				
Morocco	20498.3 (19390.2 to 21746.3)	7717.5 (6818.5 to 8677.7)	2874.2 (2251.3 to 3637.4)	4806.7 (4497.2 to 5209.4)	3465.6 (3140.5 to 3836.3)	3320.9 (2637.3 to 3992.5)	1822.2 (1671.7 to 1982.2)	65.9 (55.7 to 77.5)
Mozambique	20325.3 (19056.7 to 21924.7)	6660.4 (5837.1 to 7533.9)	1154.4 (910.9 to 1473.5)	5453.4 (4846.3 to 6487.0)	5201.7 (4335.5 to 6827.2)	3554.4 (2829.1 to 4288.4)	2108.9 (1911.8 to 2396.9)	196.4 (175.4 to 220.6)
Myanmar	20109.8 (18893.7 to 21464.1)	7162.7 (6335.2 to 8021.6)	3826.5 (3029.6 to 4836.7)	4331.3 (3992.2 to 4776.3)	3756.4 (3214.4 to 4614.2)	2531.3 (1994.6 to 3087.1)	1708.8 (1504.4 to 2014.8)	83.6 (72.6 to 96.2)
Namibia	16888.3 (15898.2 to 17893.9)	6165.0 (5429.3 to 6976.2)	1155.2 (911.6 to 1473.6)	3326.0 (3059.4 to 3694.6)	2947.1 (2550.5 to 3580.1)	3963.7 (3131.4 to 4805.1)	1767.7 (1635.0 to 1926.3)	220.6 (196.1 to 250.5)
Nepal	19866.0 (18794.6 to 21008.5)	7736.5 (6812.1 to 8799.4)	1417.5 (1127.2 to 1790.7)	5693.9 (5328.9 to 6112.1)	3297.5 (3008.1 to 3702.6)	3227.6 (2564.9 to 3886.7)	1743.2 (1589.6 to 1921.5)	220.2 (194.2 to 251.4)
Netherlands	24265.6 (22958.9 to 25658.5)	8395.5 (7270.3 to 9598.8)	3223.4 (2587.2 to 4055.5)	6164.1 (5736.1 to 6650.5)	4735.2 (4303.2 to 5249.0)	4389.3 (3517.5 to 5286.8)	2410.4 (2178.1 to 2680.3)	243.0 (214.4 to 277.7)
New Zealand	35498.2 (33899.9	8896.2	871.0	12833.8	10960.8	5485.7 (4374.2	7172.2	260.2 (233.1

© 2022 Chen N et al. *JAMA Network Open.*

	to 37469.3)	(7713.6 to 10187.3)	(702.2 to 1082.5)	(11992.3 to 13835.6)	(9942.6 to 12053.9)	to 6630.5)	(6485.7 to 8102.9)	to 290.5)
Nicaragua	22202.5 (20538.6 to 24292.7)	6926.5 (6089.0 to 7870.4)	1360.1 (1072.7 to 1743.9)	5363.2 (4551.0 to 6647.5)	6365.3 (5202.4 to 8383.2)	3699.5 (2937.4 to 4489.4)	2249.4 (1899.6 to 2951.7)	208.8 (180.9 to 238.8)
Niger	15893.9 (14994.5 to 16823.8)	6568.1 (5788.3 to 7424.0)	1189.4 (939.1 to 1526.6)	3151.7 (2992.3 to 3332.0)	2117.2 (1933.5 to 2342.3)	3578.8 (2834.5 to 4344.1)	1497.0 (1395.5 to 1604.4)	49.1 (42.2 to 57.2)
Nigeria	17287.2 (16303.2 to 18317.1)	7864.3 (6958.1 to 8879.2)	1492.0 (1184.4 to 1928.4)	3099.4 (2932.9 to 3275.6)	2015.5 (1835.4 to 2243.1)	3832.9 (3051.5 to 4639.9)	1534.6 (1417.7 to 1657.3)	49.2 (43.6 to 55.2)
North Macedonia	29431.2 (28032.5 to 30914.1)	8968.3 (7873.7 to 10171.5)	1410.9 (1121.3 to 1784.4)	9851.1 (9151.8 to 10696.7)	8000.8 (7278.4 to 8825.5)	3186.5 (2543.9 to 3825.5)	5043.1 (4547.8 to 5602.2)	98.3 (84.1 to 115.7)
Northern Mariana Islands	20093.8 (18917.2 to 21340.4)	8587.2 (7540.3 to 9639.0)	3204.7 (2555.8 to 4077.5)	3428.5 (3191.9 to 3718.6)	3138.7 (2801.4 to 3572.6)	3515.5 (2794.2 to 4266.5)	1773.6 (1643.3 to 1934.7)	77.1 (64.8 to 91.6)
Norway	25636.7 (24244.6 to 27042.6)	8475.7 (7369.7 to	2660.0 (2099.6 to 3366.7)	6160.5 (5662.5 to 6722.8)	4976.7 (4547.2 to 5498.6)	5267.0 (4183.5 to 6396.6)	4013.7 (3632.0 to 4471.7)	240.3 (214.7 to 267.8)

© 2022 Chen N et al. *JAMA Network Open.*

		9664.7)						
Oman	20395.0 (19290.1 to 21566.3)	7209.6 (6333.4 to 8186.6)	2675.9 (2111.4 to 3394.5)	5474.6 (5127.2 to 5919.6)	3241.1 (2891.3 to 3628.8)	3426.2 (2723.9 to 4106.0)	1909.2 (1755.6 to 2089.5)	68.6 (58.6 to 81.4)
Pakistan	16675.8 (15732.6 to 17655.2)	5836.3 (5037.0 to 6717.3)	1653.9 (1324.5 to 2093.2)	4585.8 (4315.1 to 4872.6)	2521.0 (2335.1 to 2735.1)	2983.6 (2341.4 to 3635.7)	1349.7 (1260.5 to 1437.9)	223.1 (200.7 to 247.0)
Palestine	25714.5 (22899.2 to 29371.0)	7102.3 (6257.6 to 7999.9)	2868.0 (2248.4 to 3630.2)	7787.6 (6111.2 to 10185.6)	7279.6 (5023.5 to 11318.5)	3309.9 (2613.6 to 4003.5)	2761.5 (1995.7 to 4370.5)	71.9 (61.4 to 84.6)
Panama	20268.2 (19227.4 to 21339.8)	6730.1 (5914.6 to 7623.5)	1355.1 (1068.5 to 1738.0)	4369.9 (4085.6 to 4702.8)	4907.0 (4404.7 to 5539.6)	3684.4 (2931.1 to 4445.7)	2078.0 (1883.2 to 2295.2)	193.2 (167.9 to 221.3)
Papua New Guinea	20343.9 (19222.5 to 21583.3)	8827.3 (7779.1 to 9984.3)	3205.5 (2552.3 to 4075.1)	3794.8 (3548.6 to 4042.5)	3776.1 (3443.9 to 4195.0)	2992.5 (2350.4 to 3627.6)	1266.7 (1161.0 to 1388.6)	55.9 (47.3 to 65.9)
Paraguay	20528.7 (19463.2 to 21709.3)	7259.5 (6398.3 to 8175.6)	1654.0 (1307.6 to 2124.4)	4835.9 (4520.2 to 5183.5)	4526.5 (4149.4 to 4991.5)	3647.8 (2921.1 to 4413.9)	1771.3 (1635.8 to 1932.4)	200.5 (173.6 to 233.2)
Peru	18988.6 (18002.1	6048.0	1356.8	3998.4	4740.7	3755.0 (2976.2	1856.4	180.4 (156.0

© 2022 Chen N et al. *JAMA Network Open.*

	to 20089.2)	(5329.4 to 6800.2)	(1069.4 to 1739.8)	(3738.3 to 4281.8)	(4289.2 to 5350.9)	to 4528.1)	(1704.8 to 2020.1)	to 208.2)
Philippines	20841.4 (19562.0 to 22277.2)	8923.4 (7900.2 to 10048.8)	5333.5 (4269.7 to 6740.7)	3523.2 (3312.3 to 3769.7)	2886.2 (2626.6 to 3251.3)	2448.7 (1932.7 to 2988.1)	1316.3 (1213.1 to 1432.4)	79.8 (71.4 to 89.0)
Poland	31058.2 (29625.0 to 32655.4)	9999.5 (8848.3 to 11293.8)	1659.7 (1330.4 to 2098.1)	11026.1 (10230.6 to 11937.4)	8159.3 (7433.0 to 9076.8)	3364.9 (2686.9 to 4053.0)	5037.1 (4569.3 to 5560.3)	206.7 (186.7 to 229.6)
Portugal	24592.9 (23325.2 to 26027.1)	10342.7 (9170.5 to 11768.2)	3583.7 (2860.4 to 4535.8)	4795.4 (4468.2 to 5179.7)	4037.2 (3677.0 to 4467.8)	4853.3 (3882.9 to 5836.2)	1997.4 (1816.4 to 2222.3)	172.4 (152.6 to 197.1)
Qatar	21276.7 (20125.8 to 22521.2)	7483.1 (6566.5 to 8458.3)	2513.1 (1977.8 to 3177.9)	5788.6 (5360.5 to 6359.1)	3574.0 (3231.5 to 3972.3)	3521.2 (2792.8 to 4243.0)	2235.0 (2040.1 to 2472.9)	74.9 (63.3 to 88.7)
South Korea	27151.2 (25856.0 to 28619.6)	8735.0 (7622.0 to 9958.8)	1719.5 (1361.9 to 2196.2)	7330.2 (6847.9 to 7941.8)	6541.5 (5975.5 to 7256.7)	6362.3 (5089.1 to 7604.6)	3286.2 (3007.5 to 3625.9)	166.7 (150.9 to 186.0)
Republic of Moldova	26282.2 (25044.9 to 27604.4)	8533.9 (7515.5 to	1417.6 (1129.3 to 1790.6)	8176.6 (7639.3 to 8845.3)	6597.2 (6047.5 to 7303.6)	4029.3 (3216.6 to 4842.9)	3389.0 (3096.8 to 3712.2)	93.3 (80.3 to 108.2)

© 2022 Chen N et al. *JAMA Network Open.*

		9634.2)						
Romania	30632.8 (29177.8 to 32165.0)	10214.8 (9029.0 to 11549.9)	1414.0 (1125.0 to 1787.3)	10354.5 (9616.1 to 11219.4)	8060.7 (7345.6 to 8945.0)	3242.9 (2580.8 to 3918.5)	5111.4 (4626.5 to 5653.2)	104.1 (89.1 to 122.4)
Russia	29952.1 (28642.2 to 31477.7)	9159.6 (8146.8 to 10277.8)	1665.5 (1335.9 to 2103.8)	10825.1 (10091.4 to 11823.7)	7127.2 (6537.1 to 7852.7)	5890.1 (4641.3 to 7280.5)	4076.3 (3741.3 to 4454.3)	124.2 (111.9 to 136.9)
Rwanda	21931.7 (20105.0 to 24140.5)	6696.0 (5930.5 to 7596.6)	1156.6 (912.4 to 1475.7)	6597.7 (5199.6 to 8716.4)	5687.2 (4517.6 to 7671.5)	3596.0 (2861.2 to 4377.1)	2346.3 (1830.5 to 3537.7)	209.8 (187.8 to 236.3)
Saint Lucia	20310.9 (19245.3 to 21445.6)	6541.2 (5738.1 to 7427.5)	1356.2 (1070.1 to 1739.0)	4114.3 (3893.7 to 4353.4)	5540.5 (5042.6 to 6175.8)	3745.6 (2959.3 to 4513.5)	2067.8 (1910.6 to 2249.2)	164.8 (143.7 to 188.9)
Saint Vincent and the Grenadines	20173.9 (19163.6 to 21292.5)	6332.2 (5557.4 to 7176.8)	1352.3 (1066.4 to 1734.1)	4120.7 (3904.5 to 4361.5)	5693.7 (5186.7 to 6315.9)	3686.8 (2924.3 to 4457.6)	2088.9 (1923.0 to 2282.0)	118.3 (100.3 to 139.4)
Samoa	20464.3 (19249.3 to 21759.4)	9238.0 (8173.1 to 10413.7)	3211.5 (2560.5 to 4085.2)	3482.2 (3245.7 to 3744.8)	3199.6 (2843.8 to 3677.2)	3440.9 (2718.0 to 4181.8)	1332.2 (1185.1 to 1531.3)	71.4 (60.9 to 84.7)
Sao Tome and	16331.0 (15448.8	5900.8	1184.2	3709.3	2225.4	3865.7 (3078.3	1824.2	54.4 (46.5 to

© 2022 Chen N et al. *JAMA Network Open.*

Principe	to 17263.2)	(5186.6 to 6668.8)	(934.6 to 1517.9)	(3514.4 to 3927.5)	(2032.3 to 2464.1)	to 4655.8)	(1693.5 to 1961.4)	63.8)
Saudi Arabia	26268.9 (24948.7 to 27948.8)	7170.1 (6283.2 to 8113.1)	2727.8 (2143.3 to 3464.9)	10090.3 (9276.0 to 11180.8)	5244.6 (4704.5 to 5856.8)	4256.3 (3381.3 to 5149.7)	3241.8 (2903.5 to 3687.1)	71.8 (60.2 to 85.3)
Senegal	15548.5 (14679.4 to 16486.2)	6011.0 (5309.2 to 6782.6)	1189.0 (939.1 to 1526.0)	3183.6 (3020.3 to 3366.3)	2044.4 (1865.6 to 2259.4)	3768.2 (2993.7 to 4539.8)	1495.7 (1388.0 to 1613.9)	52.4 (44.8 to 61.0)
Serbia	30589.6 (29144.5 to 32191.6)	9982.3 (8699.8 to 11429.2)	1413.1 (1123.8 to 1786.6)	10249.0 (9500.0 to 11085.5)	8215.4 (7455.0 to 9108.6)	3249.9 (2576.9 to 3917.5)	5079.9 (4591.5 to 5666.0)	124.3 (108.4 to 142.7)
Seychelles	18667.2 (17546.3 to 19797.5)	7294.6 (6453.1 to 8204.2)	3792.3 (3009.0 to 4785.4)	3100.3 (2920.9 to 3285.8)	2880.4 (2622.7 to 3208.7)	2795.3 (2187.4 to 3393.0)	1736.3 (1612.7 to 1882.3)	65.6 (55.7 to 78.2)
Sierra Leone	17420.4 (16234.4 to 18774.3)	6556.0 (5758.5 to 7428.3)	1181.4 (933.0 to 1516.2)	4098.6 (3664.4 to 4868.4)	2930.2 (2335.5 to 4049.1)	3675.6 (2919.5 to 4453.3)	1616.8 (1473.1 to 1824.2)	49.4 (42.2 to 57.4)
Singapore	24398.1 (23116.9 to 25798.7)	6891.4 (5969.8 to	1713.5 (1357.6 to 2184.7)	6206.3 (5785.1 to 6750.6)	6018.9 (5469.8 to 6727.9)	5767.3 (4605.7 to 6925.0)	2960.7 (2693.7 to 3285.1)	112.3 (95.1 to 131.9)

© 2022 Chen N et al. *JAMA Network Open.*

		7917.3)						
Slovakia	31599.9 (30153.6 to 33160.3)	9845.8 (8659.6 to 11135.5)	1414.5 (1125.5 to 1788.3)	11767.7 (10932.0 to 12733.2)	8349.2 (7671.0 to 9170.9)	3276.9 (2612.4 to 3932.3)	5530.2 (4994.3 to 6139.9)	105.2 (90.2 to 122.9)
Slovenia	33030.3 (31511.7 to 34693.5)	9456.6 (8280.1 to 10699.3)	1411.5 (1121.9 to 1785.2)	12724.7 (11868.6 to 13681.0)	9308.7 (8485.7 to 10343.7)	3278.2 (2625.9 to 3941.1)	6148.8 (5538.4 to 6817.7)	201.1 (177.9 to 230.0)
Solomon Islands	20508.3 (19378.6 to 21767.4)	8754.2 (7723.6 to 9881.1)	3216.9 (2561.6 to 4091.7)	4447.5 (4107.5 to 4847.6)	3256.0 (2987.7 to 3591.3)	3143.1 (2480.3 to 3815.6)	1376.4 (1252.2 to 1526.2)	75.9 (65.5 to 87.5)
Somalia	19280.8 (17964.9 to 21087.6)	6526.9 (5747.1 to 7385.8)	1153.3 (909.7 to 1471.2)	4327.7 (3760.6 to 5452.5)	4801.2 (3998.1 to 6357.8)	3490.3 (2763.8 to 4240.5)	1989.7 (1814.3 to 2268.2)	221.5 (194.4 to 250.4)
South Africa	16079.9 (15155.6 to 17041.5)	5589.9 (4943.3 to 6331.3)	1364.9 (1077.4 to 1757.5)	2805.4 (2629.5 to 2990.2)	2482.0 (2246.1 to 2770.4)	4276.8 (3401.7 to 5147.7)	1718.9 (1584.0 to 1881.4)	258.9 (232.7 to 287.1)
South Sudan	20161.0 (18762.6 to 21870.6)	6564.3 (5761.4 to 7460.4)	1145.8 (904.8 to 1466.1)	4889.7 (4284.6 to 5942.5)	5277.0 (4349.9 to 6812.6)	3509.0 (2787.3 to 4247.3)	2144.0 (1953.8 to 2444.9)	180.1 (161.2 to 202.9)
Spain	24118.6 (22686.8	7316.2	2698.6	6631.3	4615.7	5081.3 (3874.3	2603.4	161.6 (148.4

© 2022 Chen N et al. *JAMA Network Open.*

	to 25714.6)	(6451.4 to 8361.1)	(2139.9 to 3407.7)	(6081.4 to 7295.3)	(4186.8 to 5119.7)	to 6763.7)	(2330.4 to 2927.9)	to 177.8)
Sri Lanka	21210.5 (19813.1 to 22852.9)	7148.0 (6283.6 to 8056.3)	3819.2 (3025.6 to 4824.4)	5126.7 (4584.1 to 5847.0)	4532.1 (3696.7 to 6098.4)	2594.7 (2034.6 to 3147.8)	1790.0 (1559.7 to 2140.6)	67.3 (57.2 to 79.1)
Sudan	18778.0 (17570.6 to 20105.8)	7006.6 (6190.2 to 7926.1)	2852.1 (2234.1 to 3611.6)	3704.2 (3303.3 to 4321.3)	2801.3 (2316.1 to 3700.3)	3234.8 (2580.7 to 3900.3)	2106.7 (1914.7 to 2338.1)	60.2 (51.3 to 70.7)
Suriname	19879.0 (18859.0 to 20969.1)	6481.6 (5707.9 to 7335.0)	1357.8 (1071.7 to 1740.4)	4063.7 (3857.5 to 4302.1)	5261.8 (4785.6 to 5873.1)	3803.9 (3020.0 to 4583.5)	1937.2 (1756.4 to 2197.1)	109.9 (92.9 to 131.1)
Sweden	26033.6 (24665.2 to 27542.3)	8461.8 (7444.5 to 9606.1)	4344.8 (3470.5 to 5510.2)	5906.9 (5413.0 to 6475.8)	5215.8 (4710.6 to 5793.2)	3645.3 (2895.0 to 4398.7)	4204.1 (3798.1 to 4666.4)	249.3 (228.2 to 273.2)
Switzerland	28991.5 (27442.8 to 30656.0)	10621.2 (9303.4 to 12069.4)	3554.8 (2835.0 to 4498.1)	8513.5 (7798.8 to 9352.0)	5511.8 (5013.3 to 6099.8)	4605.4 (3686.4 to 5533.0)	3347.4 (2999.5 to 3767.5)	213.5 (185.5 to 247.5)
Syrian Arab Republic	27225.8 (23809.9 to 31701.1)	7160.2 (6255.0 to	2889.6 (2265.1 to 3663.2)	9007.4 (6809.0 to 12071.7)	7527.4 (4984.2 to 12343.8)	3326.4 (2650.7 to 4023.5)	2727.6 (1855.3 to 4731.5)	71.7 (60.2 to 85.0)

© 2022 Chen N et al. *JAMA Network Open.*

		8108.7)						
Tajikistan	22721.3 (21559.4 to 24025.2)	7081.6 (6227.7 to 8052.9)	1413.1 (1123.6 to 1786.4)	5864.2 (5391.5 to 6400.2)	6680.6 (5957.2 to 7719.7)	3241.1 (2567.8 to 3935.8)	2514.8 (2255.8 to 2837.1)	151.5 (132.7 to 174.3)
Thailand	18669.7 (17532.7 to 19859.1)	7018.7 (6197.8 to 7916.5)	3815.2 (3023.7 to 4817.0)	3861.9 (3645.1 to 4104.3)	2641.2 (2430.9 to 2905.8)	2905.6 (2288.9 to 3547.3)	1330.2 (1243.3 to 1439.4)	124.4 (108.2 to 144.0)
Timor-Leste	21495.7 (19694.4 to 23705.9)	7346.3 (6502.2 to 8269.1)	3799.3 (3016.1 to 4798.6)	5354.5 (4395.1 to 6741.1)	5063.7 (3658.6 to 7585.3)	2365.8 (1851.6 to 2871.0)	1573.6 (1235.0 to 2332.9)	64.8 (55.7 to 75.3)
Togo	16298.3 (15410.4 to 17236.7)	6493.0 (5709.6 to 7319.6)	1194.8 (944.7 to 1533.0)	3472.4 (3293.7 to 3682.0)	2099.8 (1928.2 to 2304.0)	3749.5 (2956.2 to 4546.4)	1584.8 (1470.8 to 1702.2)	50.1 (42.8 to 58.1)
Tonga	19340.0 (18117.2 to 20558.9)	9060.0 (8012.6 to 10177.7)	3231.5 (2574.0 to 4116.2)	2828.3 (2627.7 to 3074.5)	2816.2 (2579.6 to 3126.9)	3482.0 (2729.6 to 4216.5)	1000.9 (922.7 to 1086.8)	67.1 (56.2 to 79.8)
Trinidad and Tobago	19418.7 (18336.3 to 20466.8)	6404.9 (5611.1 to 7241.0)	1355.0 (1069.2 to 1737.7)	3867.0 (3659.9 to 4093.3)	4950.2 (4496.8 to 5513.7)	3815.6 (3006.2 to 4630.8)	1771.6 (1635.3 to 1919.8)	195.8 (172.4 to 226.0)
Tunisia	19164.1 (18092.6	7022.1	2883.1	4271.7	2909.8	3361.6 (2685.6	1790.4	73.4 (62.3 to

© 2022 Chen N et al. *JAMA Network Open.*

	to 20314.9)	(6160.7 to 7987.7)	(2258.7 to 3648.4)	(4005.5 to 4616.0)	(2638.9 to 3237.8)	to 4020.3)	(1651.6 to 1947.7)	87.4)
Turkey	20486.5 (19321.1 to 21749.0)	8453.5 (7457.1 to 9478.7)	2875.0 (2252.6 to 3640.8)	4291.5 (4002.2 to 4660.8)	3291.2 (2956.4 to 3700.7)	3250.2 (2577.0 to 3935.8)	1691.6 (1554.8 to 1859.4)	157.9 (142.3 to 174.5)
Turkmenistan	22733.9 (21661.4 to 23941.4)	7295.4 (6448.2 to 8239.6)	1414.0 (1125.7 to 1787.0)	5432.9 (5064.5 to 5871.9)	6706.2 (6094.0 to 7482.6)	3245.4 (2585.8 to 3922.2)	2670.6 (2430.5 to 2924.5)	100.2 (85.7 to 116.9)
Uganda	18377.8 (17211.6 to 19845.8)	6470.6 (5690.8 to 7340.5)	1154.3 (910.9 to 1473.0)	4146.1 (3666.9 to 4983.6)	4214.3 (3472.4 to 5609.3)	3563.4 (2830.5 to 4311.2)	1783.0 (1620.1 to 2023.6)	178.3 (159.3 to 200.4)
Ukraine	29572.7 (28253.8 to 31064.5)	9676.5 (8554.5 to 10884.9)	1666.0 (1336.3 to 2105.7)	10554.7 (9775.3 to 11583.6)	7483.9 (6889.5 to 8259.6)	3768.0 (3005.2 to 4541.6)	4141.9 (3791.1 to 4535.9)	82.3 (72.9 to 93.1)
United Arab Emirates	20028.1 (18933.7 to 21177.9)	7101.6 (6223.6 to 8047.2)	2536.2 (2000.6 to 3197.9)	5136.4 (4825.1 to 5531.3)	3309.2 (3015.0 to 3670.6)	3395.8 (2697.6 to 4082.9)	1920.4 (1765.0 to 2109.4)	65.9 (55.7 to 78.7)
United Kingdom	27236.3 (25742.8 to 28782.6)	9903.9 (8663.9 to	4501.3 (3591.7 to 5675.2)	6387.7 (5884.4 to 6976.0)	5044.0 (4592.9 to 5631.3)	4920.7 (3952.6 to 5911.9)	2802.7 (2527.7 to 3132.6)	284.3 (255.3 to 314.4)

© 2022 Chen N et al. *JAMA Network Open.*

		11282.3)						
United Republic of Tanzania	17260.2 (16260.3 to 18285.4)	6261.9 (5507.0 to 7114.1)	1150.3 (907.7 to 1470.1)	3324.2 (3125.6 to 3519.3)	3610.8 (3164.6 to 4206.7)	3658.6 (2892.2 to 4423.1)	1738.0 (1613.7 to 1879.4)	199.1 (176.6 to 226.5)
United States of America	29675.6 (28517.8 to 30885.7)	12706.0 (11718.1 to 13778.9)	5123.3 (4268.4 to 6170.4)	5807.8 (5436.4 to 6224.4)	4461.4 (4096.6 to 4894.2)	6717.6 (5401.0 to 8081.4)	3742.3 (3434.0 to 4082.0)	248.7 (231.2 to 267.8)
Uruguay	26851.9 (25599.6 to 28143.4)	7152.4 (6236.3 to 8189.7)	1936.8 (1541.1 to 2451.8)	7373.7 (6913.8 to 7902.3)	7221.8 (6658.5 to 7930.0)	5321.4 (4245.4 to 6428.1)	4040.7 (3676.3 to 4482.3)	164.3 (143.3 to 190.3)
Uzbekistan	22620.7 (21546.4 to 23757.5)	7218.8 (6369.3 to 8146.5)	1416.3 (1127.4 to 1790.2)	5842.5 (5495.2 to 6257.9)	6099.5 (5556.2 to 6785.2)	3463.8 (2750.2 to 4195.7)	2509.1 (2303.2 to 2734.6)	303.8 (273.7 to 338.2)
Vanuatu	20208.1 (19020.1 to 21509.8)	9827.5 (8723.7 to 11073.8)	3210.8 (2555.9 to 4083.0)	3163.0 (2963.7 to 3380.6)	2886.7 (2587.8 to 3288.5)	3186.0 (2499.1 to 3872.5)	1203.5 (1077.7 to 1388.4)	63.5 (54.4 to 74.7)
Venezuela (Bolivarian Republic of)	21992.0 (20921.6 to 23103.5)	6614.5 (5807.4 to 7461.0)	1357.6 (1071.1 to 1740.3)	5174.8 (4860.9 to 5524.0)	5840.6 (5240.6 to 6505.2)	3503.9 (2769.2 to 4252.6)	2794.9 (2540.7 to 3102.2)	263.3 (250.0 to 277.9)
Viet Nam	19793.7 (18684.7	7923.4	3815.9	4260.6	3165.5	2462.5 (1943.3	1508.8	78.3 (67.2 to

© 2022 Chen N et al. *JAMA Network Open.*

	to 20937.8)	(7008.7 to 8913.4)	(3022.1 to 4820.6)	(4049.1 to 4478.7)	(2922.0 to 3490.6)	to 2974.8)	(1409.3 to 1617.2)	91.2)
Yemen	20868.5 (19536.5 to 22412.3)	7208.7 (6336.0 to 8120.3)	2874.2 (2251.5 to 3638.2)	5317.2 (4745.9 to 6096.0)	4185.1 (3460.9 to 5383.8)	3025.9 (2413.2 to 3634.7)	1704.5 (1493.6 to 2099.3)	53.7 (45.7 to 63.1)
Zambia	16657.9 (15717.2 to 17688.2)	6142.7 (5395.5 to 6950.3)	1146.8 (905.1 to 1465.0)	3115.9 (2928.0 to 3298.9)	3284.1 (2904.2 to 3787.1)	3614.8 (2894.4 to 4395.8)	1659.2 (1533.4 to 1789.2)	230.1 (208.5 to 256.2)
Zimbabwe	15830.2 (14924.1 to 16828.7)	6675.0 (5906.4 to 7608.2)	1157.0 (913.5 to 1476.5)	2413.4 (2283.2 to 2550.0)	2357.5 (2141.6 to 2620.8)	3886.2 (3072.9 to 4706.1)	1459.0 (1349.0 to 1585.8)	96.2 (83.6 to 111.2)

© 2022 Chen N et al. *JAMA Network Open.*

eTable 6. The Age-Standardized YLDs Rates (per 100 000 Persons) of Musculoskeletal Rehabilitation Needs in 2019

Location	Musculoskeletal disorders	Low back pain	Neck pain	Fractures	Other injuries	Osteoarthritis	Amputation	Rheumatoid arthritis
Afghanistan	2930.1 (2009.6 to 4392.8)	803.6 (568.0 to 1075.4)	277.7 (184.5 to 403.6)	767.8 (436.8 to 1418.0)	672.8 (350.7 to 1362.0)	163.9 (83.1 to 326.4)	232.7 (91.1 to 670.8)	11.5 (7.7 to 15.7)
Albania	2388.7 (1704.6 to 3201.9)	1141.6 (793.6 to 1551.9)	141.1 (92.5 to 205.3)	586.9 (395.4 to 842.2)	219.0 (141.3 to 328.8)	171.7 (85.9 to 339.9)	106.7 (64.2 to 169.0)	21.8 (14.5 to 29.7)
Algeria	1698.9 (1210.9 to 2242.6)	816.5 (567.8 to 1094.0)	284.0 (186.9 to 411.3)	254.1 (175.1 to 353.2)	98.9 (68.8 to 137.9)	185.1 (92.9 to 369.4)	46.3 (31.5 to 66.5)	14.0 (9.2 to 19.0)
American Samoa	1841.7 (1336.1 to 2424.5)	979.4 (690.1 to 1307.1)	316.6 (207.2 to 455.4)	176.8 (124.7 to 242.4)	94.8 (65.7 to 132.1)	197.8 (98.1 to 398.9)	62.5 (44.7 to 84.0)	13.7 (9.0 to 19.1)
Andorra	2382.3 (1691.8 to 3185.7)	1038.7 (724.8 to 1423.1)	353.5 (232.6 to 512.1)	440.6 (296.6 to 632.3)	133.4 (86.2 to 199.6)	320.7 (163.4 to 652.0)	58.4 (35.7 to 91.7)	37.0 (25.0 to 51.4)
Angola	1734.9 (1267.6 to 2286.2)	751.4 (529.2 to 1006.0)	114.1 (75.6 to 165.0)	325.9 (230.8 to 459.0)	228.5 (153.4 to 340.9)	197.8 (100.6 to 390.3)	91.6 (55.3 to 164.6)	25.7 (17.2 to 35.7)
Antigua and Barbuda	1565.3 (1123.0 to 2088.7)	713.0 (500.1 to 955.7)	135.1 (88.5 to 193.8)	253.5 (172.4 to 353.2)	168.5 (111.5 to 243.6)	203.2 (102.1 to 408.3)	62.7 (41.0 to 92.0)	29.3 (19.5 to 40.1)

© 2022 Chen N et al. *JAMA Network Open.*

Argentina	1974.5 (1398.9 to 2667.5)	791.9 (551.8 to 1073.6)	192.2 (126.8 to 273.4)	382.6 (258.9 to 541.8)	200.2 (132.2 to 292.2)	298.3 (151.8 to 594.4)	80.1 (52.6 to 118.0)	29.1 (19.7 to 40.1)
Armenia	1887.3 (1365.1 to 2489.7)	908.6 (643.0 to 1220.1)	141.4 (92.4 to 204.8)	340.9 (237.0 to 476.3)	203.4 (137.2 to 291.0)	193.2 (98.0 to 386.9)	77.9 (50.5 to 115.2)	21.9 (14.8 to 30.2)
Australia	2453.4 (1733.9 to 3310.2)	986.6 (685.3 to 1334.1)	107.9 (70.8 to 155.1)	643.5 (432.0 to 918.8)	244.0 (154.5 to 371.6)	312.9 (158.4 to 634.0)	116.0 (69.0 to 185.7)	42.6 (29.4 to 57.7)
Austria	2175.6 (1545.8 to 2908.1)	874.8 (618.6 to 1184.3)	355.2 (234.1 to 515.2)	416.4 (279.1 to 596.1)	132.3 (84.8 to 199.0)	306.9 (155.4 to 620.1)	57.8 (35.2 to 91.4)	32.1 (21.7 to 43.7)
Azerbaijan	1794.4 (1290.5 to 2367.6)	836.7 (586.1 to 1129.3)	141.3 (92.2 to 204.0)	318.8 (216.5 to 450.4)	206.9 (141.5 to 291.3)	202.8 (102.9 to 409.0)	69.5 (47.8 to 98.6)	18.5 (12.2 to 25.9)
Bahamas	1550.6 (1109.4 to 2066.0)	714.4 (502.5 to 962.5)	135.2 (88.9 to 195.3)	235.0 (159.7 to 327.5)	173.1 (116.9 to 244.4)	210.6 (104.5 to 430.1)	53.1 (36.6 to 74.4)	29.3 (19.5 to 40.1)
Bahrain	1637.7 (1179.9 to 2176.9)	808.9 (568.9 to 1083.6)	264.1 (175.4 to 382.5)	233.1 (157.1 to 332.5)	83.4 (54.5 to 123.9)	188.7 (95.0 to 375.5)	39.3 (25.3 to 59.1)	20.3 (13.7 to 27.9)
Bangladesh	1562.3 (1120.9 to 2070.6)	761.0 (536.3 to 1022.9)	139.7 (91.2 to 201.4)	256.5 (176.0 to 356.8)	135.1 (97.9 to 177.2)	166.8 (85.4 to 331.7)	63.2 (44.7 to 84.6)	40.0 (27.0 to 54.5)

© 2022 Chen N et al. *JAMA Network Open.*

Barbados	1504.2 (1074.0 to 2015.1)	722.9 (505.2 to 963.1)	135.5 (89.0 to 196.0)	212.8 (143.7 to 297.0)	139.9 (91.5 to 206.1)	211.2 (106.2 to 420.3)	45.0 (29.7 to 64.9)	36.9 (24.9 to 50.2)
Belarus	2234.4 (1607.1 to 2995.7)	992.1 (702.9 to 1325.3)	141.1 (91.2 to 203.8)	581.4 (389.0 to 828.6)	194.3 (126.0 to 292.7)	224.0 (113.5 to 442.7)	84.3 (51.6 to 129.9)	17.2 (11.3 to 24.1)
Belgium	2362.6 (1675.6 to 3166.4)	1043.6 (734.2 to 1407.4)	296.1 (198.4 to 430.5)	491.7 (330.4 to 708.8)	145.7 (93.6 to 219.0)	285.5 (145.7 to 565.4)	65.7 (40.3 to 102.6)	34.2 (23.4 to 47.0)
Belize	1648.2 (1177.4 to 2187.9)	733.4 (515.3 to 982.2)	134.8 (90.4 to 195.0)	271.8 (186.6 to 376.7)	195.1 (137.9 to 266.0)	211.7 (105.9 to 422.3)	71.4 (50.0 to 97.9)	30.0 (20.4 to 41.0)
Benin	1462.9 (1043.5 to 1953.7)	755.4 (533.0 to 1014.3)	118.1 (78.2 to 170.3)	211.2 (147.9 to 289.6)	85.4 (62.1 to 112.6)	212.8 (107.5 to 418.8)	70.5 (51.6 to 90.7)	9.6 (6.5 to 13.5)
Bermuda	1520.9 (1084.2 to 2036.9)	735.1 (514.2 to 987.1)	135.8 (89.6 to 196.3)	206.0 (140.1 to 292.1)	135.1 (78.0 to 211.7)	219.0 (110.5 to 442.5)	58.3 (35.8 to 90.3)	31.7 (21.4 to 43.2)
Bhutan	1682.7 (1213.1 to 2241.8)	766.6 (541.3 to 1037.9)	139.3 (90.9 to 201.9)	343.1 (237.7 to 472.0)	141.1 (104.2 to 184.2)	178.3 (90.5 to 357.7)	74.2 (53.2 to 100.0)	40.2 (27.4 to 54.9)
Bolivia (Plurinational State of)	1527.7 (1093.4 to 2037.1)	704.0 (492.3 to 941.4)	134.8 (89.6 to 194.0)	221.6 (152.2 to 308.4)	167.7 (122.2 to 225.1)	208.9 (105.2 to 418.6)	56.5 (40.7 to 76.6)	34.3 (23.1 to 46.8)

© 2022 Chen N et al. *JAMA Network Open.*

Bosnia and Herzegovina	2391.7 (1734.9 to 3147.3)	1015.0 (714.3 to 1357.0)	140.1 (91.4 to 202.2)	649.9 (449.7 to 898.5)	264.6 (179.3 to 374.4)	171.0 (86.5 to 336.2)	119.2 (74.4 to 183.2)	32.1 (21.9 to 43.5)
Botswana	1330.5 (952.5 to 1781.4)	647.7 (460.4 to 868.3)	112.7 (74.2 to 161.4)	172.4 (118.4 to 238.4)	73.2 (49.4 to 104.4)	225.7 (115.9 to 454.0)	61.0 (44.1 to 81.5)	37.8 (26.0 to 51.6)
Brazil	1760.7 (1250.3 to 2340.9)	861.5 (607.2 to 1146.4)	221.7 (145.4 to 322.6)	253.6 (172.1 to 360.2)	100.4 (66.3 to 147.6)	206.2 (103.6 to 407.8)	81.0 (57.3 to 111.3)	36.3 (24.7 to 48.3)
Brunei Darussalam	2090.7 (1478.3 to 2827.5)	966.8 (672.3 to 1317.1)	170.2 (112.4 to 244.6)	370.8 (250.7 to 526.5)	156.7 (101.6 to 232.1)	335.2 (170.8 to 663.3)	66.1 (42.8 to 98.0)	24.8 (16.9 to 34.0)
Bulgaria	2426.0 (1737.1 to 3237.8)	1130.1 (790.0 to 1518.7)	140.6 (91.0 to 202.2)	620.4 (418.2 to 883.5)	224.3 (146.7 to 333.8)	177.7 (89.0 to 356.7)	115.2 (71.8 to 176.1)	17.8 (11.8 to 24.5)
Burkina Faso	1467.9 (1048.5 to 1950.5)	746.3 (526.1 to 1002.4)	118.9 (77.5 to 171.4)	226.3 (158.1 to 309.2)	89.0 (65.0 to 118.1)	205.5 (104.5 to 402.3)	72.7 (54.0 to 94.3)	9.3 (6.2 to 13.0)
Burundi	2573.3 (1859.4 to 3425.0)	724.9 (514.4 to 975.5)	112.6 (74.0 to 162.1)	684.1 (459.0 to 1060.5)	628.5 (369.7 to 1062.2)	185.7 (94.2 to 365.7)	207.1 (100.2 to 447.3)	30.4 (20.6 to 41.8)
Cabo Verde	1385.8 (985.3 to 1862.6)	730.6 (512.8 to 975.0)	118.3 (78.5 to 171.4)	182.5 (125.0 to 250.9)	73.5 (50.5 to 103.2)	215.4 (109.4 to 422.9)	56.0 (40.4 to 76.0)	9.4 (6.2 to 13.2)

© 2022 Chen N et al. *JAMA Network Open.*

Cambodia	2005.4 (1439.1 to 2662.8)	930.6 (647.1 to 1239.6)	379.6 (251.3 to 550.9)	312.6 (217.9 to 451.1)	167.5 (111.7 to 264.2)	132.1 (65.6 to 265.1)	69.9 (45.6 to 116.9)	13.2 (8.8 to 18.4)
Cameroon	1543.3 (1109.0 to 2060.0)	784.9 (550.1 to 1051.2)	117.4 (77.5 to 169.1)	244.3 (169.3 to 331.6)	91.7 (66.7 to 121.1)	218.2 (110.6 to 432.2)	76.4 (56.0 to 99.4)	10.4 (6.9 to 14.3)
Canada	2152.8 (1528.0 to 2868.3)	1003.8 (697.4 to 1358.8)	292.2 (191.4 to 419.7)	351.7 (237.8 to 497.0)	126.4 (81.5 to 190.9)	278.6 (140.6 to 568.0)	54.1 (33.5 to 81.8)	45.9 (31.6 to 61.6)
Central African Republic	1660.3 (1194.7 to 2205.3)	753.5 (534.2 to 1015.8)	113.1 (74.1 to 162.8)	267.2 (190.0 to 374.9)	226.3 (151.5 to 338.8)	189.7 (97.1 to 373.9)	88.6 (59.0 to 138.4)	21.8 (14.9 to 29.4)
Chad	1574.2 (1135.1 to 2085.2)	801.2 (568.5 to 1083.9)	116.8 (77.4 to 168.7)	255.1 (182.4 to 341.6)	116.5 (82.6 to 157.8)	193.2 (98.5 to 380.2)	82.8 (60.1 to 109.5)	8.7 (5.8 to 11.9)
Chile	1945.0 (1371.3 to 2622.4)	782.9 (544.7 to 1064.4)	191.6 (126.2 to 275.1)	384.0 (259.8 to 548.1)	170.0 (107.5 to 257.8)	301.3 (153.5 to 605.8)	67.0 (40.7 to 105.1)	48.2 (32.3 to 66.1)
China	1496.8 (1073.5 to 2025.7)	579.1 (411.6 to 778.1)	358.1 (234.5 to 515.9)	203.0 (136.7 to 289.4)	60.4 (39.4 to 90.1)	224.8 (112.4 to 452.3)	42.8 (28.3 to 61.4)	28.8 (19.9 to 39.0)
Colombia	1734.4 (1245.0 to 2317.2)	885.1 (618.2 to 1187.1)	135.5 (90.0 to 196.9)	273.8 (188.3 to 382.2)	147.6 (98.8 to 209.8)	203.8 (102.3 to 411.2)	52.3 (34.3 to 77.1)	36.3 (24.2 to 49.6)

© 2022 Chen N et al. *JAMA Network Open.*

Comoros	1600.2 (1149.7 to 2128.8)	749.9 (530.2 to 996.9)	114.4 (74.6 to 165.7)	271.5 (189.5 to 373.2)	138.2 (96.7 to 187.8)	198.2 (101.1 to 390.2)	92.4 (66.9 to 119.2)	35.6 (24.6 to 48.7)
Congo	1585.4 (1151.3 to 2111.4)	735.8 (520.3 to 993.0)	113.3 (74.2 to 162.0)	251.6 (181.6 to 347.3)	180.9 (122.8 to 262.3)	204.0 (103.3 to 403.3)	74.1 (47.1 to 122.1)	25.8 (17.8 to 35.2)
Costa Rica	1604.7 (1139.1 to 2138.3)	761.6 (532.3 to 1028.9)	135.2 (89.3 to 195.7)	271.3 (183.8 to 380.0)	130.3 (83.3 to 194.0)	207.9 (103.8 to 414.2)	48.3 (30.8 to 72.5)	50.2 (34.1 to 67.5)
Croatia	2443.0 (1742.2 to 3249.3)	1129.1 (791.7 to 1521.5)	140.2 (91.4 to 202.6)	669.2 (453.3 to 948.8)	199.5 (129.5 to 293.7)	176.1 (89.0 to 346.6)	97.4 (59.2 to 151.1)	31.5 (21.4 to 42.7)
Cuba	1661.1 (1188.0 to 2221.0)	753.1 (539.5 to 1003.7)	134.9 (88.8 to 195.8)	315.7 (214.8 to 443.2)	166.9 (104.1 to 256.1)	203.6 (102.7 to 408.0)	54.0 (34.2 to 81.3)	32.9 (22.3 to 45.4)
Cyprus	2374.8 (1694.4 to 3167.0)	1100.9 (774.0 to 1497.2)	357.0 (233.8 to 515.2)	396.5 (266.9 to 570.1)	127.6 (81.7 to 191.4)	292.4 (149.0 to 589.4)	55.1 (33.7 to 86.8)	45.4 (30.9 to 61.7)
Czechia	2474.5 (1768.5 to 3291.3)	1125.0 (784.8 to 1522.1)	139.7 (91.5 to 201.1)	665.2 (448.5 to 949.4)	234.4 (150.8 to 351.7)	177.1 (89.9 to 348.5)	108.6 (65.8 to 170.3)	24.4 (16.2 to 33.5)
Ivory Coast	1460.2 (1047.3 to 1937.4)	750.1 (532.6 to 1007.3)	116.3 (76.9 to 166.0)	217.9 (153.9 to 295.8)	86.6 (62.1 to 115.0)	206.6 (104.8 to 404.7)	73.2 (54.1 to 95.5)	9.6 (6.4 to 13.3)

© 2022 Chen N et al. *JAMA Network Open.*

North Korea	1607.1 (1143.8 to 2149.3)	837.9 (591.5 to 1104.2)	317.2 (208.1 to 462.1)	128.8 (87.2 to 179.0)	61.9 (44.2 to 83.3)	206.5 (103.0 to 414.4)	29.3 (20.9 to 39.4)	25.5 (17.4 to 35.0)
Democratic Republic of the Congo	1519.0 (1096.6 to 2024.9)	744.8 (524.0 to 1003.8)	113.2 (74.6 to 163.7)	222.9 (161.3 to 297.8)	159.1 (114.7 to 215.1)	192.3 (98.8 to 375.1)	64.9 (45.4 to 93.3)	21.8 (14.9 to 30.0)
Denmark	2593.9 (1849.2 to 3441.0)	1248.5 (878.3 to 1682.1)	446.5 (298.4 to 647.4)	382.9 (258.5 to 549.3)	127.4 (81.4 to 194.7)	292.7 (147.7 to 591.1)	54.3 (33.0 to 86.5)	41.5 (28.3 to 56.9)
Djibouti	1491.9 (1077.4 to 1982.7)	683.3 (478.1 to 923.0)	113.3 (74.1 to 162.4)	245.0 (172.4 to 332.9)	127.3 (88.7 to 173.1)	199.1 (100.9 to 393.9)	85.1 (62.3 to 111.6)	38.8 (26.5 to 52.5)
Dominica	1565.5 (1126.0 to 2068.1)	719.6 (508.5 to 964.8)	134.3 (88.5 to 193.9)	234.0 (160.5 to 324.8)	189.7 (132.6 to 259.2)	201.9 (102.1 to 405.7)	59.2 (41.3 to 81.4)	26.8 (17.8 to 36.8)
Dominican Republic	1602.0 (1144.9 to 2126.3)	699.3 (489.5 to 940.0)	135.1 (89.8 to 194.3)	278.8 (189.3 to 388.7)	194.4 (136.4 to 267.1)	206.0 (103.4 to 415.8)	67.3 (47.6 to 92.5)	21.1 (13.9 to 29.1)
Ecuador	1528.7 (1092.5 to 2054.2)	634.9 (450.4 to 842.9)	135.2 (89.1 to 195.5)	259.6 (176.7 to 362.9)	151.4 (105.0 to 211.2)	249.2 (125.1 to 490.7)	55.8 (39.0 to 78.2)	42.6 (29.2 to 58.4)
Egypt	1657.3 (1183.4 to 2201.6)	842.9 (590.7 to 1130.1)	281.2 (186.5 to 412.0)	205.0 (139.6 to 289.2)	87.0 (60.1 to 119.8)	187.2 (93.6 to 377.1)	40.8 (28.1 to 57.1)	13.2 (8.5 to 18.5)

© 2022 Chen N et al. *JAMA Network Open.*

El Salvador	1850.2 (1352.5 to 2419.8)	782.0 (552.1 to 1054.2)	135.2 (89.0 to 196.5)	377.8 (271.8 to 511.5)	238.0 (164.9 to 329.6)	206.4 (103.7 to 412.2)	80.6 (50.6 to 129.0)	30.2 (20.6 to 41.9)
Equatorial Guinea	1394.0 (994.0 to 1863.4)	729.2 (515.1 to 977.2)	113.9 (75.5 to 163.0)	173.9 (120.5 to 237.3)	91.5 (65.9 to 120.3)	211.5 (107.2 to 418.0)	46.4 (33.7 to 61.2)	27.4 (18.6 to 37.9)
Eritrea	1984.2 (1441.9 to 2627.7)	636.2 (449.0 to 852.1)	113.7 (74.5 to 164.2)	536.0 (356.1 to 823.5)	315.6 (193.4 to 510.6)	190.0 (97.0 to 373.4)	158.6 (89.1 to 303.6)	34.2 (23.7 to 46.6)
Estonia	2130.3 (1533.7 to 2859.7)	944.7 (665.1 to 1274.1)	141.0 (92.6 to 205.0)	512.6 (346.1 to 730.1)	184.1 (118.3 to 279.8)	227.1 (115.5 to 448.3)	77.6 (46.6 to 119.9)	43.2 (29.7 to 59.3)
Eswatini	1313.2 (943.7 to 1757.2)	614.7 (439.8 to 825.1)	112.5 (74.3 to 161.6)	171.4 (119.0 to 236.1)	78.1 (54.2 to 108.7)	231.4 (118.1 to 462.0)	66.1 (48.4 to 87.1)	39.1 (26.7 to 54.0)
Ethiopia	1661.5 (1199.2 to 2216.5)	700.1 (491.3 to 935.2)	134.5 (87.8 to 191.3)	286.8 (202.4 to 401.1)	232.2 (165.8 to 328.6)	194.9 (99.2 to 382.4)	82.6 (56.3 to 130.0)	30.5 (21.0 to 41.8)
Fiji	1811.4 (1309.6 to 2385.8)	967.9 (689.3 to 1291.2)	316.4 (206.7 to 452.7)	167.5 (115.8 to 233.3)	119.3 (86.6 to 158.2)	186.1 (93.3 to 372.8)	42.7 (31.1 to 56.7)	11.4 (7.5 to 16.0)
Finland	2458.3 (1753.0 to 3266.6)	930.2 (652.0 to 1250.4)	428.8 (288.5 to 611.2)	555.0 (371.7 to 800.2)	168.0 (108.2 to 252.0)	243.6 (122.3 to 488.4)	75.1 (45.9 to 117.4)	57.6 (40.0 to 77.8)

© 2022 Chen N et al. *JAMA Network Open.*

France	2312.5 (1643.1 to 3081.6)	1085.6 (755.3 to 1464.4)	279.2 (185.9 to 406.7)	453.2 (303.7 to 653.5)	142.0 (91.1 to 214.8)	259.0 (130.3 to 515.3)	61.6 (37.8 to 96.5)	31.8 (21.6 to 43.4)
Gabon	1435.4 (1022.5 to 1910.2)	728.7 (511.2 to 977.3)	113.4 (75.4 to 164.1)	193.7 (135.5 to 263.6)	105.4 (77.2 to 136.5)	215.3 (108.9 to 425.6)	51.9 (37.4 to 68.7)	27.0 (18.5 to 37.2)
Gambia	1395.6 (995.4 to 1860.4)	714.3 (503.4 to 961.9)	117.4 (77.2 to 168.8)	197.1 (139.7 to 266.0)	81.5 (58.4 to 108.4)	207.5 (104.6 to 411.1)	68.4 (50.1 to 88.8)	9.4 (6.3 to 13.0)
Georgia	1930.2 (1390.0 to 2566.0)	768.0 (544.0 to 1030.4)	140.9 (92.1 to 205.2)	474.5 (322.2 to 671.0)	227.8 (153.0 to 325.2)	207.2 (104.2 to 414.9)	91.6 (61.8 to 131.0)	20.2 (13.6 to 27.9)
Germany	2357.7 (1683.1 to 3150.1)	1169.0 (810.6 to 1567.0)	353.8 (235.0 to 510.1)	388.9 (261.5 to 559.5)	127.1 (81.0 to 193.3)	234.6 (118.4 to 469.7)	54.3 (32.9 to 85.6)	30.0 (20.4 to 41.6)
Ghana	1363.5 (978.0 to 1827.6)	619.0 (438.2 to 831.7)	118.9 (78.1 to 171.2)	239.6 (166.1 to 329.5)	87.4 (62.0 to 117.0)	214.5 (109.6 to 423.8)	74.0 (54.3 to 96.3)	10.1 (6.7 to 14.2)
Greece	2162.1 (1540.1 to 2848.4)	998.5 (695.2 to 1356.9)	388.1 (259.2 to 557.3)	360.5 (240.4 to 523.0)	120.5 (76.7 to 184.2)	212.6 (107.0 to 426.5)	52.2 (32.1 to 82.9)	29.6 (20.2 to 40.8)
Grenada	1594.3 (1146.3 to 2120.5)	718.3 (505.0 to 960.6)	134.3 (89.8 to 192.6)	263.6 (180.0 to 366.0)	192.2 (132.9 to 267.0)	198.5 (100.7 to 395.7)	66.1 (45.2 to 92.3)	21.3 (14.0 to 29.2)

© 2022 Chen N et al. *JAMA Network Open.*

Guam	1798.2 (1284.5 to 2376.7)	970.2 (684.9 to 1287.7)	319.8 (207.5 to 459.7)	169.6 (116.2 to 238.4)	78.2 (49.9 to 117.9)	197.2 (97.9 to 397.3)	49.3 (34.5 to 68.5)	13.9 (9.0 to 19.3)
Guatemala	1860.2 (1348.7 to 2433.2)	800.9 (562.5 to 1067.2)	134.5 (88.7 to 194.7)	352.2 (247.4 to 482.1)	253.7 (182.1 to 346.6)	194.2 (98.0 to 387.4)	90.1 (62.5 to 130.1)	34.5 (23.5 to 47.4)
Guinea	1469.9 (1054.8 to 1952.4)	748.3 (531.1 to 1004.2)	117.9 (77.6 to 169.6)	223.7 (157.3 to 300.5)	92.9 (67.9 to 122.5)	200.8 (102.0 to 397.2)	77.0 (56.9 to 99.5)	9.3 (6.2 to 13.0)
Guinea-Bissau	1491.9 (1068.5 to 1976.8)	725.1 (510.8 to 964.8)	118.1 (77.9 to 169.8)	252.6 (178.7 to 342.2)	101.1 (73.4 to 132.8)	201.7 (103.5 to 397.3)	84.2 (61.9 to 110.0)	9.1 (6.0 to 12.5)
Guyana	1660.5 (1195.7 to 2200.7)	714.2 (505.4 to 953.7)	133.3 (88.8 to 192.2)	293.1 (203.4 to 406.2)	222.9 (161.2 to 300.9)	198.2 (99.6 to 391.7)	78.6 (56.2 to 105.3)	20.1 (13.6 to 27.5)
Haiti	2124.4 (1544.6 to 2770.1)	723.1 (513.0 to 967.0)	133.8 (87.3 to 192.8)	346.3 (245.2 to 480.5)	529.8 (365.1 to 749.9)	180.3 (90.6 to 368.0)	183.8 (113.0 to 305.3)	27.2 (18.5 to 37.1)
Honduras	1764.2 (1266.3 to 2332.0)	790.3 (548.9 to 1068.8)	134.8 (88.3 to 194.7)	269.8 (187.3 to 373.5)	237.4 (169.7 to 318.0)	197.3 (99.0 to 394.2)	76.0 (54.0 to 105.1)	58.7 (39.5 to 79.3)
Hungary	2410.2 (1719.5 to 3224.3)	1142.1 (798.0 to 1543.1)	140.5 (91.7 to 203.0)	611.0 (410.8 to 875.6)	207.9 (131.7 to 314.1)	180.6 (90.3 to 362.6)	99.0 (59.1 to 155.3)	29.1 (19.6 to 39.6)

© 2022 Chen N et al. *JAMA Network Open.*

Iceland	2399.1 (1684.4 to 3241.6)	1078.3 (753.4 to 1459.8)	355.0 (232.8 to 512.2)	385.3 (259.4 to 552.0)	126.6 (81.0 to 191.0)	366.0 (185.8 to 727.2)	55.4 (33.9 to 87.3)	32.5 (22.1 to 44.9)
India	1679.0 (1222.0 to 2207.6)	575.0 (407.5 to 773.4)	161.9 (108.0 to 234.1)	453.7 (317.7 to 620.0)	180.5 (134.0 to 234.5)	180.2 (91.1 to 360.7)	96.5 (70.9 to 126.9)	31.2 (21.5 to 41.7)
Indonesia	1873.6 (1355.2 to 2465.6)	936.0 (657.5 to 1246.6)	427.5 (280.3 to 613.7)	194.8 (135.6 to 268.4)	96.1 (67.4 to 131.8)	138.7 (68.7 to 279.9)	70.0 (51.4 to 91.5)	10.5 (7.2 to 14.4)
Iran (Islamic Republic of)	2003.9 (1448.0 to 2626.7)	951.9 (668.9 to 1275.2)	423.5 (280.3 to 609.8)	261.5 (185.4 to 358.7)	108.1 (76.0 to 150.4)	195.3 (98.7 to 381.0)	49.0 (32.1 to 72.8)	14.6 (9.9 to 19.8)
Iraq	2264.3 (1679.9 to 2959.8)	802.3 (560.9 to 1078.3)	281.5 (186.8 to 409.5)	550.2 (392.9 to 767.7)	317.7 (206.3 to 472.9)	183.8 (92.7 to 367.3)	115.3 (62.9 to 222.0)	13.5 (8.9 to 18.9)
Ireland	2397.2 (1703.3 to 3193.6)	1145.7 (802.0 to 1543.4)	355.5 (236.6 to 516.7)	384.0 (257.3 to 557.5)	130.6 (83.3 to 197.7)	265.3 (135.4 to 527.0)	55.1 (33.5 to 87.0)	61.1 (42.1 to 82.7)
Israel	2296.3 (1631.0 to 3072.2)	1070.4 (748.6 to 1440.6)	356.9 (232.4 to 514.3)	360.2 (246.0 to 512.4)	128.9 (84.1 to 191.4)	298.9 (151.7 to 601.8)	52.8 (31.5 to 83.8)	28.2 (18.8 to 38.9)
Italy	2216.5 (1568.7 to 2958.8)	1078.1 (749.0 to 1460.8)	384.2 (254.2 to 546.4)	334.6 (225.1 to 482.9)	95.4 (61.8 to 141.7)	249.2 (126.2 to 494.7)	45.5 (27.9 to 71.6)	29.5 (20.4 to 40.1)

© 2022 Chen N et al. *JAMA Network Open.*

Jamaica	1548.2 (1102.6 to 2046.3)	741.4 (523.6 to 992.7)	134.9 (89.4 to 194.5)	224.4 (152.1 to 313.9)	165.1 (112.2 to 236.6)	202.8 (102.0 to 401.3)	55.7 (37.8 to 80.1)	23.9 (15.7 to 32.9)
Japan	2191.8 (1550.3 to 2942.2)	1134.3 (789.3 to 1538.0)	195.8 (129.8 to 287.9)	315.2 (212.3 to 451.1)	133.2 (83.2 to 206.3)	304.7 (153.8 to 607.8)	75.9 (46.4 to 118.1)	32.8 (22.6 to 44.6)
Jordan	1618.0 (1143.3 to 2147.8)	819.8 (570.5 to 1091.2)	279.6 (185.9 to 407.1)	203.0 (137.6 to 289.5)	76.1 (50.0 to 111.2)	191.3 (95.2 to 383.9)	34.5 (22.5 to 51.4)	13.7 (9.0 to 18.9)
Kazakhstan	1920.2 (1385.0 to 2546.1)	856.7 (606.8 to 1151.5)	140.8 (92.0 to 203.5)	408.2 (275.5 to 579.5)	179.8 (120.0 to 258.8)	200.5 (101.8 to 396.7)	81.0 (55.2 to 115.8)	53.3 (36.3 to 71.9)
Kenya	1543.0 (1110.4 to 2057.2)	734.4 (518.0 to 982.3)	134.5 (88.6 to 191.5)	223.6 (156.9 to 303.2)	147.0 (108.5 to 189.0)	203.5 (103.2 to 399.4)	64.3 (46.8 to 84.4)	35.7 (24.7 to 48.2)
Kiribati	1841.5 (1326.6 to 2423.5)	1024.2 (719.0 to 1362.9)	319.6 (209.4 to 461.4)	146.1 (101.4 to 200.7)	115.5 (85.1 to 148.1)	179.1 (90.1 to 364.5)	47.2 (34.4 to 61.6)	9.9 (6.4 to 13.8)
Kuwait	1658.1 (1187.6 to 2201.8)	835.7 (589.3 to 1121.0)	215.0 (141.0 to 314.1)	267.3 (181.5 to 379.5)	90.3 (59.3 to 133.5)	196.7 (98.5 to 392.0)	38.0 (23.1 to 60.0)	15.2 (10.0 to 21.0)
Kyrgyzstan	1775.0 (1280.4 to 2355.8)	824.6 (586.3 to 1106.8)	141.7 (91.9 to 204.3)	322.3 (218.5 to 456.5)	180.6 (124.5 to 252.9)	188.8 (95.5 to 376.9)	70.2 (48.9 to 99.2)	46.9 (31.9 to 64.2)

© 2022 Chen N et al. *JAMA Network Open.*

Lao People's Democratic Republic	1815.4 (1295.2 to 2391.0)	891.9 (627.2 to 1183.9)	379.0 (250.8 to 550.7)	206.8 (143.7 to 283.7)	136.8 (101.3 to 177.1)	134.8 (66.7 to 272.0)	53.5 (39.1 to 70.3)	12.6 (8.3 to 17.7)
Latvia	2188.1 (1560.9 to 2926.4)	981.4 (693.7 to 1320.4)	141.0 (91.8 to 204.9)	531.0 (357.5 to 751.3)	187.7 (120.8 to 280.6)	226.4 (114.3 to 445.6)	83.1 (52.2 to 128.6)	37.5 (25.7 to 50.8)
Lebanon	1715.9 (1243.9 to 2298.1)	727.2 (511.5 to 966.0)	287.8 (189.0 to 422.2)	311.8 (214.0 to 455.0)	140.5 (84.1 to 245.3)	185.7 (94.2 to 366.5)	48.0 (25.4 to 96.3)	14.8 (9.7 to 20.4)
Lesotho	1336.7 (962.5 to 1780.9)	628.3 (445.8 to 838.7)	112.3 (74.7 to 160.4)	184.3 (128.1 to 252.7)	84.9 (58.8 to 117.8)	217.7 (111.8 to 434.2)	76.0 (55.9 to 98.0)	33.1 (22.8 to 44.9)
Liberia	1535.9 (1110.5 to 2029.8)	716.1 (507.2 to 957.9)	115.4 (76.2 to 165.5)	271.6 (193.8 to 380.2)	135.2 (90.4 to 205.3)	208.8 (107.2 to 410.4)	79.1 (54.8 to 118.1)	9.6 (6.5 to 13.4)
Libya	1812.1 (1316.9 to 2375.3)	796.3 (558.9 to 1069.2)	280.7 (187.7 to 407.5)	325.5 (233.6 to 442.5)	149.1 (105.5 to 204.4)	187.6 (94.4 to 374.5)	60.3 (39.8 to 90.3)	12.6 (8.2 to 17.5)
Lithuania	2223.3 (1584.5 to 2973.5)	984.9 (695.6 to 1329.0)	140.9 (91.3 to 204.5)	578.3 (389.9 to 820.5)	194.3 (127.3 to 288.8)	194.0 (97.9 to 382.9)	91.3 (57.7 to 137.6)	39.6 (26.9 to 53.2)
Luxembourg	2349.2 (1666.6 to 3118.7)	1083.0 (759.8 to 1454.0)	353.8 (234.2 to 515.2)	426.2 (286.3 to 612.7)	134.5 (86.9 to 202.8)	263.8 (133.4 to 524.7)	58.0 (35.6 to 91.3)	29.9 (20.2 to 40.7)

© 2022 Chen N et al. *JAMA Network Open.*

Madagascar	1488.0 (1071.9 to 1968.4)	721.6 (508.1 to 975.6)	114.1 (75.3 to 163.2)	221.8 (153.4 to 306.5)	124.1 (86.9 to 169.9)	190.0 (97.1 to 374.2)	85.8 (62.5 to 111.1)	30.6 (20.6 to 41.8)
Malawi	1417.8 (1011.2 to 1885.4)	702.5 (492.2 to 944.7)	113.8 (74.5 to 163.3)	190.8 (132.7 to 264.2)	102.5 (71.1 to 142.3)	198.0 (101.2 to 391.5)	75.2 (55.2 to 97.9)	35.0 (24.3 to 48.3)
Malaysia	1684.8 (1198.0 to 2219.2)	864.2 (611.5 to 1147.3)	299.9 (200.2 to 437.4)	228.9 (155.2 to 320.9)	89.2 (61.3 to 125.0)	148.7 (73.0 to 299.7)	41.4 (28.4 to 58.0)	12.5 (8.3 to 17.5)
Maldives	1613.6 (1147.9 to 2129.0)	800.6 (558.7 to 1065.7)	376.2 (247.7 to 547.8)	176.2 (120.7 to 249.3)	74.0 (49.6 to 106.3)	141.0 (69.5 to 284.4)	30.4 (19.8 to 45.5)	15.1 (10.2 to 20.9)
Mali	1414.2 (1019.4 to 1884.7)	664.9 (468.3 to 888.1)	117.5 (77.3 to 168.3)	237.4 (169.5 to 315.7)	110.4 (77.8 to 150.5)	199.0 (100.0 to 392.2)	75.3 (55.1 to 99.4)	9.6 (6.4 to 13.5)
Malta	2461.2 (1743.4 to 3253.2)	1187.6 (830.1 to 1607.3)	354.7 (235.4 to 514.8)	424.0 (284.4 to 611.6)	140.9 (89.2 to 212.3)	266.0 (134.2 to 530.2)	59.2 (35.9 to 93.3)	28.9 (19.9 to 39.8)
Marshall Islands	1803.5 (1297.0 to 2371.5)	944.4 (669.4 to 1252.3)	315.8 (207.0 to 452.2)	187.1 (130.5 to 258.9)	121.7 (90.5 to 157.6)	169.5 (84.4 to 338.1)	52.3 (38.4 to 68.3)	12.7 (8.5 to 17.6)
Mauritania	1469.6 (1045.4 to 1973.8)	734.7 (514.6 to 995.4)	118.5 (77.4 to 171.4)	229.7 (159.7 to 313.9)	87.6 (63.1 to 117.0)	218.3 (111.1 to 432.4)	69.7 (51.3 to 91.4)	11.0 (7.4 to 15.5)

© 2022 Chen N et al. *JAMA Network Open.*

Mauritius	1646.6 (1176.8 to 2173.2)	801.1 (563.3 to 1075.9)	376.3 (250.9 to 547.8)	176.7 (120.9 to 249.3)	76.3 (48.5 to 115.5)	147.9 (73.2 to 293.8)	50.2 (34.9 to 70.2)	18.1 (12.1 to 25.0)
Mexico	1661.6 (1190.7 to 2220.7)	721.2 (508.7 to 962.0)	157.8 (104.5 to 229.2)	254.6 (174.5 to 356.1)	122.5 (78.2 to 183.8)	266.7 (135.6 to 518.3)	83.2 (57.9 to 116.9)	55.6 (38.8 to 75.6)
Micronesia (Federated States of)	1856.3 (1337.4 to 2445.6)	978.4 (687.2 to 1301.5)	318.4 (208.3 to 456.3)	195.2 (135.5 to 271.7)	115.8 (84.4 to 153.3)	181.9 (90.4 to 365.5)	53.3 (38.7 to 70.6)	13.4 (9.0 to 18.4)
Mongolia	1945.0 (1397.8 to 2571.8)	820.6 (578.5 to 1100.3)	141.3 (91.6 to 203.8)	429.8 (290.6 to 607.7)	237.7 (169.0 to 323.1)	188.2 (95.5 to 377.5)	102.6 (73.0 to 140.0)	24.7 (16.7 to 34.0)
Montenegro	2370.3 (1677.7 to 3182.8)	1106.0 (779.0 to 1482.5)	140.6 (92.1 to 205.6)	610.8 (409.3 to 877.6)	215.0 (138.5 to 325.3)	176.1 (88.7 to 347.8)	101.2 (60.9 to 159.6)	20.6 (13.7 to 28.5)
Morocco	1804.5 (1300.0 to 2380.5)	868.0 (610.1 to 1167.0)	284.0 (188.1 to 414.4)	280.9 (194.4 to 393.4)	121.1 (85.1 to 166.7)	180.5 (90.4 to 357.2)	57.8 (41.3 to 79.9)	12.1 (8.0 to 16.8)
Mozambique	1717.9 (1241.6 to 2252.9)	739.4 (524.2 to 992.0)	113.3 (74.4 to 163.1)	352.8 (248.6 to 483.8)	167.2 (118.3 to 232.1)	193.2 (97.5 to 381.5)	116.9 (83.3 to 162.0)	35.1 (23.7 to 47.8)
Myanmar	1852.4 (1339.2 to 2430.4)	802.9 (570.3 to 1077.1)	380.1 (252.2 to 552.6)	271.0 (194.3 to 365.8)	174.8 (123.7 to 233.8)	135.5 (67.1 to 274.2)	72.7 (50.9 to 101.0)	15.4 (10.2 to 21.1)

© 2022 Chen N et al. *JAMA Network Open.*

Namibia	1419.0 (1024.8 to 1884.1)	684.4 (481.4 to 920.1)	113.5 (75.6 to 161.1)	205.4 (146.6 to 274.5)	91.3 (63.6 to 125.0)	216.5 (110.4 to 422.0)	68.3 (50.0 to 91.6)	39.6 (27.2 to 53.2)
Nepal	1818.5 (1315.3 to 2393.9)	860.8 (612.8 to 1149.9)	139.6 (89.4 to 200.6)	354.5 (249.5 to 482.9)	169.5 (124.9 to 220.7)	173.5 (88.2 to 346.6)	81.3 (59.1 to 107.9)	39.2 (26.6 to 53.0)
Netherlands	2087.7 (1484.6 to 2769.8)	948.6 (658.6 to 1285.9)	322.5 (212.3 to 467.3)	356.9 (241.8 to 508.4)	120.8 (77.2 to 184.1)	245.3 (125.4 to 490.3)	49.6 (30.3 to 78.1)	44.1 (29.8 to 60.1)
New Zealand	2592.4 (1839.6 to 3504.3)	998.6 (693.7 to 1354.1)	86.1 (56.5 to 122.9)	742.3 (497.4 to 1056.9)	276.5 (177.0 to 421.3)	310.3 (156.2 to 621.1)	131.9 (79.5 to 209.1)	46.7 (32.1 to 63.8)
Nicaragua	1767.4 (1282.6 to 2317.4)	780.0 (548.7 to 1044.6)	134.9 (89.1 to 194.4)	324.9 (232.6 to 449.4)	218.1 (147.6 to 315.2)	201.2 (100.6 to 402.7)	70.2 (43.2 to 120.8)	38.1 (25.7 to 51.6)
Niger	1426.8 (1020.3 to 1899.5)	740.0 (521.7 to 987.9)	118.5 (78.8 to 169.6)	202.1 (142.2 to 273.6)	88.5 (64.0 to 117.1)	197.1 (100.1 to 386.7)	71.5 (52.5 to 92.8)	9.0 (6.0 to 12.6)
Nigeria	1579.3 (1132.4 to 2111.4)	881.2 (619.2 to 1184.7)	147.8 (96.3 to 213.1)	192.6 (134.6 to 260.7)	77.8 (55.3 to 103.9)	210.8 (107.3 to 414.6)	60.1 (44.2 to 79.6)	9.0 (6.2 to 12.4)
North Macedonia	2208.7 (1583.2 to 2942.7)	1012.5 (710.5 to 1358.5)	140.2 (91.3 to 202.7)	557.5 (375.6 to 797.9)	206.5 (132.9 to 309.8)	173.4 (87.7 to 341.7)	100.5 (61.7 to 156.2)	18.1 (11.9 to 25.3)

© 2022 Chen N et al. *JAMA Network Open.*

Northern Mariana Islands	1814.2 (1299.2 to 2405.7)	962.5 (676.6 to 1279.7)	318.2 (206.7 to 456.9)	199.1 (135.9 to 280.9)	77.8 (49.7 to 118.1)	189.6 (93.8 to 380.1)	52.6 (36.3 to 73.8)	14.3 (9.4 to 19.9)
Norway	2144.4 (1523.1 to 2875.2)	952.6 (662.8 to 1285.9)	265.2 (174.8 to 380.1)	367.2 (250.5 to 522.2)	124.6 (79.2 to 188.0)	297.5 (150.8 to 595.9)	94.2 (60.0 to 141.3)	43.0 (29.9 to 58.3)
Oman	1711.3 (1218.5 to 2279.3)	812.7 (568.2 to 1092.1)	265.0 (178.8 to 383.8)	304.3 (206.1 to 434.9)	88.1 (57.8 to 130.8)	185.8 (93.0 to 364.3)	42.8 (26.9 to 65.2)	12.6 (8.4 to 17.3)
Pakistan	1502.9 (1083.3 to 1977.3)	647.5 (458.9 to 878.0)	162.9 (107.9 to 236.0)	275.6 (191.4 to 376.2)	147.5 (108.0 to 190.4)	160.1 (80.8 to 315.9)	69.6 (50.6 to 91.6)	39.8 (27.2 to 53.9)
Palestine	2162.9 (1610.0 to 2810.4)	793.3 (560.7 to 1063.7)	281.7 (185.6 to 412.1)	489.5 (351.3 to 685.6)	296.8 (190.2 to 453.3)	178.7 (89.5 to 350.3)	109.7 (58.0 to 223.1)	13.2 (8.8 to 18.2)
Panama	1567.7 (1112.5 to 2074.0)	759.2 (530.3 to 1014.4)	134.7 (89.2 to 193.9)	247.3 (167.5 to 346.8)	139.9 (93.0 to 202.0)	200.9 (100.3 to 404.5)	50.4 (33.4 to 74.7)	35.3 (23.8 to 48.4)
Papua New Guinea	1943.5 (1405.1 to 2559.0)	980.5 (693.1 to 1307.0)	315.4 (206.5 to 449.1)	236.1 (168.1 to 323.0)	178.0 (132.1 to 229.2)	159.4 (78.8 to 321.4)	63.7 (46.4 to 83.4)	10.4 (6.8 to 14.5)
Paraguay	1696.3 (1211.7 to 2256.7)	818.1 (577.3 to 1100.7)	164.3 (107.2 to 238.0)	281.0 (191.3 to 397.1)	143.1 (99.1 to 198.8)	199.0 (99.8 to 399.0)	54.1 (37.7 to 75.6)	36.8 (24.8 to 50.4)

© 2022 Chen N et al. *JAMA Network Open.*

Peru	1481.6 (1066.1 to 1974.6)	684.7 (483.4 to 922.2)	135.6 (89.1 to 195.1)	232.8 (159.2 to 322.5)	140.6 (96.2 to 198.0)	205.9 (103.7 to 414.1)	48.9 (32.9 to 70.7)	33.1 (22.5 to 45.1)
Philippines	2064.6 (1490.8 to 2726.1)	1000.4 (704.9 to 1329.8)	530.1 (350.6 to 764.8)	211.9 (146.5 to 289.6)	125.1 (91.2 to 166.3)	131.3 (65.1 to 263.9)	51.2 (36.9 to 69.6)	14.7 (10.1 to 19.9)
Poland	2459.0 (1749.1 to 3296.1)	1129.4 (793.1 to 1516.1)	165.1 (109.5 to 239.5)	628.1 (422.5 to 895.8)	214.0 (137.0 to 321.3)	184.1 (92.5 to 360.2)	100.8 (61.2 to 157.9)	37.6 (25.9 to 51.5)
Portugal	2231.1 (1593.6 to 2962.4)	1162.7 (826.4 to 1568.4)	357.1 (236.7 to 519.8)	266.5 (180.1 to 379.7)	99.8 (63.9 to 151.9)	273.2 (137.6 to 544.6)	40.2 (24.7 to 62.0)	31.5 (20.9 to 43.2)
Qatar	1747.0 (1255.7 to 2324.3)	839.5 (592.4 to 1132.8)	247.6 (165.9 to 357.4)	316.6 (213.5 to 456.7)	94.8 (61.2 to 142.5)	189.5 (95.4 to 379.6)	45.3 (27.6 to 71.1)	13.7 (9.2 to 19.0)
South Korea	2208.8 (1548.6 to 2999.8)	993.3 (693.9 to 1338.8)	172.0 (112.6 to 247.5)	415.1 (278.9 to 589.8)	162.5 (103.6 to 247.4)	369.4 (186.9 to 729.6)	66.0 (40.4 to 103.4)	30.6 (20.8 to 42.1)
Republic of Moldova	2089.1 (1498.2 to 2778.0)	958.8 (670.7 to 1281.0)	140.8 (92.3 to 203.6)	468.6 (318.6 to 661.5)	191.7 (128.2 to 275.0)	223.0 (114.7 to 442.9)	89.0 (58.6 to 129.2)	17.2 (11.5 to 24.0)
Romania	2393.2 (1702.4 to 3162.7)	1156.9 (807.6 to 1555.6)	140.8 (91.2 to 205.3)	586.3 (394.1 to 840.8)	209.9 (135.2 to 313.9)	177.6 (89.3 to 354.5)	102.4 (63.7 to 159.7)	19.2 (12.7 to 26.3)

© 2022 Chen N et al. *JAMA Network Open.*

Russia	2449.8 (1744.9 to 3288.5)	1023.0 (722.6 to 1368.0)	164.9 (109.6 to 238.4)	615.0 (414.4 to 871.9)	196.7 (130.3 to 288.5)	331.3 (170.0 to 665.5)	96.2 (61.8 to 144.3)	22.8 (15.7 to 30.8)
Rwanda	2020.0 (1447.8 to 2720.9)	749.4 (524.0 to 1008.8)	114.4 (74.6 to 164.9)	450.7 (304.0 to 685.7)	324.5 (188.4 to 584.4)	197.0 (100.1 to 385.1)	146.0 (82.6 to 282.8)	37.8 (25.8 to 52.1)
Saint Lucia	1563.1 (1120.2 to 2068.9)	733.5 (515.0 to 984.2)	134.2 (89.1 to 194.0)	236.7 (161.5 to 331.0)	169.2 (116.0 to 237.9)	203.1 (101.7 to 409.1)	56.6 (39.3 to 80.2)	29.9 (20.0 to 40.6)
Saint Vincent and the Grenadines	1559.5 (1115.2 to 2080.7)	711.1 (498.7 to 949.5)	134.1 (88.3 to 194.2)	241.0 (165.1 to 335.3)	189.8 (133.1 to 262.1)	200.5 (101.1 to 403.3)	61.5 (43.3 to 84.4)	21.7 (14.5 to 30.2)
Samoa	1941.3 (1401.4 to 2548.7)	1034.1 (733.4 to 1379.5)	318.1 (205.9 to 455.2)	205.9 (145.4 to 283.1)	130.2 (94.7 to 171.5)	185.0 (91.0 to 375.5)	54.8 (39.3 to 74.5)	13.2 (8.6 to 18.3)
Sao Tome and Principe	1399.2 (997.0 to 1880.0)	664.1 (467.0 to 893.1)	117.9 (77.4 to 169.5)	228.7 (157.8 to 314.1)	86.9 (61.4 to 117.6)	213.1 (108.6 to 419.9)	78.4 (57.8 to 103.1)	10.0 (6.6 to 13.8)
Saudi Arabia	2106.7 (1503.4 to 2825.3)	803.0 (568.3 to 1071.7)	268.6 (178.9 to 389.4)	569.2 (381.8 to 825.0)	145.1 (95.7 to 213.2)	233.1 (118.8 to 457.8)	74.5 (47.7 to 112.1)	13.2 (8.9 to 18.4)
Senegal	1361.1 (968.6 to 1818.4)	674.3 (473.0 to 909.8)	118.0 (78.9 to 169.3)	201.8 (142.0 to 272.4)	83.4 (60.7 to 110.8)	207.1 (105.2 to 411.2)	67.1 (49.4 to 87.2)	9.6 (6.5 to 13.3)

© 2022 Chen N et al. *JAMA Network Open.*

Serbia	2359.7 (1696.5 to 3133.9)	1126.6 (783.2 to 1515.4)	140.3 (92.6 to 203.5)	579.8 (393.6 to 821.0)	212.8 (137.7 to 314.5)	177.0 (89.2 to 350.0)	100.5 (60.9 to 159.7)	22.8 (15.3 to 31.3)
Seychelles	1669.2 (1195.0 to 2208.1)	818.4 (578.2 to 1100.9)	376.9 (250.4 to 544.5)	181.1 (123.9 to 252.4)	77.2 (50.8 to 113.2)	149.8 (74.7 to 302.6)	53.6 (37.9 to 73.2)	12.2 (8.1 to 16.7)
Sierra Leone	1550.6 (1120.8 to 2050.5)	735.1 (521.0 to 993.2)	117.2 (77.4 to 168.5)	272.9 (197.0 to 368.3)	130.6 (91.5 to 185.5)	201.9 (102.5 to 397.2)	83.8 (60.2 to 115.6)	9.0 (6.0 to 12.7)
Singapore	1864.4 (1305.0 to 2559.9)	787.3 (552.0 to 1073.6)	172.2 (114.0 to 248.7)	347.7 (232.0 to 498.8)	145.6 (91.9 to 223.3)	333.4 (167.3 to 663.3)	57.2 (34.3 to 89.7)	20.9 (13.6 to 29.1)
Slovakia	2451.9 (1758.2 to 3254.5)	1110.7 (779.0 to 1491.1)	140.4 (91.5 to 203.0)	672.8 (451.0 to 963.2)	221.8 (142.7 to 333.9)	178.6 (89.7 to 354.5)	108.1 (65.6 to 170.0)	19.4 (12.9 to 26.7)
Slovenia	2529.5 (1803.6 to 3382.0)	1066.9 (734.8 to 1438.9)	140.1 (90.4 to 202.1)	739.7 (497.4 to 1049.9)	247.9 (160.1 to 370.0)	178.5 (90.0 to 352.2)	119.9 (72.3 to 188.7)	36.5 (25.0 to 49.9)
Solomon Islands	1938.4 (1389.4 to 2553.4)	977.6 (686.8 to 1298.4)	317.6 (208.4 to 455.8)	268.3 (186.5 to 373.8)	127.9 (93.1 to 169.8)	168.4 (84.5 to 342.3)	64.6 (47.4 to 84.7)	14.0 (9.5 to 19.1)
Somalia	1661.7 (1198.1 to 2189.8)	730.5 (517.4 to 977.1)	113.9 (74.6 to 164.4)	294.3 (207.3 to 420.7)	186.0 (128.2 to 272.8)	190.8 (97.2 to 375.3)	106.5 (75.0 to 150.1)	39.8 (27.6 to 54.5)

© 2022 Chen N et al. *JAMA Network Open.*

South Africa	1334.0 (955.6 to 1783.1)	617.6 (434.7 to 829.2)	133.6 (88.2 to 190.6)	169.0 (116.4 to 231.9)	73.7 (50.6 to 103.8)	234.1 (119.2 to 459.6)	59.6 (43.1 to 81.1)	46.5 (32.0 to 63.6)
South Sudan	1710.1 (1244.7 to 2234.2)	724.7 (516.1 to 976.9)	112.1 (73.9 to 160.6)	333.6 (239.5 to 464.0)	210.5 (142.9 to 308.4)	189.1 (96.8 to 371.9)	108.2 (77.5 to 152.8)	31.9 (22.0 to 43.0)
Spain	1950.3 (1402.4 to 2641.6)	824.3 (587.6 to 1128.7)	269.9 (181.1 to 392.6)	368.7 (247.1 to 533.7)	117.7 (75.6 to 178.3)	288.5 (137.8 to 587.6)	51.7 (31.5 to 82.2)	29.5 (20.0 to 39.9)
Sri Lanka	1826.0 (1331.8 to 2391.2)	799.7 (562.8 to 1070.1)	378.7 (250.7 to 554.6)	301.6 (219.6 to 408.4)	141.1 (100.6 to 192.3)	138.5 (68.9 to 278.9)	53.9 (35.0 to 82.2)	12.5 (8.4 to 17.3)
Sudan	1668.5 (1207.2 to 2186.5)	788.3 (553.5 to 1045.1)	282.3 (186.1 to 410.8)	231.7 (164.6 to 315.1)	101.9 (70.7 to 145.0)	175.8 (88.9 to 351.2)	77.2 (54.7 to 106.1)	11.2 (7.6 to 15.6)
Suriname	1590.1 (1142.9 to 2095.4)	725.3 (511.3 to 976.0)	134.2 (88.4 to 194.8)	241.0 (165.0 to 337.1)	195.5 (140.9 to 261.6)	206.4 (103.3 to 414.8)	67.6 (47.1 to 94.2)	20.2 (13.5 to 27.5)
Sweden	2207.6 (1573.6 to 2921.7)	953.0 (666.9 to 1291.2)	434.6 (291.8 to 628.1)	349.2 (236.4 to 498.1)	126.7 (79.4 to 190.7)	202.0 (102.0 to 399.2)	96.8 (61.2 to 147.2)	45.2 (31.1 to 61.6)
Switzerland	2548.6 (1807.2 to 3401.3)	1197.2 (836.4 to 1618.9)	354.7 (231.1 to 511.4)	486.3 (328.4 to 706.9)	146.0 (93.4 to 217.0)	258.6 (130.2 to 513.0)	67.0 (40.6 to 105.3)	38.7 (26.0 to 53.0)

© 2022 Chen N et al. *JAMA Network Open.*

Syrian Arab Republic	2251.8 (1686.9 to 2907.9)	802.3 (559.6 to 1070.6)	285.2 (189.5 to 415.7)	556.9 (394.7 to 786.9)	308.3 (202.2 to 462.9)	180.4 (90.7 to 357.1)	105.5 (50.3 to 231.1)	13.2 (8.7 to 18.4)
Tajikistan	1851.5 (1346.5 to 2427.1)	799.9 (561.5 to 1078.5)	141.0 (90.7 to 203.8)	356.5 (251.2 to 485.2)	257.8 (184.2 to 347.6)	178.7 (91.0 to 356.0)	89.8 (61.8 to 127.7)	27.8 (18.9 to 37.9)
Thailand	1685.3 (1207.3 to 2236.9)	792.7 (556.9 to 1066.9)	381.2 (253.4 to 556.3)	217.4 (147.7 to 308.0)	78.5 (52.3 to 112.0)	157.3 (77.4 to 316.1)	35.4 (23.5 to 51.5)	22.8 (15.4 to 31.5)
Timor-Leste	2068.6 (1509.5 to 2696.9)	822.1 (580.7 to 1094.9)	376.5 (248.2 to 549.8)	365.4 (257.1 to 521.5)	277.3 (179.3 to 430.9)	126.2 (63.0 to 254.2)	89.2 (51.4 to 168.6)	11.9 (7.9 to 16.5)
Togo	1441.4 (1027.9 to 1916.8)	729.7 (511.0 to 990.3)	118.6 (78.2 to 170.6)	218.1 (152.3 to 300.1)	86.5 (62.3 to 114.5)	206.5 (103.9 to 409.0)	72.7 (53.1 to 93.6)	9.2 (6.2 to 12.8)
Tonga	1850.9 (1326.2 to 2452.6)	1016.1 (716.7 to 1359.8)	320.6 (208.1 to 459.1)	167.6 (116.0 to 234.6)	107.9 (77.5 to 145.2)	187.9 (94.0 to 379.1)	38.5 (27.7 to 51.8)	12.5 (8.3 to 17.4)
Trinidad and Tobago	1529.2 (1092.0 to 2031.2)	719.6 (508.4 to 963.4)	134.2 (87.9 to 194.8)	222.5 (151.0 to 311.1)	157.8 (110.2 to 220.8)	207.6 (104.8 to 420.8)	51.9 (35.9 to 72.3)	35.6 (24.1 to 48.4)
Tunisia	1632.0 (1172.3 to 2163.2)	791.5 (553.5 to 1052.4)	286.0 (188.0 to 415.1)	237.9 (161.3 to 339.6)	79.8 (52.7 to 117.8)	183.0 (92.6 to 368.2)	40.2 (26.0 to 60.3)	13.6 (8.7 to 19.0)

© 2022 Chen N et al. *JAMA Network Open.*

Turkey	1810.9 (1295.9 to 2386.9)	953.6 (671.3 to 1283.5)	284.8 (188.6 to 414.8)	241.7 (165.8 to 341.7)	88.2 (59.5 to 128.2)	175.7 (86.7 to 352.9)	38.1 (24.1 to 58.1)	28.8 (20.2 to 38.6)
Turkmenistan	1752.1 (1261.5 to 2325.8)	826.4 (582.0 to 1109.2)	141.3 (91.8 to 204.3)	313.1 (211.7 to 444.8)	199.8 (135.8 to 283.1)	179.5 (91.1 to 354.0)	73.6 (50.5 to 104.1)	18.5 (12.4 to 25.8)
Uganda	1558.0 (1128.3 to 2056.3)	724.7 (507.8 to 977.4)	114.1 (74.2 to 164.6)	268.8 (191.0 to 365.1)	135.9 (95.5 to 184.2)	195.3 (99.3 to 386.5)	87.1 (62.2 to 119.8)	32.1 (22.1 to 43.8)
Ukraine	2377.5 (1698.5 to 3167.3)	1086.1 (763.8 to 1449.1)	165.3 (110.0 to 237.9)	600.3 (402.0 to 863.5)	204.2 (134.6 to 301.5)	207.6 (106.0 to 413.3)	98.7 (64.5 to 145.9)	15.2 (10.1 to 20.8)
United Arab Emirates	1702.9 (1218.1 to 2248.0)	798.9 (559.5 to 1067.8)	250.7 (166.5 to 364.1)	291.7 (197.8 to 412.5)	109.3 (75.3 to 152.1)	183.5 (92.6 to 362.8)	56.7 (39.6 to 79.6)	12.1 (8.0 to 16.6)
United Kingdom	2422.0 (1725.6 to 3199.1)	1108.1 (774.6 to 1494.6)	446.8 (302.0 to 636.9)	358.7 (242.0 to 513.5)	124.2 (79.3 to 190.0)	275.4 (139.4 to 545.2)	57.7 (35.3 to 90.0)	51.1 (35.6 to 68.5)
United Republic of Tanzania	1447.2 (1035.4 to 1923.6)	702.6 (490.5 to 941.3)	114.0 (75.4 to 163.6)	206.7 (143.5 to 283.6)	110.4 (77.4 to 154.3)	201.0 (101.6 to 395.6)	76.7 (56.4 to 100.7)	35.9 (24.8 to 49.8)
United States of America	2856.5 (2067.5 to 3783.0)	1402.9 (1007.0 to 1852.3)	500.3 (338.9 to 704.9)	334.3 (228.4 to 466.7)	113.1 (72.9 to 170.3)	378.1 (194.8 to 759.8)	84.0 (53.8 to 124.4)	43.8 (30.7 to 58.5)

© 2022 Chen N et al. *JAMA Network Open.*

Uruguay	2036.8 (1442.6 to 2741.5)	807.6 (564.7 to 1087.3)	192.6 (126.1 to 276.7)	420.5 (283.8 to 596.4)	195.7 (128.2 to 289.6)	301.9 (154.3 to 609.2)	88.5 (57.6 to 132.1)	30.0 (20.2 to 41.6)
Uzbekistan	1795.8 (1291.5 to 2379.9)	814.7 (575.7 to 1089.9)	141.2 (92.2 to 203.2)	335.3 (226.3 to 475.2)	184.6 (125.6 to 260.9)	191.5 (96.8 to 386.1)	72.4 (49.9 to 102.2)	56.0 (38.1 to 76.0)
Vanuatu	1991.7 (1434.5 to 2623.2)	1097.0 (778.3 to 1462.0)	317.1 (206.9 to 457.0)	191.5 (135.0 to 261.8)	143.1 (106.4 to 183.5)	170.8 (84.9 to 343.5)	60.4 (43.7 to 79.5)	11.8 (7.9 to 16.3)
Venezuela (Bolivarian Republic of)	1655.9 (1185.3 to 2200.8)	746.1 (518.3 to 997.0)	135.1 (88.9 to 195.5)	295.3 (203.4 to 413.2)	173.8 (117.6 to 247.2)	189.7 (93.3 to 387.3)	68.1 (45.4 to 100.5)	47.8 (33.1 to 63.6)
Viet Nam	1829.2 (1307.0 to 2428.4)	896.1 (634.2 to 1202.7)	381.9 (252.0 to 557.7)	249.3 (171.1 to 349.8)	107.4 (74.2 to 148.6)	132.9 (65.2 to 265.4)	47.2 (32.6 to 65.8)	14.5 (9.8 to 20.1)
Yemen	1900.0 (1377.0 to 2476.7)	807.2 (563.7 to 1075.2)	282.6 (186.5 to 408.3)	345.1 (244.7 to 474.9)	216.9 (151.6 to 305.1)	163.7 (82.1 to 326.0)	74.6 (48.8 to 115.3)	10.0 (6.5 to 13.7)
Zambia	1399.1 (996.2 to 1856.6)	685.5 (480.0 to 915.4)	113.1 (74.4 to 163.2)	190.8 (131.8 to 262.9)	96.8 (65.8 to 134.9)	197.9 (100.8 to 390.1)	73.7 (54.2 to 95.8)	41.3 (28.4 to 55.3)
Zimbabwe	1375.3 (979.3 to 1843.9)	742.0 (521.6 to 1003.6)	113.7 (75.0 to 162.9)	149.6 (103.3 to 204.3)	76.9 (53.7 to 104.8)	212.2 (108.3 to 418.6)	63.4 (46.9 to 82.5)	17.4 (12.0 to 24.0)

© 2022 Chen N et al. *JAMA Network Open.*

eTable 7. Musculoskeletal Rehabilitation Needs and Trends Between 1990 and 2019 by Health Condition

	Prevalence					YLDs				
	1990		2019		1990-2019	1990		2019		1990-2019
Health condition	No (95% UI)	ASRs per 100 000 persons (95% UI)	No (95% UI)	ASRs per 100 000 persons (95% UI)	EAPC (95% CI)	No (95% UI)	ASRs per 100 000 persons (95% UI)	No (95% UI)	ASRs per 100 000 persons (95% UI)	EAPC (95% CI)
Musculoskeletal disorders	1060596311 (1009078451 to 1116444693)	23010.8 (21966.7 to 24159.9)	1713557313 (1632382721 to 1800359391)	20978.0 (20006.3 to 22052.7)	-0.34 (-0.37 to -0.31) *	93854869 (67677171 to 123557400)	2055.2 (1489.0 to 2706.2)	149003879 (107516947 to 198581644)	1821.9 (1317.1 to 2426.1)	-0.42 (-0.51 to -0.32) *
Low back pain	386004927 (342715789 to 434483554)	8341.2 (7389.8 to 9370.2)	568444532 (505000665 to 640597792)	6972.5 (6190.5 to 7860.5)	-0.52 (-0.57 to -0.47) *	43361648 (30529532 to 57934973)	932.5 (658.6 to 1248.3)	63685120 (44999198 to 85192922)	780.2 (549.3 to 1046.1)	-0.52 (-0.66 to -0.37) *
Neck pain	124472266 (98829383 to 157046441)	2709.4 (2172.3 to 3409.8)	222718453 (179236777 to 281066237)	2696.5 (2177.0 to 3375.2)	-0.10 (-0.18 to -0.02) *	12393478 (8128866 to 17740324)	268.3 (176.7 to 382.7)	22081323 (14508244 to 31726933)	267.3 (175.5 to 383.5)	-0.09 (-0.35 to 0.16)
Fractures	258201535 (243329783 to 275614557)	5777.9 (5458.7 to 6158.7)	436288796 (411003613 to 464527319)	5379.5 (5069.4 to 5720.2)	-0.35 (-0.41 to -0.30) *	15535133 (10650550 to 21447363)	346.2 (237.9 to 478.6)	25705426 (17716628 to 35606674)	317.3 (218.7 to 439.7)	-0.42 (-0.65 to -0.19) *

© 2022 Chen N et al. *JAMA Network Open.*

Other injuries	213951206 (197805240 to 235079320)	4518.4 (4184.0 to 4962.6)	305001551 (281550649 to 335914599)	3757.2 (3472.0 to 4136.7)	-0.75 (-0.82 to -0.68) *		8501998 (6074167 to 11411839)	172.5 (123.0 to 231.7)	10629607 (7509804 to 14577882)	131.8 (93.0 to 180.4)	-1.04 (-1.38 to -0.71) *
Osteoarthritis	160587859 (128204391 to 193028735)	4018.4 (3216.6 to 4829.5)	343943653 (274662464 to 414306891)	4141.1 (3310.3 to 4987.2)	0.13 (0.06 to 0.19) *		8834777 (4495198 to 17359205)	220.7 (112.5 to 433.5)	18948965 (9571298 to 37659660)	228.0 (115.3 to 452.7)	0.14 (-0.14 to 0.42)
Amputation	115511394 (106982929 to 125220027)	2489.0 (2311.3 to 2688.9)	175731751 (163639977 to 189776551)	2167.0 (2017.9 to 2336.8)	-0.64 (-0.73 to -0.55) *		4040973 (2939365 to 5526081)	88.0 (63.7 to 120.0)	5520398 (3835581 to 7705268)	68.1 (47.3 to 94.9)	-1.13 (-1.60 to -0.65) *
Rheumatoid arthritis	6546378 (5928082 to 7223937)	150.6 (136.8 to 165.9)	13487835 (12218038 to 14875080)	162.8 (147.6 to 179.5)	0.37 (0.04 to 0.70) *		1186862 (821206 to 1596157)	27.1 (18.8 to 36.4)	2433040 (1681354 to 3276916)	29.3 (20.3 to 39.5)	0.38 (-0.40 to 1.16)

Abbreviations: ASRs, age-standardized rates; EAPC, estimated annual percentage change; UI, uncertainty interval.

*The annual percentage change in the age- standardized rate did not include 0.

© 2022 Chen N et al. *JAMA Network Open.*
